# Epidemiology of Four Major Canine Tumours in the UK: Insights From a National Pathology Registry With Comparative Oncology Perspectives

**DOI:** 10.1111/vco.70056

**Published:** 2026-02-24

**Authors:** José Rodríguez, Antonio Espinosa de los Monteros, Ángelo Santana, P. J. Noble, Alan D. Radford, Francesco Cian, Annika Herrmann, Jenny S. McKay, Matthew Jones, David R. Killick

**Affiliations:** ^1^ Institute for Animal Health and Food Safety University of Las Palmas de Gran Canaria Las Palmas Spain; ^2^ Institute of Infection, Veterinary and Ecological Sciences University of Liverpool Neston UK; ^3^ Mathematics Department University of Las Palmas de Gran Canaria Las Palmas Spain; ^4^ Batt Laboratories, Venture Centre Coventry UK; ^5^ Veterinary Pathology Group (VPG) Exeter UK; ^6^ IDEXX Laboratories Wetherby West Yorkshire UK

**Keywords:** breed risk, canine cancer, comparative oncology, haemangiosarcoma, mast cell tumour, melanoma, one health, osteosarcoma, tumour registry, veterinary oncology

## Abstract

Pet dogs with naturally occurring cancers provide valuable models for comparative oncology and pathology tumour registries offer a powerful resource for onco‐epidemiological research. Here, we analysed the Small Animal Veterinary Surveillance Network (SAVSNET) pathology‐based tumour registry (PTR), one of the largest veterinary tumour registries to date, focusing on four major canine tumours: mast cell tumour (MCT), osteosarcoma (OSA), haemangiosarcoma (HSA), and melanoma (MEL). A case–control study was conducted using a subset of 130 998 histologically confirmed tumours drawn from the pathology tumour registry containing over 1.1 million canine tumour records from 1.02 million dogs collected in the UK between 2010 and 2023. Case–control analyses were performed for melanoma, haemangiosarcoma, OSA and MCT using a denominator population of dogs attending first‐opinion veterinary practices. Additionally for MCT, comparisons were made against dogs diagnosed with other tumour types (‘tumour denominator’ approach). Breed‐specific risks were identified, including high odds ratios (OR) for Bulldog‐related breeds and Retrievers with mast cell tumours (MCTs) (OR up to 6.8, 95% CI 6.0, 7.6), Rottweilers, Shar Pei and Giant‐Schnauzer with melanoma (OR up to 50.3, 95% CI 24.7, 102.5), and German Shepherd Dogs, Mastiffs, and Bullmastiffs with haemangiosarcoma (OR up to 28.0, 95% CI 10.6, 40.9). Higher‐grade MCT were diagnosed at older ages and certain breeds were more predisposed to higher‐grade MCT (Shar Pei, Rottweiler) while others were more prone to low‐grade MCTs (Boxer, Boston Terrier). Neutered dogs generally had higher tumour odds than entire dogs; for example, in MCTs, female‐neutered vs. female‐entire showed OR 1.81 (95% CI 1.6, 2.0) in ages 3–6, with similar patterns across older age bands. In general, the difference in OR values between entire and neutered dogs was consistently more pronounced in females than in males. These findings demonstrate the value of the SAVSNET PTR as a comprehensive resource for canine tumour surveillance, with potential to support health initiatives and cancer research by identifying breed‐specific and demographic risk factors as well as a foundational tool for comparative cancer epidemiology.

## 1 Introduction

For more than 10 000 years, dogs and humans have co‐evolved [1], thriving in shared environments and facing similar challenges, such as food scarcity, hostile climates, a diverse range of infectious diseases, and more recently, chemical pollution [2].

There is increasing evidence that the narrowing of genetic heterogeneity within dog breeds alongside the shared environment of humans and dogs makes several spontaneous canine cancers occurring in immunocompetent free‐living pet dogs an invaluable comparative tool for cancer investigations [1]. For example, canine haemangiosarcoma(HSA), which mirrors human angiosarcoma, occurs more frequently in dogs, making it a strong model for understanding disease mechanisms and evaluating novel treatments. In osteosarcomas (OSAs), large‐breed dogs have provided critical insights into tumour biology and drug development that would be difficult to achieve in human studies. The increasing use of canine models in biomarker discovery and targeted therapy validation highlights their growing role in translational cancer research [3].

Cancer in pet dogs is also a problem with its own societal relevance. There are now millions of pet dogs worldwide that provide a variety of benefits to their owner families [4]. Cancer is now recognised as the most frequent cause of death in pet dogs, both in Europe [5, 6] and the US [7] and the commercial Veterinary Oncology market for companion animals is estimated at USD 1.18 billion in 2023 [8].

Recognising One Health Opportunities, the Global Initiative for Veterinary Cancer Surveillance (GIVCS) was launched in 2018 [9] to promote international collaboration in developing and linking cancer registries worldwide [9]. In 2021, the first version of the Small Animal Veterinary Surveillance Network (SAVSNET) pathology‐based tumour registry (PTR) was published [10]. This included more than 100 000 tumours identified by applying rule‐based text‐mining techniques to more than 180 000 electronic pathology records (EPR) pertaining to UK dogs and cats seen in veterinary practice and sampled during routine veterinary practice and diagnosed between April 2018 to June 2019.

Although large in size, the utility of this PTR was limited by its short duration of coverage. In this study, we developed a much larger PTR, using an expanded collection of EPRs received by SAVSNET over the last decade to provide a dataset of more than a million canine and feline tumours. To our knowledge, this is the largest dataset of its kind at the time of writing.

Concomitantly, we have developed tools to enhance the robustness of data accrual and processing in near real‐time to enhance the usability for surveillance. We also established a relevant denominator database derived from the electronic health records (EHRs) of pet dogs attending UK first‐opinion veterinary practices [11].

To test these enhanced functionalities, in this study, we provide an analysis of four tumour types. The first three, melanomas, osteosarcoma, and haemangiosarcoma, are tumours for which the dog tumour has been proposed as a One Medicine model for their human counterparts [1, 2]. The fourth, mast cell tumours, are the most frequently diagnosed malignant tumours in dogs [12] and are therefore of significant veterinary interest.

Canine melanomas (MEL), especially those located in the oral cavity, digits, and uveal tract, are highly relevant models of non‐UV‐induced melanoma in humans. Unlike the more common UV‐induced cutaneous melanomas frequently diagnosed in fair‐skinned humans, these subtypes are rarer, carry poorer prognoses, and are more prevalent in individuals with darker pigmentation [13, 14]. Dogs spontaneously develop melanomas at these anatomical sites more frequently, often with breed‐specific predispositions, providing an opportunity to investigate both somatic mutations and germline risk factors [13, 14].

Canine haemangiosarcoma (HSA) is a common and aggressive cancer of endothelial origin [15] that closely mirrors the human counterpart, angiosarcoma (AS) [16], which is a rare but aggressive malignancy. While AS present's challenges for research due to its low incidence and clinical heterogeneity, dogs spontaneously develop HSA at much higher frequencies, making them valuable models for translational studies [17]. Molecular profiling has revealed shared driver mutations such as NRAS (neuroblastoma RAS viral oncogene homolog), TP53 (tumour protein p53), or KIT (KIT proto‐oncogene receptor tyrosine kinase), along with activation pathways like the PI3K pathway in both species [17].

Osteosarcoma (OSA) is the most common primary bone tumour in both dogs and humans. While it is a rare malignancy in humans, particularly affecting adolescents and young adults, it is relatively common in large and giant breed dogs. Canine OSA shares key biological, molecular, and clinical features with human OSA, including aggressive behaviour, early metastasis, and recurrent mutation or copy number alteration of TP53 [18, 19].

These parallels in histopathology, molecular features, and clinical behaviour across tumour types position canine cancers such as haemangiosarcoma, melanoma, and OSA as robust spontaneous models for human disease, playing a key role in testing immunotherapies and novel targeted treatments, investigating risk factors like body size and replicative stress, and accelerating the discovery of molecular targets and predictive biomarkers for improved translational outcomes, particularly in paediatric and rare human cancers [13, 17, 18].

Mast cell tumours (MCTs) are the most commonly diagnosed malignant skin neoplasm in dogs, accounting for a significant proportion of all canine tumours across multiple studies and regions, including the UK [20], Germany [21], Switzerland [12], Italy [22], Denmark [23], Portugal [24], Spain [25], the US [26], South Korea [27] and Australia [28].

Although MCTs are not established models to specific human tumours, they are recognised to be of comparative interest due to shared molecular features in relation to mutations in the KIT Proto‐Oncogene Receptor Tyrosine Kinase (KIT) [29, 30].

The aim of this study was to characterise these four major tumour types in the canine population using data from the SAVSNET pathology tumour registry (PTR). By leveraging this large‐scale dataset, we aimed to describe epidemiological patterns by breed, age, sex, and neuter status.

More specifically, the aims of this study were to: (a) evaluate the frequency of four major tumour types: MCT, MEL, HSA, and OSA in the SAVSNET PTR and assess temporal trends in their diagnosis over the study period; (b) provide a descriptive analysis of each tumour type, including anatomical distribution and age at diagnosis, and explore associations between histological grade and age or breed in the case of MCT; (c) investigate breed‐specific odds of tumour diagnosis using multivariable models adjusted for age, sex, and neuter status; (d) assess the influence of neuter status across sex and age groups on the odds of tumour occurrence, with a focus on sex‐hormone–sensitive patterns.

## 2 Materials and Methods

SAVSNET receives veterinary data from two main sources: veterinary clinicians (vet data) and diagnostic laboratories (lab data).

The laboratory data includes pathology reports (histologies and cytologies) identified and classified under the label ‘Pathologist Report’. The current version of the PTR contains cytology and histopathology reports submitted between January 2010 to December 2023 from three UK veterinary laboratories (IDEXX Laboratories, the Veterinary Pathology Group (VPG) and Batt Laboratories Ltd).

For the PTR, the first step involves filtering pathology reports from SAVSNET lab data and, from this filtered dataset, only reports that contain a diagnosis of cancer (inclusion criteria) within the diagnosis/interpretation section of the report are retained.

In this sense, a tumour diagnosis was assigned when an exact text string match was detected in the diagnosis/interpretation section of the electronic pathology report (EPR). This matching process was conducted using Python (PY) scripts (version 3.12.7) (described in the Supporting Information [PY script] in Data S2) which identify terms from a tailored list based on the Vet‐ICD‐O Canine‐1 [31] and, complementary, sources such as ‘Tumours of Domestic Animals’ [32].

Beyond identifying tumour diagnosis, the PY scripts performed additional data processing and cleaning operations to generate the final normalised dataset including standardised terminology for anatomical locations, grades, and breeds. It also removed false‐positive narratives (such as reports containing expressions such as ‘*no evidence*’, ‘*no neoplas*’, ‘*no tumo*’, ‘*no remain*’ or ‘*inconclusive*’).

For this study, we included a canine subset of the PTR comprised of mast cell tumours (MCTs), melanomas (MEL), haemangiosarcomas (HSA) and OSAs confirmed by histology (Table 1). Reports including any of these four diagnoses along with an additional alternative differential diagnosis such as for example, ‘chondrosarcoma vs chondroblastic osteosarcoma’ or ‘dermal melanoma, most likely benign (syn. melanocytoma)’ were excluded.

**TABLE 1:** Number of mast cell tumours, melanomas, haemangiosarcomas, and osteosarcomas identified during the study period, and numbers of dogs included in the case–control analyses.

Tumour type	Tumours (*n*)	% of all reported tumours
Descriptive analysis		
Mast cell tumour	97 879	13.2%
Melanoma	16 964	2.3%
Haemangiosarcoma	11 308	1.5%
Osteosarcoma	4847	0.7%
	130 998	

Tumour type	Cases (dogs, *n*)	Controls (dogs, *n*)*
Case–control analyses		
Mast cell tumour	24 735	83 365
Oral melanoma	3889	88 382
Cutaneous melanoma	2059	87 966
Cutaneous digital melanoma	835	62 731
Uveal melanoma	644	69 409
Splenic haemangiosarcoma	2380	79 858
Cutaneous haemangiosarcoma	1820	82 686
Osteosarcoma	2166	83 131
	38 528	
Mast cell tumour (univariable tumour‐denominator model)	68 235	440 366

*Note*: The descriptive analysis includes all records of the four tumour types under study (*n* = 130 998). The case–control analyses comprised a main multivariable model, performed for all tumour types using dogs attending first‐opinion practices as the reference population, and a complementary univariable tumour‐denominator model, conducted only for mast cell tumours and using dogs diagnosed with other tumour types as the control population.

For exploratory analysis, MEL and HSA cases were stratified according to their more common anatomical locations, as follows: (1) Oral MEL (those found within the oral cavity); (2) Cutaneous MEL (those located on the haired skin); (3) Cutaneous digital MEL (those located on the digits, toes, claws, nail bed, footpad, phalanges, or ungual regions) [13]; and (4) Uveal MEL (located within the eye and adnexal tissues). Haemangiosarcomas were classified as follows: (1) Splenic HSA (those found in the spleen); and (2) Cutaneous HSA (located in the external parts of the body).

We then conducted a case–control study to evaluate the role of breed, age, sex, and neuter status as risk factors for each of the cancers under study. For this study we used a dog population comprised of dogs presented to 73 UK first opinion veterinary premises (vet data) between the period January 2014 and December 2023 as a reference\control population.

The case group included dogs diagnosed with at least one of the tumours under study, ensuring that multiple diagnoses of the same tumour type in a single individual (for instance, two MCTS) were counted as one case.

To prevent overlap between cases and controls, we generated a unique identifier (Dog_ID) for each dog in both the PTR dataset and vet data, using date of birth (DOB), breed, vet‐postcode, and sex, formatted as follows:

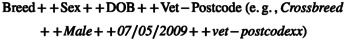




Records with missing values for any of these variables were removed. For each tumour type, a specific denominator population was constructed by excluding dogs whose Dog_ID matched cases of that particular tumour. The final cases included on the case–control analysis are shown in Table 1.

Statistical analyses were performed using R software (version 4.4.1). Categorical variables were summarised as numbers and percentages, and age was expressed as median and interquartile range (IQR) although only for preliminary assessments of comparability between cases and controls, Welch's *t*‐test was used to compare mean ages.

Normality was firstly assessed using the Anderson‐Darling test for datasets with more than 5000 observations and the Shapiro–Wilk test for smaller samples. For comparisons between more than two groups, we used the Kruskal‐Wallis test, followed by post hoc pairwise comparisons using the Conover test when significant differences were detected. For two‐group comparisons, the Wilcoxon rank‐sum test (Mann–Whitney U test) was used. Associations in contingency tables were assessed using the Chi‐square test. Additionally, changes in the relative proportion of tumour types over time were analysed using the two‐sample proportion test, comparing the frequency of each tumour type at the beginning and end of the study period.

Logistic regression (binomial family) was performed using the glm() function from base R (stats package) to assess the association between dog breed and the odds of tumours. The main multivariable model included breed (restricted to breeds with at least five individuals and at least as many controls as cases), age group, and sex/neuter status (four categories: female neutered, female entire, male neutered, and male entire), with an interaction term between sex and age group model <− glm(group ~ Breed + Sex*groupedAge, data = study_data, family = ‘binomial’).

To minimise age‐related confounding, individuals younger than 2 and older than 18 years were excluded, which reduced the mean age difference between cases and controls to < 1 year (cases: 8.55 ± 2.60; controls: 7.43 ± 3.61; Welch's *t*‐test, *p* < 0.0001). For further direct comparisons between sex and neuter status combinations, estimated marginal means (EMMs) were computed on the link scale within each age group using the emmeans() function, and post hoc pairwise contrasts were obtained via Tukey's adjustment.

Because the predefined inclusion criteria (≥ 5 individuals per breed and at least as many controls as cases) excluded some well‐documented high‐risk breeds for MCT (e.g., there were 17 691 Boxer cases, 6616 French Bulldog cases, and 7900 Pug cases in the tumour group, but only 1079 Boxers, 577 French Bulldogs, and 795 Pugs in the control population), we therefore implemented an alternative approach frequently applied in cancer‐registry studies in the absence of suitable population denominators [33], performing a complementary univariable tumour‐denominator analysis restricted to this tumour type. This model was restricted to dogs diagnosed with tumours and breeds with at least five individuals. The binary outcome was the diagnosis of MCT versus other tumour types, using the total number of tumours affecting each breed as the denominator instead of animals attending veterinary practices.

For both analyses, crossbreeds were used as the reference category. In this sense, dogs with a clearly specified breed were classified accordingly while any combination or mixed‐breed descriptions were categorised as crossbreeds, and dogs with no breed information were labelled as NA and excluded from analyses involving breed. In all tests, exact *p*‐values and 95% confidence intervals (CIs) were reported.

## 3 Results

The frequency of diagnosis of each tumour increased during the study period (Supporting Information Figure S1) but the mean number of diagnoses per affected dog for each tumour type over the study period remained stable (Supporting Information Table S1) with MCTs showing the highest average number of diagnoses (1.10), followed by HSAs (1.025), MELs (1.033) and OSAs, which have the lowest mean (1.011). The 95% CI across all tumour types ranged from 1.011 to 1.10 suggesting that for the majority of dogs under study only one tumour was reported with minimal evidence of multiple simultaneous diagnoses.

Additionally, slight but significant changes in the relative proportion of MCTs, HSAs, and OSAs occurred from January 2010 to December 2023 (*p* < 0.05, proportion test), as shown in Table 2, while proportions of MELs remained stable.

**TABLE 2:** Changes in relative proportion of tumour types over time.

	2010	2023	Difference (95% CI)	*p*
Mast cell tumour	12.81%	13.56%	0.75% (0.29%, 1.22%)	0.002
Melanoma	2.27%	2.41%	0.14% (−0.08%, 0.35%)	0.215
Haemangiosarcoma	1.65%	1.45%	−0.20% (−0.38%, −0.03%)	0.019
Osteosarcoma	0.67%	0.50%	−0.17% (−0.28%, −0.06%)	0.001

## 4 Mast Cell Tumours

MCTs predominantly affected the skin (*n* = 86 087; 91%) while the oral cavity (*n* = 1831; 1.9%) and lymph node (*n* = 1629; 1.7%) were the most predominant non‐cutaneous locations (Supporting Information Table S2a,b).

Concerning grades of MCT, from those with available information for the Patnaik's (3‐tier) histological grading system (Supporting Information Table S3), 90.1% were graded as Grade 2, followed by Grade 1 and Grade 3, while for the Kiupel's (2‐tier) approach, the relation for High‐Grade and Low‐Grade was 15.5% to 84.5% respectively (Supporting Information Table S4). MCT were generally diagnosed in the latter part of middle age (8 years IQR: 6, 10) (Supporting Information statistical analysis for MCT) with ranges (6–9) and (9–12) showing the largest odds when compared with the reference group (3–6) years (Supporting Information Table S5).

Additionally, higher grades MCTs were significantly more common in older dogs across both Patnaik and Kiupel histologic grading systems (Supporting Information statistical analysis for MCT in Data S2).

For the Patnaik approach, the Kruskal‐Wallis test revealed a significant difference in age across the three grades (χ^2^ = 711.69, df = 2, *p* < 0.0001) and post hoc Conover's test confirmed that all pairwise comparisons were statistically significant (Grade 2 vs. Grade 1, Grade 3 vs. Grade 1, and Grade 3 vs. Grade 2, all *p* < 0.0001) (Supporting Information statistical analysis for MCT in Data S2).

For those graded as High or Low grade (Kiupel approach), the Wilcoxon rank‐sum test confirmed a statistically significant difference between both groups (W = 187 740 287, *p* < 0.0001), indicating that high‐grade MCTs are more frequently diagnosed in older dogs (Supporting Information statistical analysis for MCT in Data S2).

When it comes to breeds, Hungarian Vizsla (OR: 2.61 [2.20, 3.10]), Irish Terrier (OR: 2.20 [1.54, 3.17]), and Labrador Retriever (OR: 2.12 [2.03, 2.22]) showed greater odds for MCT, while Cavalier King Charles Spaniel (OR: 0.025 [0.02, 0.04]) and Poodle (Toy) (OR: 0.07395 [0.03, 0.16]) had the lowest (Supporting Information Table S5).

Additionally, in Table 3, when analysing ORs of MCT in contrast with other tumour diagnoses across different breeds (complementary univariable tumour‐denominator model), bulldog‐related breeds such as Boston Terrier, Staffordshire Bull Terrier and Boxer, along with Retrievers (Curly Coated, Nova Scotia Duck Tolling, Golden and Labrador) and Shar Pei showed significantly greater odds when compared with Crossbreed dogs.

**TABLE 3:** Breeds with significantly increased odds of mast cell tumour diagnosis compared with crossbreeds from the complementary univariable tumour‐denominator model.

Breed	OR	95% CI	*p*	*n* (%) (Controls)	*n* (%) (Cases)
Boston terrier	6.8	[6.0, 7.6]	< 0.001	651 (0.1)	589 (0.9)
Retriever (curly coated)	5.9	[4.1, 8.6]	< 0.001	62 (0.0)	49 (0.1)
Hungarian wirehaired vizsla	5.2	[2.0, 13.7]	< 0.001	10 (0.0)	7 (0.0)
Shar pei	3.8	[3.4, 4.3]	< 0.001	883 (0.2)	452 (0.7)
Boxer	3.7	[3.6, 3.8]	< 0.001	13 746 (3.1)	6834 (10.0)
Staffordshire bull terrier	3.6	[3.5, 3.7]	< 0.001	23 617 (5.4)	11 301 (16.6)
American bulldog	3.5	[2.5, 4.8]	< 0.001	108 (0.0)	50 (0.1)
Pit bull terrier	3.3	[1.3, 7.9]	0.009	16 (0.0)	7 (0.0)
Spaniel (sussex)	3.2	[2.1, 4.8]	< 0.001	77 (0.0)	33 (0.0)
Weimaraner	3	[2.8, 3.3]	< 0.001	2352 (0.5)	954 (1.4)
Retriever (nova scotia duck tolling)	2.9	[1.6, 5.4]	< 0.001	36 (0.0)	14 (0.0)
American staffordshire terrier	2.8	[2.0, 3.7]	< 0.001	152 (0.0)	56 (0.1)
Pharaoh hound	2.5	[1.1, 5.9]	0.037	21 (0.0)	7 (0.0)
French bulldog	2.4	[2.2, 2.5]	< 0.001	4749 (1.1)	1512 (2.2)
Retriever (golden)	2.3	[2.2, 2.4]	< 0.001	11 952 (2.7)	3670 (5.4)
Spaniel (irish water)	2.3	[1.6, 3.1]	< 0.001	152 (0.0)	46 (0.1)
Bullmastiff	2.2	[1.9, 2.5]	< 0.001	1206 (0.3)	353 (0.5)
Retriever (labrador)	2.1	[2.1, 2.2]	< 0.001	52 471 (11.9)	15 062 (22.1)
Retriever (generic)	2.1	[1.9, 2.3]	< 0.001	2161 (0.5)	610 (0.9)
Canaan dog	1.9	[0.8, 4.6]	0.172	24 (0.0)	6 (0.0)
Manchester terrier	1.9	[1.3, 2.7]	< 0.001	140 (0.0)	35 (0.1)
Mastiff	1.7	[1.4, 2.0]	< 0.001	750 (0.2)	169 (0.2)
Bulldog	1.6	[1.5, 1.7]	< 0.001	4027 (0.9)	845 (1.2)
Bull terrier	1.6	[1.4, 1.7]	< 0.001	1941 (0.4)	404 (0.6)
Irish terrier	1.4	[1.1, 1.8]	0.004	424 (0.1)	81 (0.1)
New zealand huntaway	1.4	[0.5, 3.6]	0.506	27 (0.0)	5 (0.0)
Italian greyhound	1.4	[0.9, 2.1]	0.165	138 (0.0)	25 (0.0)
Bernese mountain dog	1.3	[1.1, 1.6]	0.006	790 (0.2)	137 (0.2)
Goldendoodle	1.3	[0.5, 3.3]	0.602	29 (0.0)	5 (0.0)
Beagle	1.2	[1.1, 1.3]	< 0.001	5654 (1.3)	927 (1.4)
Unknown	1.2	[0.7, 2.0]	0.431	110 (0.0)	18 (0.0)
Hungarian vizsla	1.2	[1.1, 1.3]	< 0.001	2778 (0.6)	447 (0.7)
Maltese	1.2	[1.0, 1.5]	0.120	638 (0.1)	101 (0.1)
Jack russell terrier	1.1	[1.1, 1.2]	< 0.001	18 683 (4.2)	2795 (4.1)
Norwich terrier	1.1	[0.5, 2.1]	0.857	63 (0.0)	9 (0.0)
Parson russell terrier	1	[0.8, 1.4]	0.792	388 (0.1)	54 (0.1)
Great dane	1	[0.8, 1.3]	0.774	724 (0.2)	100 (0.1)
Norfolk terrier	1	[0.7, 1.4]	0.971	334 (0.1)	45 (0.1)
Crossbreed	1			76 258 (17.3)	10 214 (15.0)

*Note*: Reference category for the breed variable: crossbreed (OR = 1). Model restricted to dogs diagnosed with tumours. Complete logistic regression results are provided in Supporting Information Table S6.

Certain breeds showed a clear tendency toward either higher or lower grade MCT forms. For instance, according to the Patnaik grading scheme (Supporting Information Table S7), crossbreed dogs (*n* = 7992) were assigned Grade 3 with a frequency of 4.9% while Gordon Setter (*n* = 14), Leonberger (*n* = 17), and Shar Pei (*n* = 368) had Grade 3 tumours in 42.9%, 29.4%, and 23.1% of cases, respectively.

Under the Kiupel grading system (Supporting Information Table S8), crossbreed dogs (*n* = 6159) were classified as high‐grade in 16.2% of cases. In contrast, MCTs in Shar Pei (*n* = 268) were graded high‐grade in 54.1% of cases, whereas MCTs in Pugs (*n* = 2988) were high‐grade in only 2.15% of cases.

Regarding sex, when comparing ME with FE dogs, the only significant difference was within 3–6 years old where FE showed a lower odd of developing MCT compared to ME (OR: 0.8 [0.7–0.9]) (Supporting Information Table S9).

For neuter status, MN showed significantly higher odds than ME from 3 to 15 years old with ORs from 1.4 [1.2, 1.5] to 1.7 [1.4, 2.0] while FN showed greater odds than FE for all ages with ORs ranging from 1.7 [1.6, 1.9] to 2.3 [1.3, 4.2].

## 5 Melanomas

Melanomas were analysed according to the most common topographies (Table 4): oral cavity (oral melanoma), eye/adnexa (uveal melanoma), digits (cutaneous digital melanoma) and skin other than the digits (cutaneous melanoma).

**TABLE 4:** Most common melanoma topographies.

Location	Count (*n*)	Valid proportion*
Oral cavity (oral melanoma)	8183	49.4%
Skin (cutaneous melanoma)	4665	28.2%
Digits (cutaneous digital melanoma)	1715	10.4%
Eye and adnexa (uveal melanoma)	1467	8.9%

*Note*: Valid Proportion* includes only cases without unknown values.

Significant differences in age at diagnosis were found across melanoma topographies (χ^2^ = 1170, df = 3, *p* < 0.0001) and, when comparing topographies to each other, oral melanomas were diagnosed at a significantly older age (11.50 years IQR: 10.00, 13.00) than cutaneous (9.92 years IQR: 7.83, 11.58), cutaneous digital (10.00 years IQR: 8.00, 11.58) and uveal melanoma (9.42 years IQR: 7.08, 11.33). No significant difference was found between cutaneous and cutaneous digital melanoma (Supporting Information statistical analysis for melanomas in Data S2). Regarding breeds (Table 5), Rottweiler and Shar Pei ranked among the top five high‐odds breeds for three out of the four melanoma topographies. In contrast, Border Collie appeared among the five lowest‐odds breeds for three out of the four melanoma topographies.

**TABLE 5:** Top five and bottom five breed odds ratios for melanomas from the multivariable case–control model.

Melanoma location	Oral	Cutaneous	Cutaneous digital	Uveal
Top 5 breeds with the highest risk OR (95% CI)	Rottweiler 12.2 [9.5, 15.6]	Shar Pei 16.2 [10.6, 24.6]	Giant Schnauzer 50.3 [24.7, 102.5]	Dogue De Bordeaux 30.1 [14.9, 60.7]
	Shar Pei 10.3 [6.1, 17.4]	Hungarian Vizsla 15.5 [11.6, 20.8]	Rottweiler 37.5 [27.5, 51.2]	Hungarian Vizsla 18.1 [11.5, 28.7]
	Chow Chow 8.7 [3.6, 21.1]	Chow Chow 12.4 [5.8, 26.3]	Scottish Terrier 19.6 [12.5, 30.9]	Irish Terrier 17.8 [7.8, 40.3]
	Mastiff 8.7 [2.9, 25.4]	Rottweiler 10.6 [8.1, 13.7]	Bullmastiff 14.8 [6.2, 35.3]	Shar Pei 17.5 [9.1, 34.0]
	Bernese Mountain Dog 8.5 [3.9, 18.6]	Giant Schnauzer 9.5 [4.3, 20.9]	Schnauzer 12.2 [6.5, 23.00]	Spaniel (Generic) 12.8 [6.2, 26.4]
Bottom 5 breeds with the lowest risk OR (95% CI)	Lhasa Apso 0.5 [0.3, 0.8]	Jack Russell Terrier 0.3 [0.2, 0.5]	Retriever (Golden) 2.7 [1.8, 4.1]	Dachshund (Standard) 2.2 [1.0, 4.9]
	Lurcher 0.5 [0.3, 0.8]	Lhasa Apso 0.3 [0.2, 0.7]	Labradoodle 2.2 [0.9, 5.5]	Retriever (Golden) 2.1 [1.3, 3.3]
	Cairn Terrier 0.4 [0.2, 0.9]	Spaniel (English Springer) 0.3 [0.2, 0.5]	Spaniel (Cocker) 2.2 [1.6, 3.1]	Retriever (Labrador) 1.6 [1.2, 2.1]
	Greyhound 0.2 [0.1, 0.5]	Border Collie 0.2 [0.2, 0.4]	German Shepherd Dog 1.9 [1.2, 3.1]	WHWT 0.4 [0.2, 0.9]
	WHWT 0.2 [0.1, 0.3]	WHWT 0.2 [0.1, 0.4]	Border Collie 0.2 [0.1, 0.5]	Border Collie 0.2 [0.1, 0.5]

*Note*: Reference category for the breed variable: crossbreed (OR = 1). Complete multivariable logistic regression results for oral, cutaneous, cutaneous digital, and uveal melanomas are available in Supporting Information Tables S10a–d.

Abbreviation: WHWT, West Highland White Terrier.

Across all but uveal MEL topographies, and from 6 years old onwards, FE showed significantly lower odds of developing MEL compared to ME in animals ranging from 0.4 [0.2, 0.6] to 0.6 [0.5, 0.9] (Supporting Information Table S11a–d).

MN showed significant higher odds than ME in dogs older than 9 years old for oral MEL ([9–12] years: OR: 1.2 [1.1, 1.4]; 12–15 years: OR: 1.4 [1.2, 1.7]) and digital MEL (9–12 years: OR: 1.7 [1.3, 2.4]).

A similar pattern was observed when comparing FN with FE dogs although in this case, significantly greater odds were observed for uveal MEL from 6–9 (OR: 2.2 [1.2, 4.1]) to 12–15 years old (OR: 5.8 [1.8, 18.8]) (Supporting Information Table S11a–d).

## 6 Haemangiosarcomas

Haemangiosarcoma primarily affected the spleen (*n* = 5262; 47.7%), followed by the skin (*n* = 4104; 37.2%) (Supporting Information Table S12).

Splenic HSA (10.00 years IQR: 8.75, 11.17) was diagnosed at later ages than the cutaneous form (9.58 years IQR: 7.50, 11.25) (W = 4 952 618, *p*‐value < 0.001; Supporting Information statistical analysis for HSA in Data S2).

Concerning breeds (Table 6), the German Shepherd Dog had the highest odds for splenic HSA while Mastiff and Bullmastiff had the top two ORs for cutaneous HSA. Conversely, the Yorkshire Terrier, West Highland White Terrier, and Border Terrier were among the five breeds with the lowest odds for HSA.

**TABLE 6:** Top five and bottom five breed odds ratios for haemangiosarcomas from the multivariable case–control model.

Haemangiosarcoma location	Splenic	Cutaneous
Top 5 breeds with the highest risk OR (95% CI)	German Shepherd Dog 7 [5.9, 8.3]	Mastiff 20.8 [10.6, 40.9]
	Retriever (Flat Coated) 6.2 [4.2, 9.3]	Bullmastiff 14.2 [8.4, 23.9]
	Hungarian Vizsla 5.6 [3.7, 8.5]	Dogue De Bordeaux 13.3 [7.4, 24.0]
	Italian Spinone 5.5 [2.4, 12.4]	Bulldog 5.9 [3.9, 9.1]
	Belgian Shepherd Dog 5.1 [2.0, 13.3]	Boxer 5.4 [4.2, 6.9]
Bottom 5 breeds with the lowest risk OR (95% CI)	Jack Russell Terrier 0.3 [0.2, 0.5]	Spaniel (English Springer) 0.5 [0.3, 0.7]
	Spaniel (English Springer) 0.3 [0.2, 0.5]	Terrier (Generic) 0.4 [0.2, 0.9]
	WHWT 0.3 [0.2, 0.5]	Border Terrier 0.3 [0.2, 0.5]
	Yorkshire Terrier 0.3 [0.1, 0.5]	CKCS 0.2 [0.1, 0.5]
	Border Terrier 0.2 [0.1, 0.4]	Yorkshire Terrier 0.2 [0.1, 0.4]

*Note*: Reference category for the breed variable: crossbreed (OR = 1). Complete multivariable logistic regression results for splenic and cutaneous haemangiosarcomas are available in Supporting Information Table S13a,b.

Abbreviations: CKCS, Cavalier King Charles Spaniel; WHWT, West Highland White Terrier.

In relation to sex, FE showed significantly lower odds than ME for cutaneous HSA on ranges of age 3–6 (OR: 0.5 [0.3, 0.8]) years and 9–12 years (OR: 0.4 [0.3, 0.6]) as well as significantly lower odds for splenic HSA from 6–9 years (OR: 0.5 [0.3, 0.7]) to 9–12 years (OR: 0.7 [0.5, 0.9]) (Supporting Information Table S14a,b).

MN held greater odds for both cutaneous and splenic HSA than ME in dogs 6–15 years old with ORs ranging from 1.5 [1.2, 1.8] to 2.1 [1.5, 3.1], similarly to comparisons between FN and FE where ORs ranged from 1.7 [1.2, 2.5] to 2.5 [1.8, 3.5] (Supporting Information Table S14a,b).

## 7 Osteosarcomas

Overall, OSA were mostly located in the long bones (hindlimbs [*n* = 648] and forelimbs [*n* = 593]; 26.6%), oral cavity (*n* = 1091; 23.4%), and the mammary gland (*n* = 431; 9.3%) (Supporting Information Table S15) with a different location distribution between high‐odds and low‐odds breeds (χ^2^ = 237.32, df = 35, *p* < 0.0001) (Supporting Information statistical analysis for OSA). Specifically, OSA most commonly affected long bones in high‐odds breeds, whereas mammary gland and oral cavity tumours were more frequent in low‐odds breeds (Supporting Information Tables S16 and S17).

For age, again significant differences were observed between breeds. Dogs from breeds at high‐odds of OSA were diagnosed at a younger median age (8.08 years IQR: 6.83, 10.00) compared to those from low‐odds breeds (10.00 years IQR: 8.50, 12.00) (W = 42 254, *p*‐value < 0.0001) (Supporting Information statistical analysis for OSA in Data S2).

Large breeds, such as Rottweiler (OR: 10.6 [8.4, 13.3]), and Bullmastiff (OR: 9.6 [5.6, 16.6]), as well as sighthounds like the Deerhound (OR: 8.2 [3.4, 20.1]), were among the top 10 high‐odd breeds for OSA while lowest‐odd breeds were typically smaller, including Lhasa Apso (OR: 0.2 [0.1, 0.6]) and Cavalier King Charles Spaniel (OR: 0.2 [0.1, 0.4]) (Table 7).

**TABLE 7:** Top 10 and bottom 10 breed odds ratios for osteosarcoma from the multivariable case–control model.

Top 10 breeds with the highest OR (95% CI)	Bottom 10 breeds with the lowest OR (95% CI)
Leonberger: 17.2 [8.6, 34.2]	Spaniel (Cocker): 0.5 [0.4, 0.7]
Mastiff: 13.6 [6.7, 27.4]	Yorkshire Terrier: 0.5 [0.3, 0.7]
Rottweiler: 10.6 [8.4, 13.3]	Bichon Frise: 0.4 [0.2, 0.8]
Bullmastiff: 9.6 [5.6, 16.6]	Border Collie: 0.4 [0.3, 0.6]
Deerhound: 8.2 [3.4, 20.1]	Jack Russell Terrier: 0.4 [0.3, 0.5]
St. Bernard: 6.6 [2.5, 17.1]	West Highland White Terrier: 0.4 [0.3, 0.6]
Dobermann: 6.3 [4.3, 9.4]	Border Terrier: 0.3 [0.2, 0.6]
Italian Spinone: 5.4 [2.5, 11.6]	Spaniel (English Springer): 0.3 [0.2, 0.4]
Old English Sheepdog: 5 [2.4, 10.1]	Cavalier King Charles Spaniel: 0.2 [0.1, 0.4]
Rhodesian Ridgeback: 4.6 [2.8, 7.4]	Lhasa Apso: 0.2 [0.1, 0.6]

*Note*: Reference category for the breed variable: crossbreed (OR = 1). Complete multivariable logistic regression results are available in Supporting Information Table S18.

Regarding sex, a significant difference between entire dogs of both sexes was found only within the range of age 12–15 (OR: 1.8 [1.2, 2.9]) (Supporting Information Table S19). Neutered dogs from both sexes showed significantly greater odds for OSA than their entire counterparts. In males, the age range was from 6 to 15 years old, while in females, it was only from 6 to 12 years old (Supporting Information Table S19).

## 8 The Role of Hormonal Alterations in Tumour Development

As detailed above for each tumour type, the effect of being neutered was evaluated separately in females and males across age groups (e.g., in MCT, MN consistently showed higher odds than ME from 3 to 15 years, while FN showed higher odds than FE across all age ranges).

In a final step, we directly compared the magnitude of this neuter–status association between sexes for each tumour–age combination by contrasting the OR for FN versus FE with the corresponding OR for MN versus ME within the same age group for instance, comparing the FN–FE OR for MCT in the (6–9) range with the MN–ME OR for that same age range.

**FIGURE 1:**
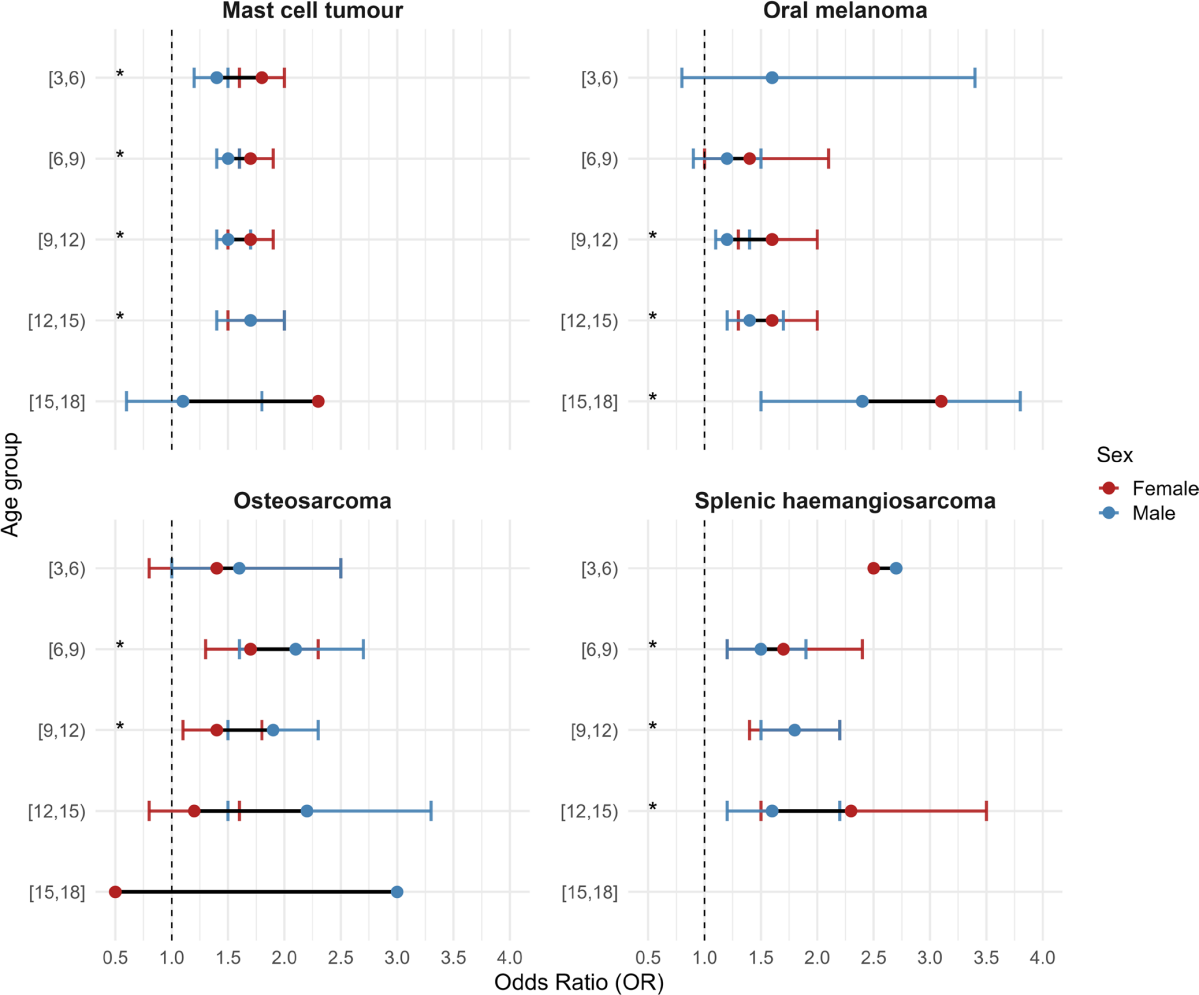
Sex‐specific associations between neuter status and tumour diagnosis for four representative tumour types and their age groups. Red and blue points represent odds ratios (OR) for being neutered versus entire in females (FN vs. FE) and males (MN vs. ME), respectively, for each tumour–age combination, with horizontal lines indicating their 95% confidence intervals. The black segment joins the female and male estimates within each age group to illustrate the magnitude and direction of the sex‐specific difference. An asterisk next to an age band denotes tumour–age combinations in which the neutered‐versus‐entire contrast was statistically significant in both sexes. The vertical dashed line marks the null value (OR = 1). Full estimated marginal means and pairwise comparisons for all sex–age combinations are presented in the Supporting Information emmeans output Tables (S9, S11a–d, S14a,b, and S19), together with tumour‐specific plots displayed in Supporting Information Figures S10–S17.

Figure 1 displays these comparisons for four illustrative tumour types (MCT, oral melanoma, splenic haemangiosarcoma and osteosarcoma). For each tumour, red and blue points represent the ORs for female (FN vs. FE) and male (MN vs. ME) comparisons, respectively, with their 95% confidence intervals. The black segment linking both points at each age band reflects the magnitude and direction of the sex‐specific difference in the neuter–status association. An asterisk next to an age band indicates that the neutered‐versus‐entire contrast was statistically significant in both sexes for that tumour–age combination. Full estimated marginal means and pairwise comparisons for all tumour types and age groups are provided in the Supporting Information emmeans output Tables (S9, S11a–d, S14a,b, and S19), together with individual plots for each tumour (Supporting Information Figures S10–S17).

In total, 18 tumour–age combinations were significantly and positively associated with tumour diagnosis in both sexes (12 of these combinations are shown with an asterisk in Figure 1), while the other 6 appear in the tumour‐specific figures: Supporting Information Figure S12 Cutaneous melanoma (2 asterisks), Supporting Information Figure S13 Cutaneous digital melanoma (1 asterisk)‐ and Supporting Information Figure S16 Cutaneous haemangiosarcoma (3 asterisks).

Among them, females consistently exhibited higher ORs than males in 12 cases, with the exceptions of MCT in the (12–15) age group, cutaneous HSA in the (6–9) and (12–15) age groups, splenic HSA in the (9–12) age group, and OSA in the (6–9) and (9–12) age groups. Consistent with this pattern, the mean OR for females was 1.87 (SD ± 0.41), compared with 1.66 (SD ± 0.31) in males.

## 9 Discussion

In this study we present the largest veterinary PTR to date, covering over a million tumours diagnosed in dogs across the UK. Given the wealth of data available we opted to focus on four major tumour types: MCT, MEL, HSA and OSA, and shed light upon important trends in their frequency, breed predispositions, topographical distribution, and associations with age, sex, and neuter status.

Our findings on breed‐specific cancer odds are broadly consistent with previous literature, reinforcing known associations such as dark‐coated dogs being more prone to MEL, molossoid breeds to OSA, bulldog‐related breeds to MCT, and German Shepherds to HSA; thus, confirming similar genetically‐associated risk in UK breeds as in the same breeds in other countries.

Moreover, the large scale of our registry enabled the identification of new, often underrepresented, breed associations such as the Irish Terrier for MCT, Newfoundland and British terriers (such as the Norfolk and Welsh Terriers) for different MEL topographies, and the Italian Spinone for both HSA and OSA.

The analysis also revealed significant effects of neuter status, age, and sex on tumour odds, with notable variation across tumour types. Notably, in comparison with other studies in the field [34, 35], that evaluated age and neuter status as additional covariates in multivariable models, our large sample size enabled a more refined assessment of neuter status by directly comparing neutered and entire dogs within the same age groups, thereby substantially reducing age‐related confounding.

Mast cell tumour was the most frequent tumour type in our study. MCTs were mostly located on the skin (90%) [23, 24, 25, 27, 36, 37, 38, 39]. The vast majority of cases for which grade was annotated were Grade 2 and low‐grade primarily affecting middle‐to elderly animals, consistent with previous work that found less common and more aggressive MCTs affecting older dogs [25, 40]. Similarly, in keeping with previous literature, we identified Bulldog‐derived breeds such as Boston Terrier, Bullmastiff, French Bulldog or Boxer as well as Retrievers (Curly and Flat Coat, Golden and Labrador), Shar Pei and Weimaraner as being predisposed to MCT [24, 25, 36, 37, 38].

The finding of increased risk in Bulldog‐derived breeds, such as the Boxer and Bullmastiff, may reflect a shared ancestral configuration associated with excessive risk for MCTs. This idea was first proposed by Peters in 1969 [41] who demonstrated that the high prevalence of MCTs in Boxers, Boston Terriers, and Bullterriers could be traced back to common lineage involving the Bulldog and the (now‐extinct) English Terrier. More recently, genomic analysis led to the definition of the European Mastiff clade, which also includes Boxer, Bull Terrier and Boston Terrier, along with French Bulldog, Bullmastiff and Bulldog, that in our study showed consistently elevated odds for MCT in line with former epidemiological literature on the field [20, 24, 25, 36, 37, 38].

Our study found associations between breed and MCT grade, thus suggesting a genetic link underlying not only tumour development but also tumour behaviour and aligning with previous studies [40, 42, 43]. For example, MCTs affecting Pug and Boston Terrier were more commonly lower grade, whereas those on Shar Pei are more frequently higher grade. Understanding these subtly different disease phenotypes can provide useful opportunities for a more tailored approach for canine health screening and potential breed‐specific prevention strategies, ultimately improving the outcomes for at‐risk breeds. Former studies [20, 24, 44] have failed to find a sex predisposition for MCT. In our case, we only found significantly lower odds for FE compared with ME within the range of 3 to 6 years old, while across other age groups, the differences were not significant. In this sense, given the absence of a consistent pattern across age groups and the lack of supporting evidence in the literature, we acknowledge that this result should be interpreted with caution given that it may reflect a statistical fluctuation or an unmeasured confounding factor.

Canine melanomas closely resemble non‐UV‐induced human melanomas in several aspects.

Regarding tumours located on the distal extremities, canine digital melanoma may serve as a translational model for human acral lentiginous melanoma [13]. However, important anatomical differences exist: human acral melanomas typically arise on the palmar, plantar, or subungual regions [45], while canine digital melanomas are more restricted to the digits, footpad, or nail bed [13]. To reflect this anatomical specificity, in this discussion we will refer to the human condition as cutaneous acral melanoma and to its canine counterpart as digital melanoma.

Specifically, canine oral and cutaneous digital melanomas exhibit aggressive behaviour similar to that of human mucosal and cutaneous acral melanomas [13]. In humans, these tumours are more common in certain ethnic groups, suggesting a genetic component of the risk [46, 47]. We identified several dog breeds, especially dark‐coated breeds, at increased risk of non‐UV‐induced melanomas, supporting previous findings of their potential as models of genetic risk factors for these tumours [13].

Oral and cutaneous acral melanomas are rare in humans [46, 48, 49]. In contrast, half of the canine melanomas reported here were located in the oral cavity, and 10% were located on the digital region, consistent with previous studies [13, 50, 51]. However, similarities arise in uveal melanomas, which are the most common malignant ocular primary tumours in both humans [52] and dogs, as evidenced by our study and others [53].

Women appear to be more frequently affected by mucosal melanomas, likely due to the higher incidence of urogenital forms, which are more common in females [53] while men have a higher risk of uveal melanoma [54]. When it comes to cutaneous acral melanoma, while survival outcomes are poorer in men compared to women [45], the incidence is similar between sexes [55].

In our study, cutaneous, cutaneous digital and oral melanomas showed significantly lower odds in entire females compared to entire males in line with previous studies [54] with no significant difference observed for uveal melanoma. Concerning age, human mucosal (oral) and cutaneous acral melanomas are diagnosed at a median age of greater than 65 years [45, 56], followed by cutaneous at 65 years [49] and uveal at 62 [57] years. Similarly, in dogs, we also found oral melanomas were diagnosed at significantly older ages in comparison with other analysed topographies [54] although unlike in humans, where cutaneous acral melanomas tend to appear later than UV‐induced cutaneous melanomas, no significant age difference was observed between cutaneous and cutaneous digital melanomas in dogs reflecting potential species‐specific differences in tumour development or variations in exposure to environmental risk factors.

Melanomas appear to be influenced by pigmentation in both humans and dogs. In humans, cutaneous and uveal melanomas are more prevalent among fair‐skinned and light‐eyed individuals [49, 57, 58], whereas cutaneous acral melanomas tend to occur more frequently in darker‐skinned populations [49]. Finally, some studies suggest that ethnicity may be a risk factor for the anatomical distribution of mucosal melanomas, with anorectal tract melanomas being more common in Asian populations, genitourinary melanomas in Caucasians, and head and neck mucosal melanomas in non‐Hispanic White populations [59].

Conversely, in dogs, pigmentation appears to be associated with increased odds for melanoma across all topographies, including both cutaneous and non‐cutaneous. Our analysis consistently identified breeds with darker or more pigmented coats, such as Rottweiler, Scottish Terrier, Bernese Mountain Dog, Schnauzer, Hungarian Vizsla, Flat Coated Retriever among others as having higher odds of developing melanomas across multiple anatomical sites. Conversely, lighter‐coated breeds, such as the West Highland White Terrier and Lhasa Apso, showed a lower association with both cutaneous and non‐cutaneous melanomas, supporting a potential role of pigmentation in melanoma susceptibility. This difference may reflect the balance of the solar protection offered by the hair coat versus the total number of melanocytes on a dog (and thus the number of cells in which a malignant transformation could occur).

Canine haemangiosarcoma is pathologically similar to angiosarcoma in humans and shows similar biological behaviours, including rapid growth, high invasiveness, and a tendency to metastasize [17].

In a study of human angiosarcoma, 72.3% of cases were cutaneous, subcutaneous, or breast‐associated, and 60.8% occurred in women. Among the remaining cases, 24.4% were visceral, most commonly involving the liver (27.6%) and heart (13.4%) [16]. Within the SAVSNET PTR, approximately three‐quarters of visceral HSA cases affected the spleen, which is consistent with previous veterinary literature [60]. Notably, in contrast to previous studies that reported no sex predilection [61, 62], we observed a higher odds in male dogs for both splenic and cutaneous HSA.

In humans, the median age at diagnosis of all skin, subcutaneous, and breast angiosarcomas is 72 [62–80] years, while those presenting with visceral angiosarcoma are, on average, younger (65 [52–75] years) [16]. However, according to our data, splenic HSA were diagnosed at older ages than cutaneous HSA consistent with cases from the Golden Retriever Lifetime Study (GRLS) where visceral HSA was diagnosed at 8.7 years old and cutaneous HSA at 8.1 years old [61].

Previous studies have identified common breeds, such as German Shepherd Dogs and Golden Retrievers, having high odds for splenic HSA [61, 63]. In our study, although German Shepherd dogs displayed the highest OR for splenic HSA, several less frequent breeds like the Flat Coated‐Retriever, Belgian Shepherd Dog and Hungarian Vizsla obtained higher odds than Golden Retrievers.

For cutaneous HSA, the literature distinguishes between an actinic form, which is related to chronic sun exposure, and a non‐actinic form, for which the aetiology is less clear [63].

Actinic HSA tends to occur in breeds predisposed by their lightly pigmented and glabrous skin, such as Whippets, Greyhounds, Beagles, Pit Bulls, and Dalmatians, where the higher prevalence of cutaneous HSA is linked to their reduced protection against solar radiation. These findings are consistent with recent human data showing that ambient UV radiation is associated with increased incidence of cutaneous angiosarcoma, particularly in sun‐exposed regions such as the head and neck and among light‐skinned individuals [64]. This further supports the biological plausibility of UV‐driven tumorigenesis in both humans and dogs, and underscores the relevance of actinic HSA in certain canine breeds as a spontaneous model for studying UV‐induced vascular cancers.

In contrast, non‐actinic cutaneous HSA occurs more frequently in breeds with darker, pigmented skin and thicker coats with typically no history of sun exposure and not displaying histopathological features indicative of sun‐related damage [63].

In this sense, we also identified lightly pigmented and glabrous skin breeds such as Beagle, Greyhound, Whippet or English Pointer to be at greater odds of cutaneous HSA. However, within this group, we also identified a cluster of molossoid breeds, including the Bullmastiff, Mastiff, Boxer, and Bulldog included in the European Mastiff clade [65], a genetically defined group that shares a common ancestral background and morphological features, and which showed markedly elevated odds for cutaneous HSA despite not fitting the actinic phenotype but rather being breeds with typically darker pigmentation and thicker skin, suggesting a potential non‐actinic, genetically driven subtype.

Some studies have pointed to potential ethnic differences in the incidence of certain soft tissue sarcomas in humans, including malignant vascular tumours; the role of other potential important confounding factors such as socioeconomic and environmental influences remains to be determined [66].

Osteosarcomas primarily affect the long bones of both humans and dogs, typically occurring in younger individuals. In both species, these tumours are highly metastatic and often spread to the lungs [19, 67].

A recent study analysing 5000 cases of human OSA found that the majority of cases were reported in individuals 10–24 years old, with a secondary peak (only in males) in individuals older than 80 years [19]. For all ages, most cases occurred in the lower long bones, although the axial locations became more frequent with age. Among all ages, males were slightly more at risk than females. In addition, two studies found Black/Non‐hispanic Black Americans showed an increased incidence rate suggesting a possible genetic risk [19, 66].

In dogs, known differences in breed risk have been used to support the underlying genetic risk. We found that molosoids (such as Bullmastiff and Rottweiler) and Sighthound (like Greyhound) were at a higher odds of developing OSA of the appendicular skeleton and at younger ages, reinforcing the previous findings linking large breed with increased OSA risk; [18, 34, 67, 68] in contrast, small breeds, where OSAs were more commonly found in the oral cavity or the mammary glands of female dogs, tended to be diagnosed at older ages. In contrast to humans, we found no sex predilection for OSA, which is consistent with the results of previous studies [34, 67].

Mechanistic similarities in OSA between dogs and humans have been proposed [69] and the tyrosine kinase *c‐Met* receptor has also been suggested as an important oncogenic factor in human OSA [70]. Interestingly, in dogs, the Rottweiler breed, identified as having a higher risk of OSA in this and other studies [34, 67], has been found to have a high incidence of *c‐Met* germline mutations [71], suggesting an avenue for further comparative studies.

Finally, when it comes to analysing the role of neutering as a risk factor for developing the tumours under study, we explored both the differences between neutered and entire dogs as well as the impact of neutering by sex adjusted by age. In general, entire (non‐neutered) dogs (of both sexes) consistently exhibited lower odds ratios (ORs) for developing tumours compared to neutered dogs.

More in detail, when analysing the difference between ORs from comparison between entire and neutered dogs from both sexes (Female neutered vs. Female entire and Male neutered vs. Male entire), neutering had a consistently greater impact in females than in males across most tumour types. This pattern was observed in MCTs, all but uveal MEL topographies (oral, cutaneous, cutaneous digital) and HSAs (splenic and cutaneous), where the odds ratios for FN versus FE were consistently higher than those observed for MN versus ME.

Overall, these findings are in line with several epidemiological studies that have reported similar results regarding neuter status and various types of cancer. For instance, the Swiss Canine Cancer Registry (SCCR) [68] reported higher ORs for both neutered male and female dogs with MCT. Conversely, While et al. identified neutering as a risk factor only in female dogs [72], Shoop et al. found lower odds of MCT in both male and female neutered dogs but noted limitations in data accuracy for this variable [20].

For bone tumours in the SCCR, only neutered males showed higher ORs [68], whereas two UK studies on OSA found that both neutered females and males had higher ORs than all dogs [34, 67]. Additionally, the SCCR [68] reported higher ORs only for neutered females with vascular and melanocytic tumours, and finally, a 1999 study focusing exclusively on cardiac tumours [73], found that neutered females and males had a relative risk more than 5 and 2.4 times greater, respectively, than their entire counterparts.

The basis for these repeatable findings is currently uncertain. Castration and ovariectomy are common practices used in canine population management. In humans, similar surgical procedures are sometimes performed for health reasons (although typically in older populations). Recent European and American studies have found that women over 45 years of age who underwent bilateral salpingo‐oophorectomy (BSO) had an increased risk of developing chronic medical conditions, including cancer [74, 75]. Biological mechanisms underlying the higher risk of cancer in women who have undergone BSO or in neutered dogs (especially female dogs) are not entirely clear, but one possible explanation could be related to the chronic elevation of luteinizing hormone (LH) that occurs after gonadal removal (or similar conditions such as menopause or testicular cancer) in both male and female individuals of both species, resulting from the loss of negative feedback to the hypothalamus and anterior pituitary following the removal of the gonads [76, 77, 78, 79] and given the ubiquity of LH receptors in different organs [77, 80, 81, 82, 83].

However, it is important to acknowledge that we do not know the age at neutering in the animals included in this study, and consequently, the duration of exposure to hormonal effects remains unknown. A clinical records‐based approach would be required to accurately assess the timing of neutering and its long‐term impact.

In the canine population, socioeconomic factors may also play a role, as pet dog owners who have their dogs spayed or neutered are often more likely to pursue tumour biopsies [84]. Large datasets such as those presented herein offer new and unparalleled opportunities to explore the wider impact of neutering on cancer risk.

## 10 Strengths and Limitations of This Study

The primary strength of the SAVSNET PTR lies in its scale and ability to deliver near real‐time analysis of tumour trends. The extensive dataset enabled the identification of breeds not previously recognised as being at altered risk and offers the potential to investigate less frequent or poorly characterised tumours that were not included in the present analysis, demonstrating a key advantage of a large‐scale registry.

While this paper presents the current state of the PTR, its nature as a digital tool means it is continually evolving, with the capacity to integrate new data sources and adapt to emerging research priorities. For instance, data on environmental exposures (e.g., air pollution, pesticide use, UV radiation), biological conditions (e.g., obesity, antibiotic resistance, infectious disease history), and even owner‐linked variables—such as socioeconomic status, smoking habits, obesity, or personal cancer diagnoses—could be integrated. These additions would allow for a more holistic perspective on cancer risk in companion animals.

Therefore, while the scope of this work is limited to veterinary epidemiology, the insights generated may support future comparative oncology research in such a way that dogs can be viewed as sentinels of shared environmental and health risks, providing valuable insight not only into veterinary oncology but also into factors relevant to human health. The PTR is well aligned with comparative oncology principles, which form a core component of the One Medicine framework. It is a resource to bridge veterinary and human cancer research by identifying breed‐associated cancer risks with potential analogues in human populations, offers large‐scale epidemiological data to support studies of shared genetic and environmental risk factors, and provides a foundation for collaborative research, particularly in tumour types where canine models have translational relevance, such as the ones covered in this paper.

The limitations of this study include the possibility that more easily biopsied (i.e., external) or clinically concerning tumours are more likely to be submitted for analysis, potentially introducing selection bias. Tumour diagnosis and reporting are not mandated in veterinary populations and, as such, the lack of completeness in the PTR must be taken into account, as the findings can only represent tumours that were submitted for histopathology. Socioeconomic factors may also influence both dog breed ownership and access to diagnostic services, thereby affecting which animals are represented in the pathology data [84]. In addition, a UK study on dogs with suspected multicentric lymphoma found that owners of entire dogs were less likely to pursue tissue confirmation, particularly in more socioeconomically deprived households. Although lymphoma was not included in our analysis, a similar pattern could plausibly affect other tumour types, leading to under‐representation of entire dogs in pathology submissions and thereby potentially inflating the apparent association between neuter status and tumour diagnosis [85].

Another constraint lies in the absence of a true, up‐to‐date reference population of dogs, which limits our ability to directly estimate population‐level risk. As a result, we relied on secondary demographic sources, such as veterinary consultation records, which may not fully reflect the actual breed distribution in the general population (as not all animals are presented to a veterinary surgery).

A further consideration is that, while veterinary professionals are highly experienced in identifying dog breeds, the secondary nature of the data prevents confirmatory checking, although systematic bias in breed identification is unlikely.

The structure of our tumour registry means that submission of material from one case by more than one practice (e.g., two branches of the same group) could not be detected and therefore the dataset is likely to include a small number of duplicate cases. Similarly primary and metastatic lesions submitted in separate submission events would not be detected as linked.

These limitations are further compounded by constraints inherent to the study design. For example, despite the well‐documented predisposition of Boxer, French Bulldog, and Pug to MCTs, these breeds were excluded from the primary logistic regression analysis due to an insufficient number of eligible controls.

Limitations related to the rules‐based text‐mining (TM) approach used for diagnosis extraction must also be considered. Although previous validation [10] showed TM achieved an average accuracy of 95% in extracting diagnosis from EPR, demonstrating its reliability in structured datasets, they are not without error due to their lack of contextual understanding [86]. This limitation is particularly relevant when dealing with complex, context‐dependent free‐text medical narratives, such as those found in pathology reports that commonly include differential diagnosis, staging details, ambiguous language, or non‐standard terminology, which can challenge deterministic rule‐based systems. For instance, while lymphomas typically originate in lymphoid tissues, other tumour types recorded at lymph node locations may, in fact, represent metastases rather than primary neoplasms. This may be most relevant for MCTs in the present study. Additionally, in order to minimise false positives, we excluded records containing explicitly non‐diagnostic terms selected based on expert criteria and previous literature [87]. In this sense, this study also lays the groundwork for the development of AI‐based models that can interpret these narratives more effectively, potentially improving future risk assessments in veterinary oncology.

## 11 Conclusion

- Our findings in relation to breed associations with certain canine tumours confirm previously recognised breed predispositions, including Bulldog‐related breeds for MCT, dark‐coated breeds for MEL, German Shepherd Dogs for HSA, and molossoid breeds for OSA while also expanding upon them by identifying additional breeds at increased or reduced risk within these tumour types.
- Neutering is positively associated with tumour occurrence across the major tumour types studied and this association is partly independent of age, suggesting potential additional biological or hormonal mechanisms.
- Together, these findings demonstrate the power of large‐scale, pathology‐based registries to generate robust, real‐time epidemiological evidence in veterinary oncology. Such registries make it possible to reveal associations between specific tumours and dogs from less common or geographically restricted breeds that would otherwise remain undetected in smaller datasets. By the same principle, less common or poorly characterised tumour types can also emerge within these comprehensive datasets, expanding opportunities for discovery and advancing our understanding of canine cancer.
- The PTR could also serve as a valuable tool to identify cases of interest, which can then be followed up for targeted molecular investigation. In previous work, we described the development of a ‘virtual biobank’, which enables us to locate and annotate such cases and request archived tissue samples from participating laboratories for genotyping and further analysis [88].
- In the broader context of One Health and Comparative Oncology, this registry provides a valuable bridge between veterinary and human cancer research. By linking naturally occurring tumours in dogs with comparable human cancers, it supports the identification of shared genetic and environmental risk factors and fosters collaborative research. Future efforts to enhance data capture, longitudinal follow‐up, and integration with molecular datasets will further strengthen its role as a foundational tool for cross‐species cancer surveillance and translational research.
- Future efforts to expand data capture, integrate longitudinal follow‐up, and align veterinary data with human cancer research will be critical for realising the full value of this resource in comparative oncology and cross‐species cancer surveillance.

## Funding

This work was supported by Pet Plan Charitable Trust (S22‐1079‐1118). We are also grateful for the support from the veterinary diagnostic laboratories that routinely submit data to SAVSNET.

## Ethics Statement

Data collection and use by SAVSNET is ethically approved by the University of Liverpool Research Ethics Committee (RETH001081).

## Conflicts of Interest

Francesco Cian is a clinical pathologist at Batt Laboratories. Annika Herrmann is an Anatomic Pathologist for the Veterinary Pathology Group (VPG). Jenny S. McKay is the Head of Anatomic Pathology at Idexx Laboratories. Matthew Jones is the Global Technical Platform Manager at Idexx Laboratories. Other authors declare that they have no conflicts of interest.

## Supporting information


**Data S1:** Supporting Information.

**Data S2:** Supporting Information.

## Data Availability

The histopathology reports on which the entire final published dataset is based cannot be made available in an open access format, as they sometimes contain clinically and financially sensitive information relating to the diagnostic laboratory or veterinary practice, as well as rare potentially identifying information. However, access to summary data or specific cohorts may be possible by reasonable request for use, in line with University of Liverpool. Researchers wishing to access the raw data must apply for access here: https://www.liverpool.ac.uk/savsnet/using‐savsnet‐data‐for‐research/ where assessment will be made based on objectives, publication strategy, and track record.

## References

[1] J. D. Schiffman and M. Breen , “Comparative Oncology: What Dogs and Other Species Can Teach Us About Humans With Cancer,” Philosophical Transactions of the Royal Society, B: Biological Sciences 370, no. 1673 (2015): 20140231. 10.1098/rstb.2014.0231 PMC4581033 26056372

[2] National Academies of Sciences E , S. J. Nass , C. Berkower , R. Cooper , Forum on Aging D , and National Cancer Policy Forum (U.S.) , “Companion Animals as Sentinels for Predicting Environmental Exposure Effects on Aging and Cancer Susceptibility in Humans Proceedings of a Workshop,” in Companion Animals as Sentinels for Predicting Environmental Exposure Effects on Aging and Cancer Susceptibility in Humans Proceedings of a Workshop (National Academies Press, 2022). 35593768

[3] J. H. Oh and J. Y. Cho , “Comparative Oncology: Overcoming Human Cancer Through Companion Animal Studies,” Experimental & Molecular Medicine 55, no. 4 (2023): 725–734. 37009802 10.1038/s12276-023-00977-3 PMC10167357

[4] E. P. Cherniack and A. R. Cherniack , “Assessing the Benefits and Risks of Owning a Pet,” Canadian Medical Association Journal 187, no. 10 (2015): 715–716. 26078462 10.1503/cmaj.150274 PMC4500685

[5] M. Roccaro , R. Salini , M. Pietra , et al., “Factors Related to Longevity and Mortality of Dogs in Italy,” Preventive Veterinary Medicine 225 (2024): 106155. 38394961 10.1016/j.prevetmed.2024.106155

[6] D. G. O'Neill , D. B. Church , P. D. McGreevy , P. C. Thomson , and D. C. Brodbelt , “Longevity and Mortality of Owned Dogs in England,” Veterinary Journal 198, no. 3 (2013): 638–643. 24206631 10.1016/j.tvjl.2013.09.020

[7] S. R. Urfer , M. Kaeberlein , D. E. L. Promislow , and K. E. Creevy , “Lifespan of Companion Dogs Seen in Three Independent Primary Care Veterinary Clinics in the United States,” Canine Medicine and Genetics 7, no. 1 (2020): 7. 32835231 10.1186/s40575-020-00086-8 PMC7386164

[8] Grand View Research , “Veterinary Oncology Market Size, Share & Trends Analysis Report By Therapy (Radiotherapy, Surgery), By Animal Type (Canine, Feline), By Cancer Type, By Region, And Segment Forecasts, 2024–2030,” 2022, accessed September 23, 2024, https://www.grandviewresearch.com/industry‐analysis/veterinary‐oncology‐market.

[9] K. C. Pinello , F. L. P. G. Queiroga , A. de Matos , et al., “The Global Initiative for Veterinary Cancer Surveillance (GIVCS): Report of the First Meeting and Future Perspectives,” Veterinary and Comparative Oncology 18, no. 2 (2020): 141–142. 32048793 10.1111/vco.12577

[10] J. Rodríguez , D. R. Killick , L. Ressel , et al., “A Text‐Mining Based Analysis of 100,000 Tumours Affecting Dogs and Cats in the United Kingdom,” Scientific Data 8, no. 1 (2021): 266. 34654839 10.1038/s41597-021-01039-x PMC8519962

[11] D. A. Singleton , J. McGarry , J. R. Torres , et al., “Small Animal Disease Surveillance 2019: Pruritus, Pharmacosurveillance, Skin Tumours and Flea Infestations,” Veterinary Record 185, no. 15 (2019): 470–475. 31628231 10.1136/vr.l6074

[12] E. S. Dhein , U. Heikkilä , A. Oevermann , et al., “Incidence Rates of the Most Common Canine Tumors Based on Data From the Swiss Canine Cancer Registry (2008 to 2020),” PLoS One 19, no. 4 (2024): e0302231. 38635572 10.1371/journal.pone.0302231 PMC11025767

[13] A. Prouteau and C. André , “Canine Melanomas as Models for Human Melanomas: Clinical, Histological, and Genetic Comparison,” Genes 10, no. 7 (2019): 501. 31262050 10.3390/genes10070501 PMC6678806

[14] K. L. B. Blacklock , K. Donnelly , Y. Lu , et al., “Oronasal Mucosal Melanoma Is Defined by Two Transcriptional Subtypes in Humans and Dogs With Implications for Diagnosis and Therapy,” Journal of Pathology 265, no. 3 (2025): 245–259. 39828982 10.1002/path.6377 PMC11794980

[15] M. A. Griffin , W. T. N. Culp , and R. B. Rebhun , “Canine and Feline Haemangiosarcoma,” Veterinary Record 189, no. 9 (2021): e585. 34213807 10.1002/vetr.585

[16] M. J. Wagner , V. Ravi , S. K. Schaub , et al., “Incidence and Presenting Characteristics of Angiosarcoma in the US, 2001‐2020,” JAMA Network Open 7, no. 4 (2024): E246235. 38607625 10.1001/jamanetworkopen.2024.6235 PMC11015348

[17] K. Heishima , N. Aketa , M. Heishima , and A. Kawachi , “Hemangiosarcoma in Dogs as a Potential Non‐Rodent Animal Model for Drug Discovery Research of Angiosarcoma in Humans,” Frontiers in Oncology 13 (2023): 1250766. 38130992 10.3389/fonc.2023.1250766 PMC10733437

[18] K. M. Makielski , L. J. Mills , A. L. Sarver , et al., “Risk Factors for Development of Canine and Human Osteosarcoma: A Comparative Review,” Veterinary Sciences 6, no. 2 (2019): 48. 31130627 10.3390/vetsci6020048 PMC6631450

[19] S. Cole , D. M. Gianferante , B. Zhu , and L. Mirabello , “Osteosarcoma: A Surveillance, Epidemiology, and End Results Program‐Based Analysis From 1975 to 2017,” Cancer 128, no. 11 (2022): 2107–2118. 35226758 10.1002/cncr.34163 PMC11647566

[20] S. J. Shoop , S. Marlow , D. B. Church , et al., “Prevalence and Risk Factors for Mast Cell Tumours in Dogs in England,” Canine Genetics and Epidemiology 2, no. 1 (2015): 1. 26401329 10.1186/2052-6687-2-1 PMC4579370

[21] H. Aupperle‐Lellbach , J. M. Grassinger , A. Floren , et al., “Tumour Incidence in Dogs in Germany: A Retrospective Analysis of 109,616 Histopathological Diagnoses (2014–2019),” Journal of Comparative Pathology 198 (2022): 33–55. 36116890 10.1016/j.jcpa.2022.07.009

[22] E. Baioni , E. Scanziani , M. C. Vincenti , et al., “Estimating Canine Cancer Incidence: Findings From a Population‐Based Tumour Registry in Northwestern Italy,” BMC Veterinary Research 13, no. 1 (2017): 203. 28659149 10.1186/s12917-017-1126-0 PMC5490209

[23] L. B. Brønden , T. Eriksen , and A. T. Kristensen , “Mast Cell Tumours and Other Skin Neoplasia in Danish Dogs‐Data from the Danish Veterinary Cancer Registry,” 2010, http://www.actavetscand.com/content/52/1/6. 10.1186/1751-0147-52-6 PMC2823750 20096110

[24] A. L. Martins , A. Canadas‐Sousa , J. R. Mesquita , P. Dias‐Pereira , I. Amorim , and F. Gärtner , “Retrospective Study of Canine Cutaneous Tumors Submitted to a Diagnostic Pathology Laboratory in Northern Portugal (2014–2020),” Canine Medicine and Genetics 9, no. 1 (2022): 2. 35216632 10.1186/s40575-022-00113-w PMC8875941

[25] J. Rodríguez , Á. Santana , M. A. Borzollino , P. Herráez , D. R. Killick , and A. E. de los Monteros , “Epidemiology of Canine Cutaneous Round Cell Tumours on the Canary Archipelago in Spain,” Veterinary and Comparative Oncology 21, no. 3 (2023): 406–418. 37143410 10.1111/vco.12899

[26] J. A. Villamil , C. J. Henry , J. N. Bryan , et al., “Identification of the Most Common Cutaneous Neoplasms in Dogs and Evaluation of Breed and Age Distributions for Selected Neoplasms,” Journal of the American Veterinary Medical Association 239, no. 7 (2011): 960–965. 21961635 10.2460/javma.239.7.960

[27] B. Pakhrin , M. S. Kang , I. H. Bae , et al., “Retrospective Study of Canine Cutaneous Tumors in Korea,” Journal of Veterinary Science 3, no. 8 (2007): 229–236. 10.4142/jvs.2007.8.3.229 PMC2868128 17679768

[28] The University of Queensland , “ACARCinom,” accessed March 21, 2025, https://www.acarcinom.org.au/.

[29] V. S. Tamlin , C. D. K. Bottema , and A. E. Peaston , “Comparative Aspects of Mast Cell Neoplasia in Animals and the Role of KIT in Prognosis and Treatment,” Veterinary Medicine and Science 6, no. 1 (2020): 3–18. 31650704 10.1002/vms3.201 PMC7036313

[30] M. Willmann , E. Hadzijusufovic , O. Hermine , et al., “Comparative Oncology: The Paradigmatic Example of Canine and Human Mast Cell Neoplasms,” Veterinary and Comparative Oncology 17, no. 1 (2019): 1–10. 30136349 10.1111/vco.12440 PMC6378619

[31] K. Pinello , V. Baldassarre , K. Steiger , et al., “Vet‐ICD‐O‐Canine‐1, a System for Coding Canine Neoplasms Based on the Human ICD‐O‐3.2,” Cancers (Basel) 14, no. 6 (2022): 1529. 35326681 10.3390/cancers14061529 PMC8946502

[32] D. J. Meuten , Tumors in Domestic Animals, 5th ed., ed. D. J. Meuten and D. J. Meuten (John Wiley & Sons, Inc, 2017).

[33] D. De Biase , V. Baldassarre , G. Piegari , et al., “Animal Sentinels and Cancer Registries: State of the Art and New Perspectives,” Annals of Research in Oncology 3, no. 1 (2023): 14.

[34] D. G. O'Neill , G. L. Edmunds , J. Urquhart‐Gilmore , et al., “Dog Breeds and Conformations Predisposed to Osteosarcoma in the UK: A VetCompass Study,” Canine Medicine and Genetics 10, no. 1 (2023): 8. 37365662 10.1186/s40575-023-00131-2 PMC10294386

[35] K. Grüntzig , R. Graf , M. Hässig , et al., “The Swiss Canine Cancer Registry: A Retrospective Study on the Occurrence of Tumours in Dogs in Switzerland From 1955 to 2008,” Journal of Comparative Pathology 152, no. 2–3 (2015): 161–171. 25824119 10.1016/j.jcpa.2015.02.005

[36] A. Śmiech , W. Lsopuszyński , B. Ślaska , K. Bulak , and A. Jasik , “Occurrence and Distribution of Canine Cutaneous Mast Cell Tumour Characteristics Among Predisposed Breeds,” Journal of Veterinary Research 63, no. 1 (2019): 141–148. 30989146 10.2478/jvetres-2019-0002 PMC6458547

[37] R. Graf , A. Pospischil , F. Guscetti , D. Meier , M. Welle , and M. Dettwiler , “Cutaneous Tumors in Swiss Dogs: Retrospective Data From the Swiss Canine Cancer Registry, 2008–2013,” Veterinary Pathology 55, no. 6 (2018): 809–820. 30131007 10.1177/0300985818789466

[38] M. K. Kok , J. K. Chambers , M. Tsuboi , et al., “Retrospective Study of Canine Cutaneous Tumors in Japan, 2008–2017,” Journal of Veterinary Medical Science 81, no. 8 (2019): 1133–1143. 31257236 10.1292/jvms.19-0248 PMC6715907

[39] B. Artuković , L. Medven , M. Hohšteter , et al., “Prevalence of Cutaneous Mast Cell Sarcoma in Dogs in Croatia,” Veterinarski Arhiv 84, no. 6 (2014): 601–614.

[40] H. Mochizuki , A. Motsinger‐Reif , C. Bettini , S. Moroff , and M. Breen , “Association of Breed and Histopathological Grade in Canine Mast Cell Tumours,” Veterinary and Comparative Oncology 15, no. 3 (2017): 829–839. 27198171 10.1111/vco.12225

[41] J. A. Peters , E. Area , and E. Crippen , “Canine Mastocytoma: Excess Risk as Related to Ancestry,” JNCI Journal of the National Cancer Institute 42, no. 3 (1969): 435–443. 4975928

[42] B. D. Reynolds , M. J. Thomson , K. O'Connell , E. J. Morgan , and B. Gummow , “Patient and Tumour Factors Influencing Canine Mast Cell Tumour Histological Grade and Mitotic Index,” Veterinary and Comparative Oncology 17, no. 3 (2019): 338–344. 30891882 10.1111/vco.12477

[43] A. Şmiech , B. Şlaska , W. Łopuszyński , A. Jasik , D. Bochyńska , and R. Da¸browski , “Epidemiological Assessment of the Risk of Canine Mast Cell Tumours Based on the Kiupel Two‐Grade Malignancy Classification,” Acta Veterinaria Scandinavica 60, no. 1 (2018): 70. 30390687 10.1186/s13028-018-0424-2 PMC6215678

[44] A. Pierini , G. Lubas , E. Gori , D. Binanti , F. Millanta , and V. Marchetti , “Epidemiology of Breed‐Related Mast Cell Tumour Occurrence and Prognostic Significance of Clinical Features in a Defined Population of Dogs in West‐Central Italy,” Veterinary Sciences 6, no. 2 (2019): 53. 31174330 10.3390/vetsci6020053 PMC6631885

[45] B. Ansbro and J. M. Riley , “Trends by Race and Ethnicity in Incidence and Mortality of Acral Lentiginous Melanoma: Analysis of Surveillance, Epidemiology, and End Results 2000–2020,” Archives of Dermatological Research 316, no. 7 (2024): 456. 38967822 10.1007/s00403-024-03167-x

[46] D. M. Holman , J. B. King , A. White , S. D. Singh , and J. L. Lichtenfeld , “Acral Lentiginous Melanoma Incidence by Sex, Race, Ethnicity, and Stage in the United States, 2010–2019,” Preventive Medicine 175 (2023): 107692. 37659614 10.1016/j.ypmed.2023.107692 PMC10949133

[47] S. S. Yde , P. Sjoegren , M. Heje , and L. B. Stolle , “Mucosal Melanoma: A Literature Review,” Current Oncology Reports 20, no. 3 (2018): 28. 29569184 10.1007/s11912-018-0675-0

[48] M. Mihajlovic , S. Vlajkovic , P. Jovanovic , and V. Stefanovic , “Primary Mucosal Melanomas: A Comprehensive Review,” International Journal of Clinical and Experimental Pathology 5 (2012): 739–753. 23071856 PMC3466987

[49] K. Saginala , A. Barsouk , J. S. Aluru , P. Rawla , and A. Barsouk , “Epidemiology of Melanoma,” Medical Science 9, no. 4 (2021): 63. 10.3390/medsci9040063 PMC8544364 34698235

[50] J. M. Grassinger , A. Floren , T. Müller , et al., “Digital Lesions in Dogs: A Statistical Breed Analysis of 2912 Cases,” Veterinary Sciences 8, no. 7 (2021): 136. 34357928 10.3390/vetsci8070136 PMC8310350

[51] L. B. Brønden , T. Eriksen , and A. T. Kristensen , “Oral Malignant Melanomas and Other Head and Neck Neoplasms in Danish Dogs – Data From the Danish Veterinary Cancer Registry,” Acta Veterinaria Scandinavica 51 (2009): 54. 20021647 10.1186/1751-0147-51-54 PMC2803174

[52] M. J. Jager , C. L. Shields , C. M. Cebulla , et al., “Uveal Melanoma,” Nature Reviews. Disease Primers 6, no. 24 (2020): 24. 10.1038/s41572-020-0158-0 32273508

[53] L. van der Weyden , T. Brenn , E. E. Patton , G. A. Wood , and D. J. Adams , “Spontaneously Occurring Melanoma in Animals and Their Relevance to Human Melanoma,” Journal of Pathology 252, no. 1 (2020): 4–21. 32652526 10.1002/path.5505 PMC7497193

[54] M. Gillard , E. Cadieu , C. De Brito , et al., “Naturally Occurring Melanomas in Dogs as Models for Non‐UV Pathways of Human Melanomas,” Pigment Cell & Melanoma Research 27, no. 1 (2014): 90–102. 24112648 10.1111/pcmr.12170

[55] K. Huang , J. Fan , and S. Misra , “Acral Lentiginous Melanoma: Incidence and Survival in the United States, 2006‐2015, an Analysis of SEER Registry,” Journal of Surgical Research 251 (2020): 329–339. 32208196 10.1016/j.jss.2020.02.010

[56] S. C. Aguas , M. C. Quarracino , A. N. Lence , H. E. Lanfranchi‐Tizeira , and R. Argentina , “Med Oral Patol Oral Cir Bucal,” Medicina Oral, Patología Oral y Cirugía Bucal 14, no. 6 (2009): 265–271, http://www.medicinaoral.com/medoralfree01/v14i6/medoralv14i6p265.pdf. 19300378

[57] B. A. Krantz , N. Dave , K. M. Komatsubara , B. P. Marr , and R. D. Carvajal , “Uveal Melanoma: Epidemiology, Etiology, and Treatment of Primary Disease,” Clinical Ophthalmology 11 (2017): 279–289. 28203054 10.2147/OPTH.S89591 PMC5298817

[58] C. Pandiani , G. E. Béranger , J. Leclerc , R. Ballotti , and C. Bertolotto , “Focus on Cutaneous and Uveal Melanoma Specificities,” Genes & Development 15, no. 31 (2017): 8. 10.1101/gad.296962.117 PMC5435887 28512236

[59] M. C. Sergi , E. Filoni , G. Triggiano , et al., “Mucosal Melanoma: Epidemiology, Clinical Features, and Treatment,” Current Oncology Reports 25, no. 11 (2023): 1247–1258. 37773078 10.1007/s11912-023-01453-x PMC10640506

[60] P. C. Schultheiss , “A Retrospective Study of Visceral and Nonvisceral Hemangiosarcoma and Hemangiomas in Domestic Animals,” Journal of Veterinary Diagnostic Investigation 16, no. 6 (2004): 522–526. 15586567 10.1177/104063870401600606

[61] A. Hillman , B. Swafford , C. Delavenne , H. Fieten , K. Boerkamp , and K. Tietje , “Descriptive Analysis of Haemangiosarcoma Occurrence in Dogs Enrolled in the Golden Retriever Lifetime Study,” Veterinary and Comparative Oncology 21, no. 4 (2023): 700–708. 37635246 10.1111/vco.12933

[62] A. M. Hargis , P. J. Ihrke , W. L. Spangler , and A. A. Stannard , A Retrospective Clinicopathologic Study of 212 Dogs With Cutaneous Hemangiomas and Hemangiosarcomas. 10.1177/030098589202900406 1514218

[63] A. B. De Nardi , C. de Oliveira Massoco Salles Gomes , C. E. Fonseca‐Alves , et al., “Diagnosis, Prognosis, and Treatment of Canine Hemangiosarcoma: A Review Based on a Consensus Organized by the Brazilian Association of Veterinary Oncology, ABROVET,” Cancers (Basel) 15, no. 7 (2023): 2025. 37046686 10.3390/cancers15072025 PMC10093745

[64] B. R. Lee , A. R. Brostek , and B. H. Kaffenberger , “Ambient UV Radiation Is Associated With Cutaneous Angiosarcoma Incidence in the United States, 1992 to 2020,” Journal of the American Academy of Dermatology 91, no. 1 (2024): 102–104. 38432461 10.1016/j.jaad.2024.01.084 PMC11193604

[65] H. G. Parker , D. L. Dreger , M. Rimbault , et al., “Genomic Analyses Reveal the Influence of Geographic Origin, Migration, and Hybridization on Modern Dog Breed Development,” Cell Reports 19, no. 4 (2017): 697–708. 28445722 10.1016/j.celrep.2017.03.079 PMC5492993

[66] B. J. Diessner , B. J. Weigel , P. Murugan , L. Zhang , J. N. Poynter , and L. G. Spector , “Racial and Ethnic Differences in Sarcoma Incidence Are Independent of Census‐Tract Socioeconomic Status,” Cancer Epidemiology, Biomarkers & Prevention 29, no. 11 (2020): 2141–2148. 10.1158/1055-9965.EPI-20-0520 PMC7641997 32928933

[67] G. L. Edmunds , M. J. Smalley , S. Beck , et al., “Dog Breeds and Body Conformations With Predisposition to Osteosarcoma in the UK: A Case‐Control Study,” Canine Medicine and Genetics 10, no. 80 (2021): 1. 10.1186/s40575-021-00100-7 PMC7944903 33750475

[68] K. Grüntzig , R. Graf , G. Boo , et al., “Swiss Canine Cancer Registry 1955–2008: Occurrence of the Most Common Tumour Diagnoses and Influence of Age, Breed, Body Size, Sex and Neutering Status on Tumour Development,” Journal of Comparative Pathology 155, no. 2–3 (2016): 156–170. 27406312 10.1016/j.jcpa.2016.05.011

[69] F. Pu , H. Guo , D. Shi , et al., “The Generation and Use of Animal Models of Osteosarcoma in Cancer Research,” Genes & Diseases 11, no. 2 (2024): 664–674. 37692517 10.1016/j.gendis.2022.12.021 PMC10491873

[70] M. Zeng , C. Liu , H. Gong , et al., “Therapeutic Potential of Tyrosine‐Protein Kinase MET in Osteosarcoma,” Frontiers in Molecular Biosciences 26, no. 11 (2024): 1367331. 10.3389/fmolb.2024.1367331 PMC11002252 38596618

[71] A. T. Liao , M. McMahon , and C. A. London , “Identification of a Novel Germline MET Mutation in Dogs,” Animal Genetics 37, no. 3 (2006): 248–252. 16734685 10.1111/j.1365-2052.2006.01415.x

[72] C. R. White , A. E. Hohenhaus , J. Kelsey , and E. Procter‐Gray , “Cutaneous MCTs: Associations With Spay/Neuter Status, Breed, Body Size, and Phylogenetic Cluster,” Journal of the American Animal Hospital Association 47, no. 3 (2011): 210–216. 21498594 10.5326/JAAHA-MS-5621

[73] W. A. Ware and D. L. Hopper , “Cardiac Tumors in Dogs: 1982‐1995,” Journal of Veterinary Internal Medicine 13, no. 2 (1999): 95–103. 10225598 10.1892/0891-6640(1999)013<0095:ctid>2.3.co;2

[74] M. M. Mielke , E. Kapoor , J. R. Geske , et al., “Long‐Term Effects of Premenopausal Bilateral Oophorectomy With or Without Hysterectomy on Physical Aging and Chronic Medical Conditions,” Menopause 30, no. 11 (2023): 1090–1097. 37699239 10.1097/GME.0000000000002254 PMC10615715

[75] M. Gottschau , S. Rosthøj , A. Settnes , et al., “Long‐Term Health Consequences After Ovarian Removal at Benign Hysterectomy A Nationwide Cohort Study,” Annals of Internal Medicine 176, no. 5 (2023): 596–604. 37068275 10.7326/M22-1628

[76] M. A. Kutzler , “Possible Relationship Between Long‐Term Adverse Health Effects of Gonad‐Removing Surgical Sterilization and Luteinizing Hormone in Dogs,” Animals 10, no. 4 (2020): 599. 32244716 10.3390/ani10040599 PMC7222805

[77] M. A. Kutzler , “Understanding the Effects of Sustained Supraphysiologic Concentrations of Luteinizing Hormone in Gonadectomized Dogs: What We Know and What We Still Need to Learn,” Theriogenology 196 (2023): 270–274. 36459946 10.1016/j.theriogenology.2022.11.007

[78] N. Bohm‐Levine , A. R. Goldberg , M. Mariani , M. Frankfurt , and J. Thornton , “Reducing Luteinizing Hormone Levels After Ovariectomy Improves Spatial Memory: Possible Role of Brain‐Derived Neurotrophic Factor,” Hormones and Behavior 118 (2020): 104590. 31593698 10.1016/j.yhbeh.2019.104590

[79] J. P. Caron , A. P. Bennet , M. M. Plantavid , and J. P. Louvet , “Luteinizing Hormone Secretory Pattern Before and After Removal of Leydig Cell Tumor of the Testis,” European Journal of Endocrinology 131, no. 2 (1994): 156–159. 8075784 10.1530/eje.0.1310156

[80] J. E. Pabon , J. S. Bird , X. Li , et al., “Human Skin Contains Luteinizing Hormone/Chorionic Gonadotropin Receptors,” Journal of Clinical Endocrinology and Metabolism 81, no. 7 (1996): 2738–2741, https://academic.oup.com/jcem/article/81/7/2738/2875582. 8675605 10.1210/jcem.81.7.8675605

[81] K. H. Zwida , B. A. Valentine , and M. A. Kutzler , “Immunohistochemical Localization of LH Receptors in Canine Splenic Hemangiosarcoma,” Journal of Veterinary Science & Animal Husbandry 6, no. 4 (2018): 410.

[82] K. Reisinger , N. Baal , T. McKinnon , K. Münstedt , and M. Zygmunt , “The Gonadotropins: Tissue‐Specific Angiogenic Factors?,” Molecular and Cellular Endocrinology 269, no. 1‐2 (2007): 65–80. 17349737 10.1016/j.mce.2006.11.015

[83] C. V. Rao and Z. M. Lei , “The Past, Present and Future of Nongonadal LH/hCG Actions in Reproductive Biology and Medicine,” Molecular and Cellular Endocrinology 269, no. 1‐2 (2007): 2–8. 17382462 10.1016/j.mce.2006.07.007

[84] F. Sánchez‐Vizcaíno , P. J. M. Noble , P. H. Jones , et al., “Demographics of Dogs, Cats, and Rabbits Attending Veterinary Practices in Great Britain as Recorded in Their Electronic Health Records,” BMC Veterinary Research 13, no. 1 (2017): 218. 28693574 10.1186/s12917-017-1138-9 PMC5504643

[85] K. Rigas , D. A. Singleton , A. D. Radford , I. Amores‐Fuster , and D. R. Killick , “Do Socioeconomic Factors Impact Management of Suspected Canine Multicentric Lymphoma in UK First Opinion Practice?,” Veterinary Record 191, no. 4 (2022): e1319. 35191051 10.1002/vetr.1319

[86] H. Davies , G. Nenadic , G. Alfattni , et al., “Text Mining for Disease Surveillance in Veterinary Clinical Data: Part One, the Language of Veterinary Clinical Records and Searching for Words,” Frontiers in Veterinary Science 11 (2024): 1352239. 38322169 10.3389/fvets.2024.1352239 PMC10844486

[87] O. Jaber , K. Ammar , and M. Sughayer , “Communicating Uncertainty in Pathology Reports: A Descriptive Study From a Specialized Cancer Center,” Academic Pathology 11, no. 1 (2024): 100109. 38433775 10.1016/j.acpath.2024.100109 PMC10907152

[88] S. L. Smith , M. M. Afonso , L. Roberts , P. J. M. Noble , G. L. Pinchbeck , and A. D. Radford , “A Virtual Biobank for Companion Animals: A Parvovirus Pilot Study,” Veterinary Record 189, no. 6 (2021): e556. 34101190 10.1002/vetr.556

