# Supplementary material for: Epidemiology of Four Major Canine Tumours in the UK: Insights From a National Pathology Registry With Comparative Oncology Perspectives
Source: Vet Comp Oncol. 2026 Feb 24;24(2):324–40. doi: 10.1111/vco.70056 (PMC13161743; doi:10.1111/vco.70056)
Supplement: Supplementary file 1 — Data S1: Supporting Information. [file VCO-24-324-s002.docx]

# Supplementary material

Contents

[Supplementary material 1](#_Toc214869811)

[Supplementary Figures (n=17)*.* 4](#_Toc214869812)

[Note to Readers – Supplementary figures. 4](#_Toc214869813)

[Supplementary Figure 1: Number of tumour type diagnosed each year along with the mean of diagnoses per dog per year. 6](#_Toc214869814)

[Supplementary Figure 2: Significant Odds Ratios – Mast cell tumour (main multivariable model). 8](#_Toc214869815)

[Supplementary Figure 3: Significant Odds Ratios – Oral melanoma. 10](#_Toc214869816)

[Supplementary Figure 4: Significant Odds Ratios – Cutaneous melanoma. 11](#_Toc214869817)

[Supplementary Figure 5: Significant Odds Ratios – Cutaneous digital melanoma. 12](#_Toc214869818)

[Supplementary Figure 6: Significant Odds Ratios – Uveal melanoma. 13](#_Toc214869819)

[Supplementary Figure 7: Significant Odds Ratios – Splenic haemangiosarcoma 14](#_Toc214869820)

[Supplementary Figure 8: Significant Odds Ratios – Cutaneous haemangiosarcoma. 15](#_Toc214869821)

[Supplementary Figure 9: Significant Odds Ratios – Osteosarcoma. 16](#_Toc214869822)

[Supplementary Figure 10: Sex-specific associations between neuter status and tumour diagnosis across age groups: Mast cell tumour. 17](#_Toc214869823)

[Supplementary Figure 11: Sex-specific associations between neuter status and tumour diagnosis across age groups: Oral melanoma. 18](#_Toc214869824)

[Supplementary Figure 12: Sex-specific associations between neuter status and tumour diagnosis across age groups: Cutaneous melanoma. 19](#_Toc214869825)

[Supplementary Figure 13: Sex-specific associations between neuter status and tumour diagnosis across age groups: Cutaneous digital melanoma. 20](#_Toc214869826)

[Supplementary Figure 14: Sex-specific associations between neuter status and tumour diagnosis across age groups: Uveal melanoma. 21](#_Toc214869827)

[Supplementary Figure 15: Sex-specific associations between neuter status and tumour diagnosis across age groups: Splenic haemangiosarcoma. 22](#_Toc214869828)

[Supplementary Figure 16: Sex-specific associations between neuter status and tumour diagnosis across age groups: Cutaneous haemangiosarcoma. 23](#_Toc214869829)

[Supplementary Figure 17: Sex-specific associations between neuter status and tumour diagnosis across age groups: Osteosarcoma. 24](#_Toc214869830)

[Supplementary Tables (n = 20). 24](#_Toc214869831)

[Note to readers about the supplementary tables. 24](#_Toc214869832)

[Supplementary Table 1: Mean number of diagnoses per dog per year for each tumour type, along with the 95% confidence interval (CI) for the mean. 26](#_Toc214869833)

[Supplementary Table 2: Anatomical distribution of mast cell tumours 26](#_Toc214869834)

[Table 2.a: Distribution of mast cell tumours on the skin and on non-skin locations. 26](#_Toc214869835)

[Table 2.b: Specific anatomical distribution of mast cell tumours 27](#_Toc214869836)

[Supplementary Table 3: Distribution of mast cell tumours according to Patnaik 3-tier scheme 29](#_Toc214869837)

[Supplementary Table 4: Distribution of Mast cell tumours according to Kiupel 2-tier scheme 29](#_Toc214869838)

[Supplementary table 5: Full data table from the multivariable logistic regression analysis for mast cell tumours. 30](#_Toc214869839)

[Supplementary table 6: Full results from the univariable logistic regression analysis for mast cell tumours. Breeds with significantly increased odds of mast cell tumour diagnosis compared to Crossbreeds, based on a model restricted to dogs diagnosed with tumours. 35](#_Toc214869840)

[Supplementary table 7: Breed distribution according to Patnaik 3-tier scheme 40](#_Toc214869841)

[Supplementary table 8: Breed distribution according to according to Kiupel 2-tier scheme 44](#_Toc214869842)

[Supplementary table 9: emmeans for pairwise comparison between sex and neuter status for mast cell tumours. 48](#_Toc214869843)

[Supplementary table 10: Full data table from the multivariable logistic regression analysis for melanomas. 49](#_Toc214869844)

[Table 10.a: Oral melanomas 49](#_Toc214869845)

[Table 10.b: Cutaneous melanomas 53](#_Toc214869846)

[Table 10.c: Cutaneous digital melanomas 56](#_Toc214869847)

[Table 10.d: Uveal melanomas 58](#_Toc214869848)

[Supplementary table 11: emmeans for pairwise comparison between sex and neuter status for melanomas. 59](#_Toc214869849)

[Table 11.a: Oral melanomas 60](#_Toc214869850)

[Table 11.b: Cutaneous melanomas 61](#_Toc214869851)

[Table 11.c: Cutaneous digital melanomas 62](#_Toc214869852)

[Table 11.d: Uveal melanomas 64](#_Toc214869853)

[Supplementary table 12: Anatomical distribution of Haemangiosarcomas 65](#_Toc214869854)

[Supplementary table 13: Full data table from the multivariable logistic regression analysis for haemangiosarcomas. 66](#_Toc214869855)

[Table 13.a: Splenic haemangiosarcoma 66](#_Toc214869856)

[Table 13.b: Cutaneous haemangiosarcoma 69](#_Toc214869857)

[Supplementary table 14: emmeans for pairwise comparison between sex and neuter status for haemangiosarcomas. 72](#_Toc214869858)

[Table 14.a: Splenic haemangiosarcoma 72](#_Toc214869859)

[Table 14.b: Cutaneous haemangiosarcoma 73](#_Toc214869860)

[Supplementary table 15: Anatomical distribution of all osteosarcomas. 74](#_Toc214869861)

[Supplementary table 16: Anatomical distribution of Osteosarcomas in high-odds breeds. 76](#_Toc214869862)

[Supplementary table 17: Anatomical distribution of Osteosarcomas in low-odds breeds. 77](#_Toc214869863)

[Supplementary table 18: Full data table from the multivariable logistic regression analysis for osteosarcomas. 78](#_Toc214869864)

[Supplementary table 19 emmeans for pairwise comparison between sex and neuter status for osteosarcomas. 81](#_Toc214869865)

[Supplementary Statistical Analysis (n=4) 83](#_Toc214869866)

[Note to readers about the statistical test outputs: 83](#_Toc214869867)

[Supplementary statistical analysis for Mast cell tumours 83](#_Toc214869868)

[MCT: Ages 83](#_Toc214869869)

[MCT: Age at diagnosis according to Grade 2-tier 83](#_Toc214869870)

[MCT: Age at diagnosis according to Grade 3-tier 84](#_Toc214869871)

[Supplementary statistical analysis for Melanomas 85](#_Toc214869872)

[Melanoma: Ages. 85](#_Toc214869873)

[Supplementary statistical analysis for Haemangiosarcomas 86](#_Toc214869874)

[Haemangiosarcoma: Ages. 86](#_Toc214869875)

[Supplementary statistical analysis for Osteosarcomas 87](#_Toc214869876)

[Osteosarcoma: Analyzing differences in the anatomical distribution of osteosarcomas between high-risk and low-risk breeds. 87](#_Toc214869877)

[Osteosarcoma: Ages. 87](#_Toc214869878)

[Osteosarcoma: Analizing differences in age at diagnosis in low and high-risk breeds. 88](#_Toc214869879)

[Supplementary Code 89](#_Toc214869880)

[Python code: see Supplementary_material_(Python script_ANNONIMIZED_TR_paper.html) 89](#_Toc214869881)

[Schematic overview of Data extraction (four steps) and normalization process. 90](#_Toc214869882)

[Free-text narratives examples from the original dataset. 90](#_Toc214869883)

[Step 1: Separating Diagnosis section (Python script_ANNONIMIZED_TR_paper, step 4) 92](#_Toc214869884)

[Step 2: Separating individual diagnosis (Python script_ANNONIMIZED_TR_paper, step 5) 93](#_Toc214869885)

[Step 3: Dataset from wider to larger to go from an animal-per row dataset to a diagnosis-per row dataset and use of specific curated dictionaries: match with Keys and mapp to Values (Python script_ANNONIMIZED_TR_paper, step 6 onwards). 94](#_Toc214869886)

[Dictionaries 95](#_Toc214869887)

[Step 4 (end result): Normalized dataset with the mapped terms from the curated dictionaries. 97](#_Toc214869888)

# Supplementary Figures (n=17)*.*

## Note to Readers – Supplementary figures.

- The supplementary figures included in this manuscript are intended to help readers navigate the main epidemiological patterns identified in the study. Full numerical outputs are provided in accompanying supplementary tables to ensure complete transparency and reproducibility.
- **The supplementary figures 2 to 9** provide a visual summary of statistically significant odds ratios (ORs) from our regression analyses. These figures highlight only those breed, sex, or age-related associations that reached statistical significance (*p* < 0.05), facilitating quick identification of key patterns and trends.

For completeness, the corresponding full regression output tables, including all comparisons (regardless of statistical significance), are available in the supplementary tables 5, 11.a-d, 14.a-b and 18).

- **The supplementary figures 10 to 17** present all age-group comparisons for each tumour type, including statistically significant and non-significant associations between neuter status and tumour diagnosis.

As illustrated in Figure 1 of the main manuscript for four representative tumours, for each age range within a given tumour type:

- Red points represent the odds ratios (ORs) comparing female neutered vs female entire (FN vs FE).
- Blue points represent the ORs comparing male neutered vs male entire (MN vs ME).
- Horizontal red and blue bars display the respective 95% confidence intervals.
- The black line connecting the red and blue points shows the difference between sexes in the neutering effect for that age group.
- An asterisk (*) next to the age label indicates that the neutered vs entire contrast was statistically significant in both sexes for that tumour–age combination.
- The vertical dashed line at OR = 1 marks the null value.

For transparency and completeness, the corresponding emmeans output tables are available in the supplementary tables 9, 10.a-d, 13.a-b and 19).

- For clarity, the table below indicates which supplementary output table corresponds to each supplementary figure, ensuring direct traceability between graphical summaries and their underlying statistical results

| **Supplementary Figure** | **Corresponding Supplementary Full data table** |
| --- | --- |
| Suppl. Figure 2 | Suppl. Table 5 |
| Suppl. Figure 3 | Suppl. Table 10.a |
| Suppl. Figure 4 | Suppl. Table 10.b |
| Suppl. Figure 5 | Suppl. Table 10.c |
| Suppl. Figure 6 | Suppl. Table 10.d |
| Suppl. Figure 7 | Suppl. Table 13.a |
| Suppl. Figure 8 | Suppl. Table 13.b |
| Suppl. Figure 9 | Suppl. Table 18 |
| Suppl. Figure 10 | Suppl. Table 9 |
| Suppl. Figure 11 | Suppl. Table 11.a |
| Suppl. Figure 12 | Suppl. Table 11.b |
| Suppl. Figure 13 | Suppl. Table 11.c |
| Suppl. Figure 14 | Suppl. Table 11.d |
| Suppl. Figure 15 | Suppl. Table 14.a |
| Suppl. Figure 16 | Suppl. Table 14.b |
| Suppl. Figure 17 | Suppl. Table 19 |

## Supplementary Figure 1: Number of tumour type diagnosed each year along with the mean of diagnoses per dog per year.


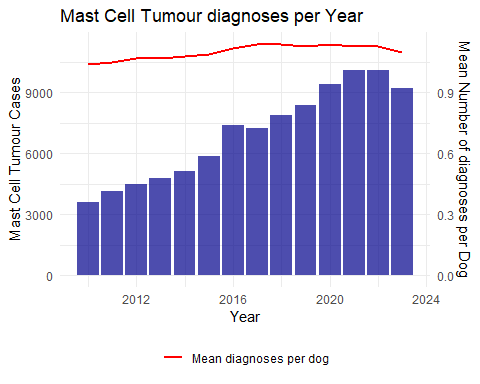

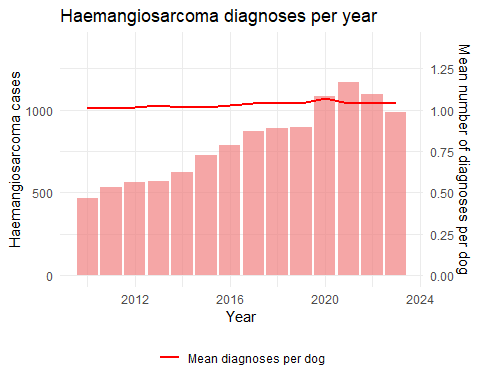

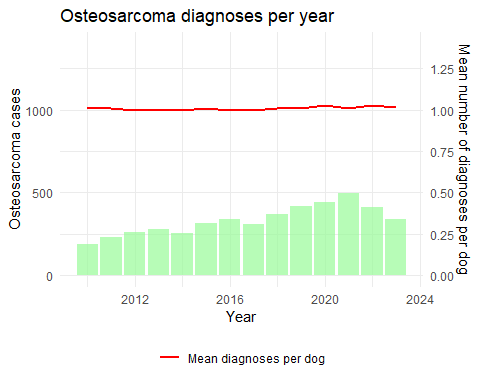

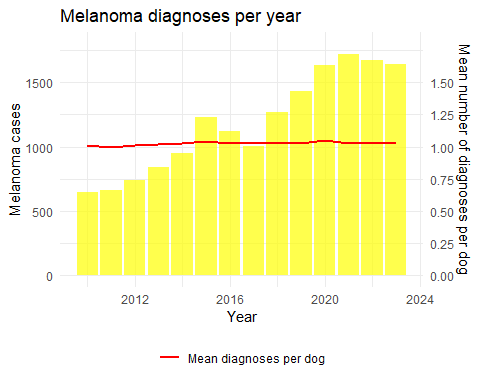


## Supplementary Figure 2: Significant Odds Ratios – Mast cell tumour (main multivariable model).


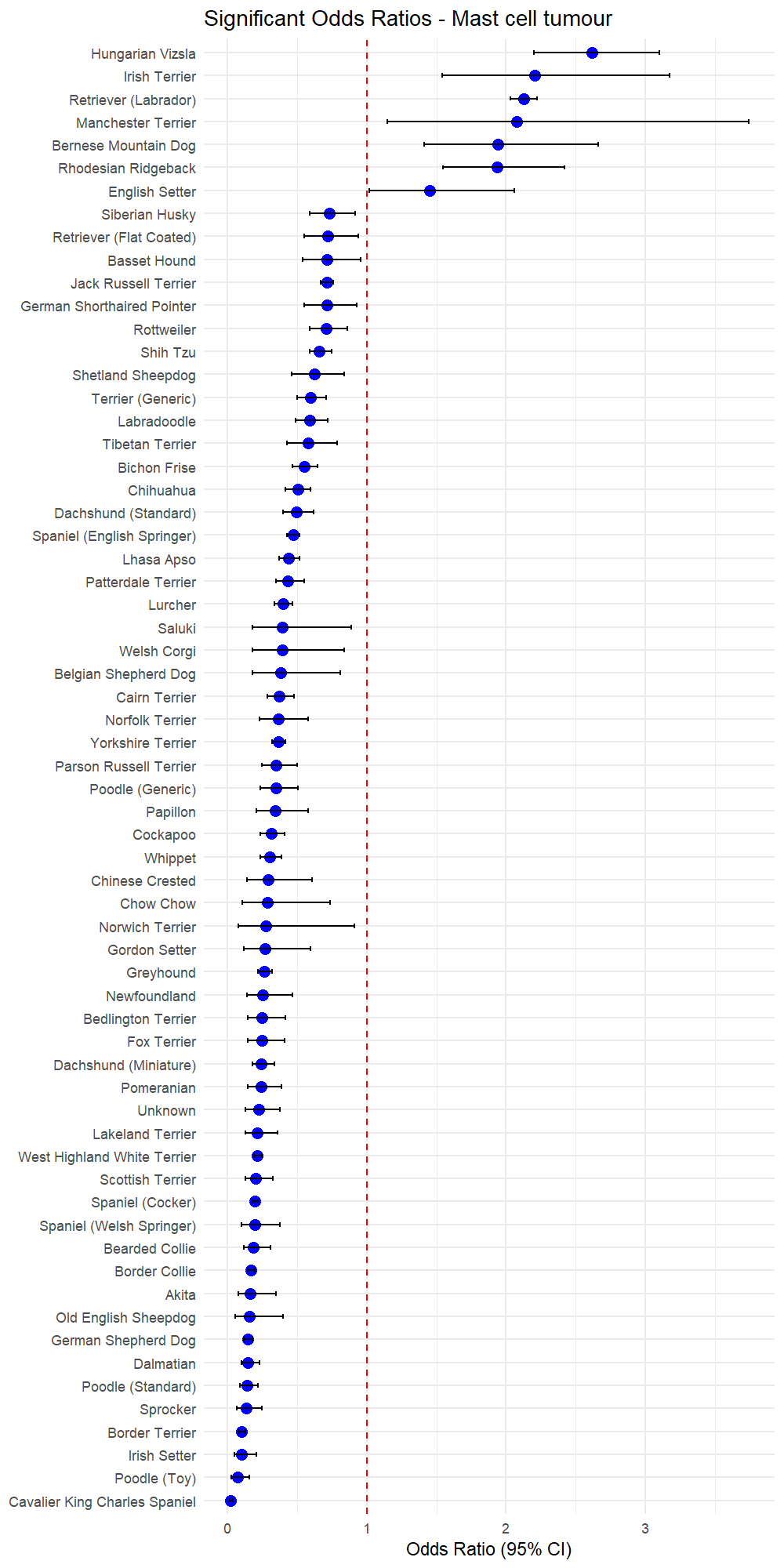


## Supplementary Figure 3: Significant Odds Ratios – Oral melanoma.


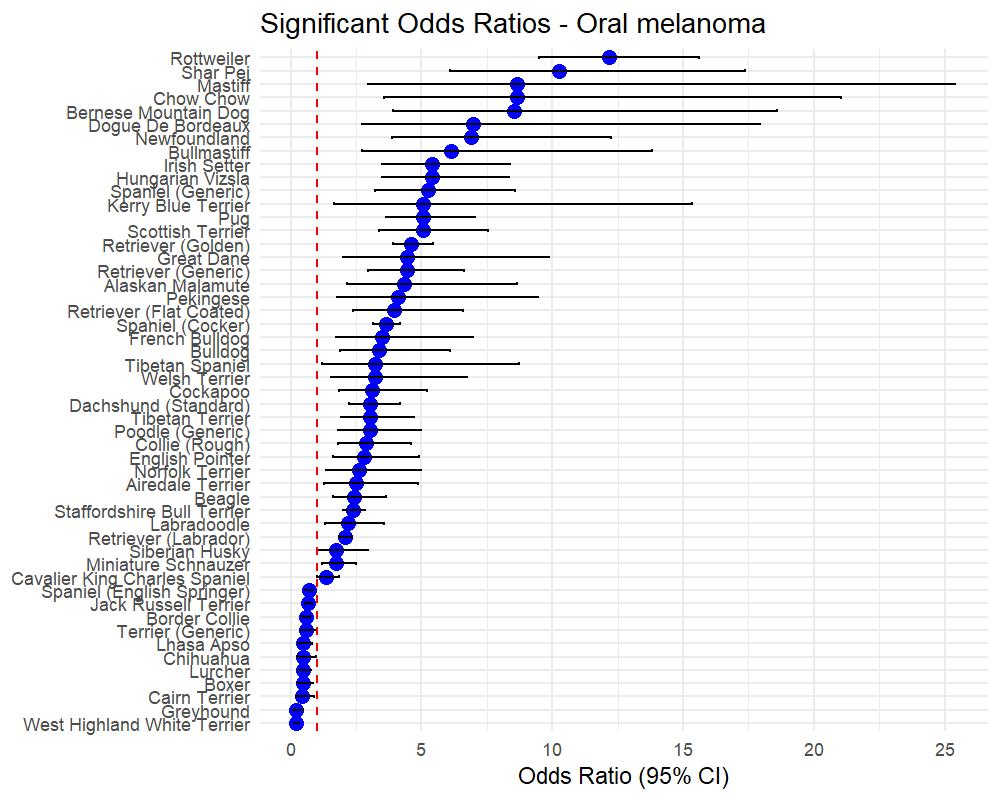


## Supplementary
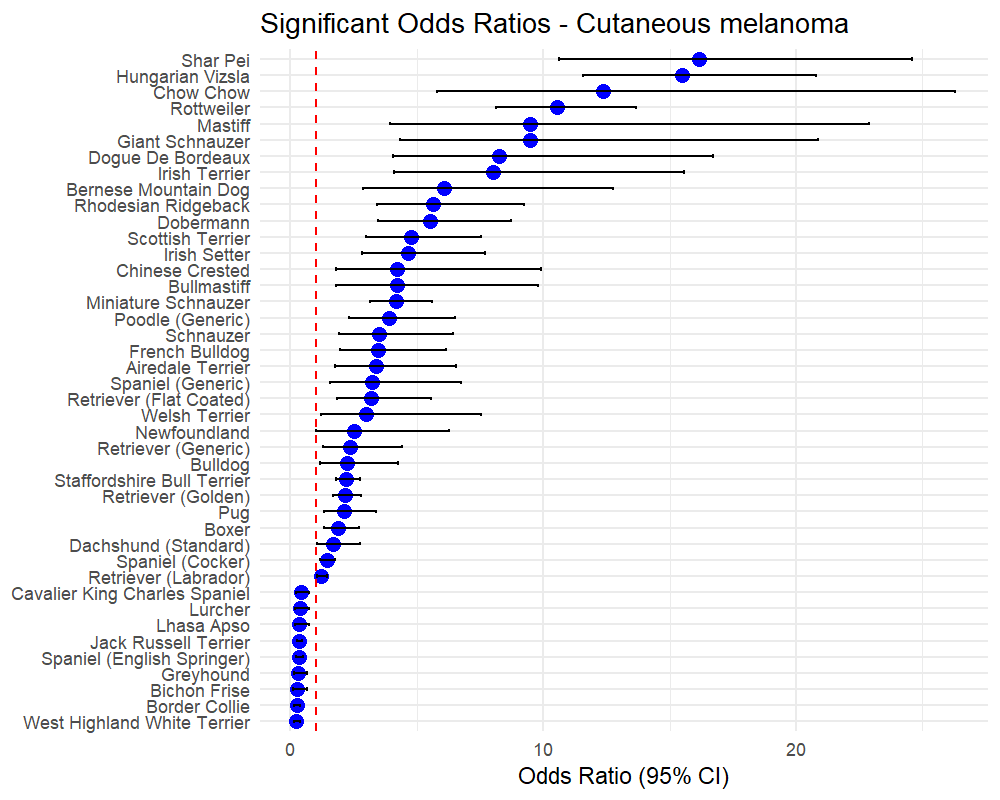
Figure 4: Significant Odds Ratios – Cutaneous melanoma.

## Supplementary
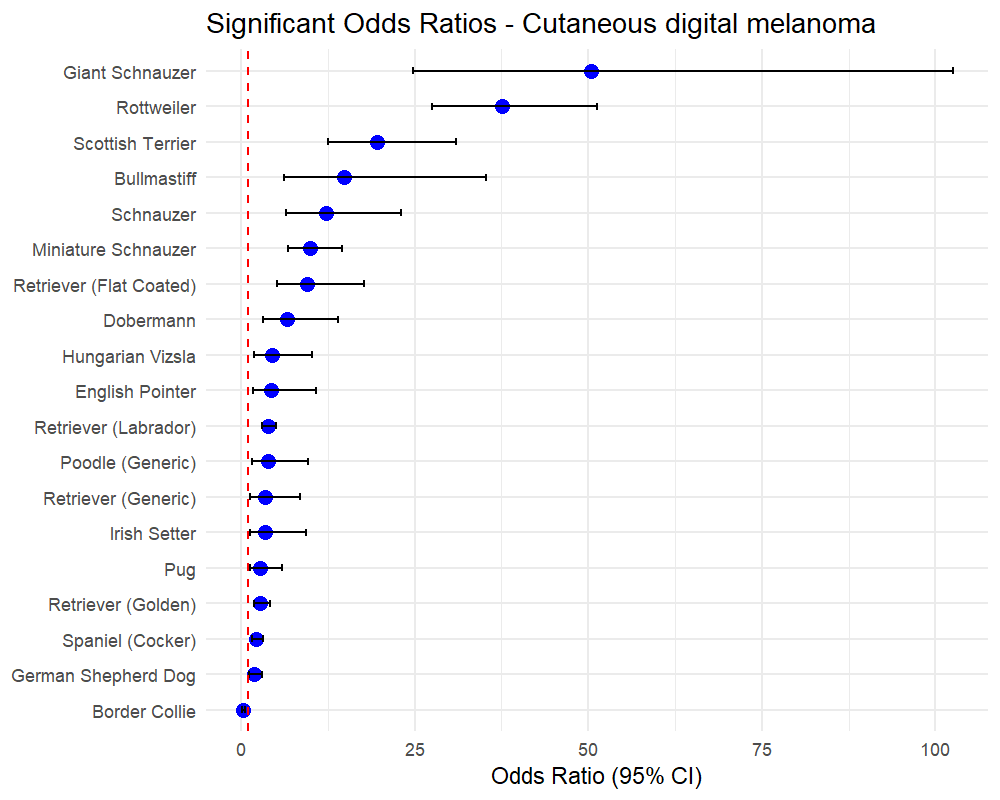
Figure 5: Significant Odds Ratios – Cutaneous digital melanoma.

## Supplementary
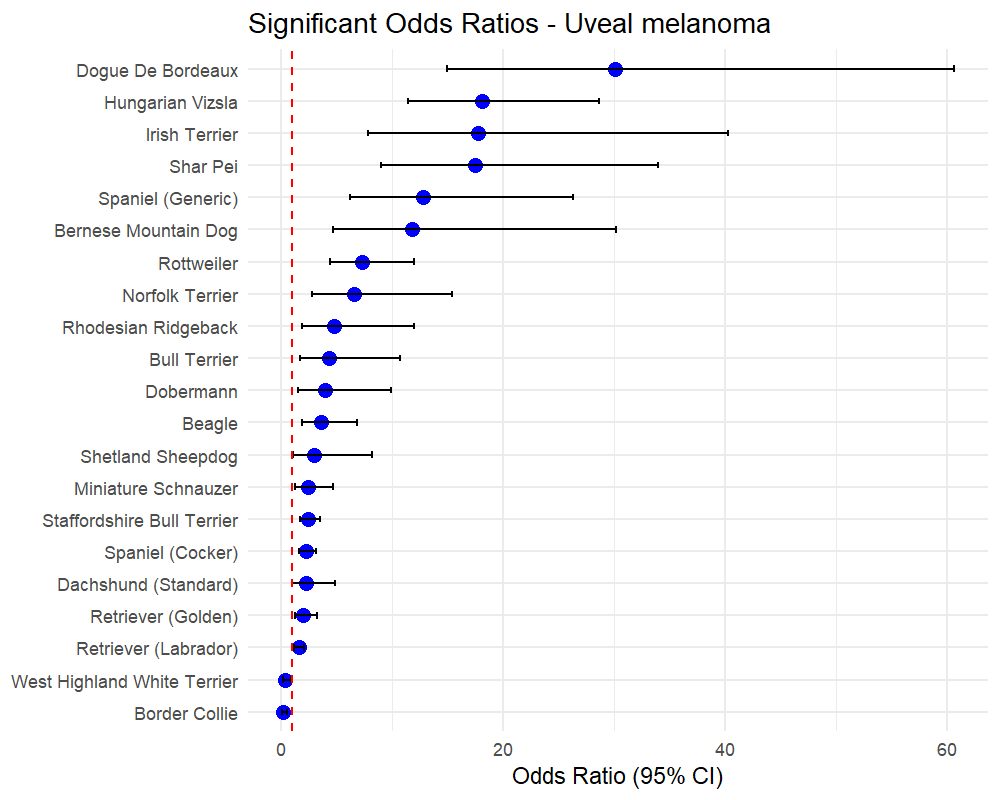
Figure 6: Significant Odds Ratios – Uveal melanoma.

Supplementary
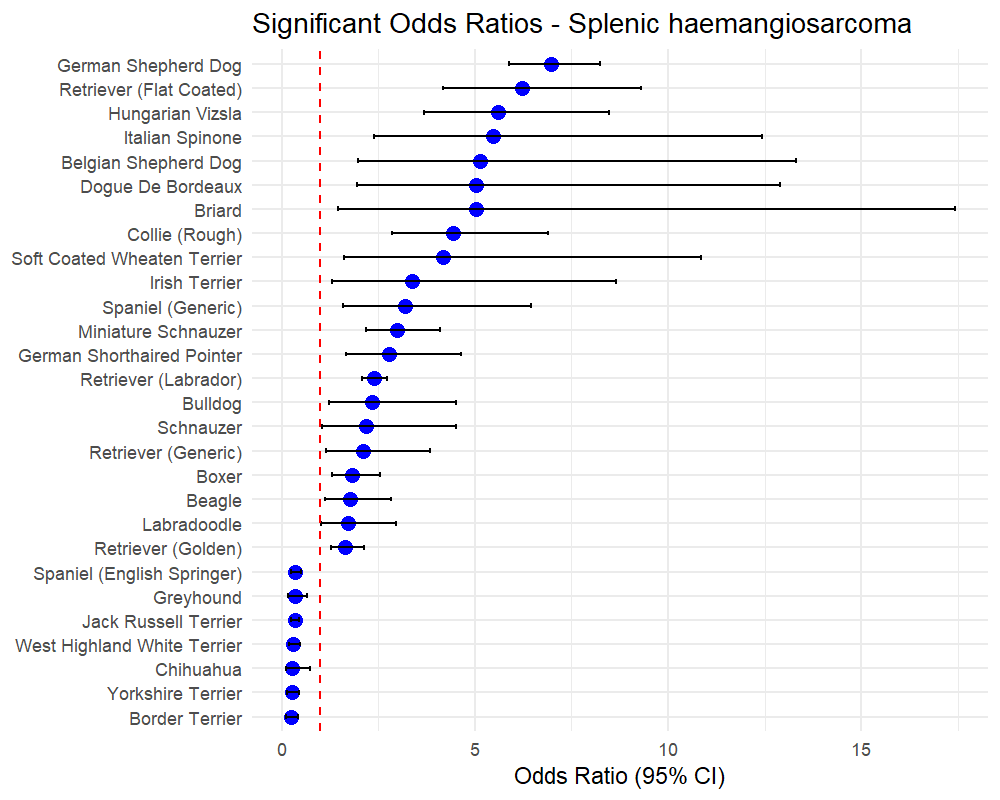
Figure 7: Significant Odds Ratios – Splenic haemangiosarcoma.

## Supplementary Figure 8: Significant Odds Ratios – Cutaneous haemangiosarcoma.


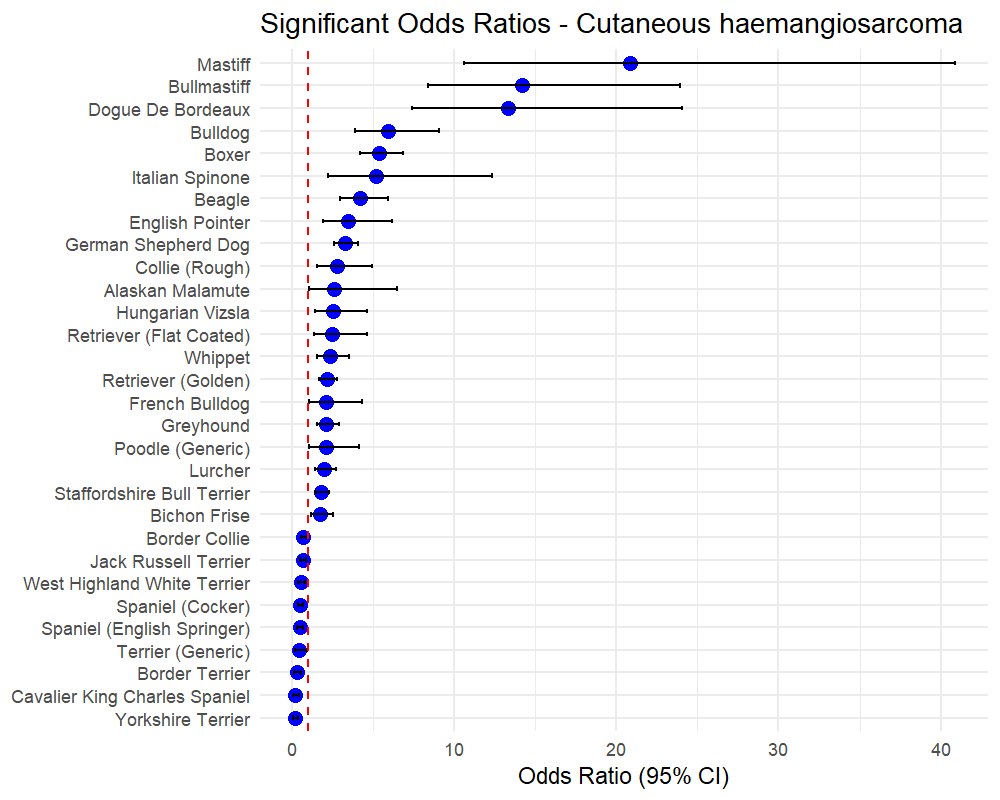


## Supplementary Figure 9: Significant Odds Ratios – Osteosarcoma.


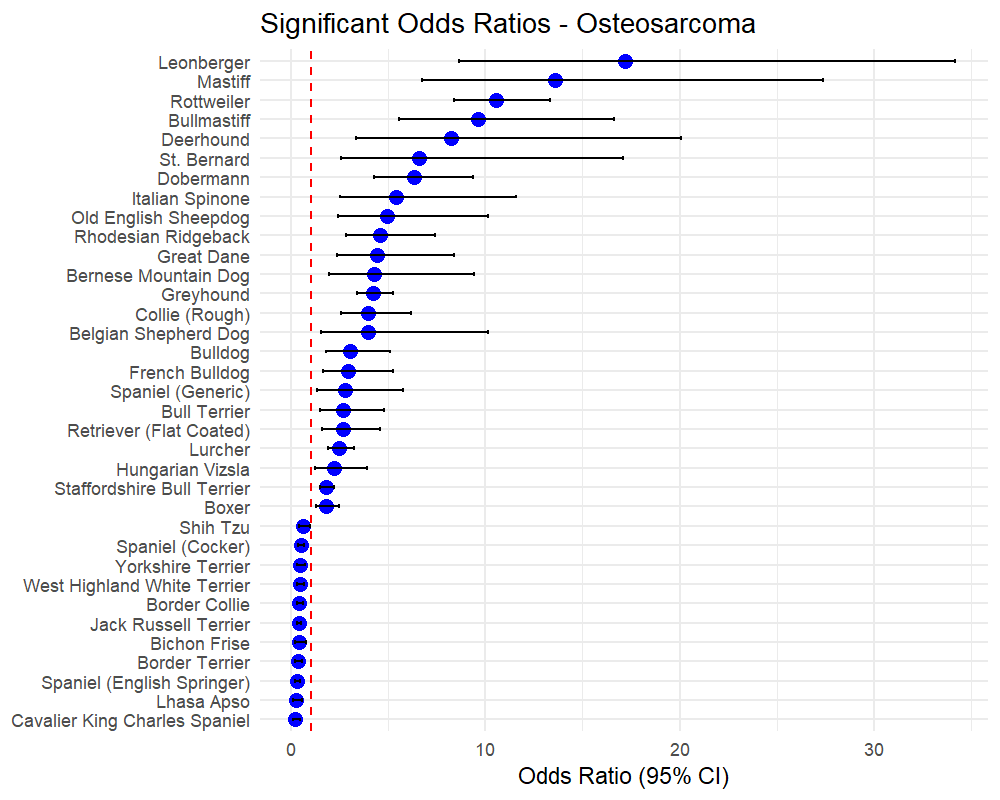


## Supplementary Figure 10: Sex-specific associations between neuter status and tumour diagnosis across age groups: Mast cell tumour.


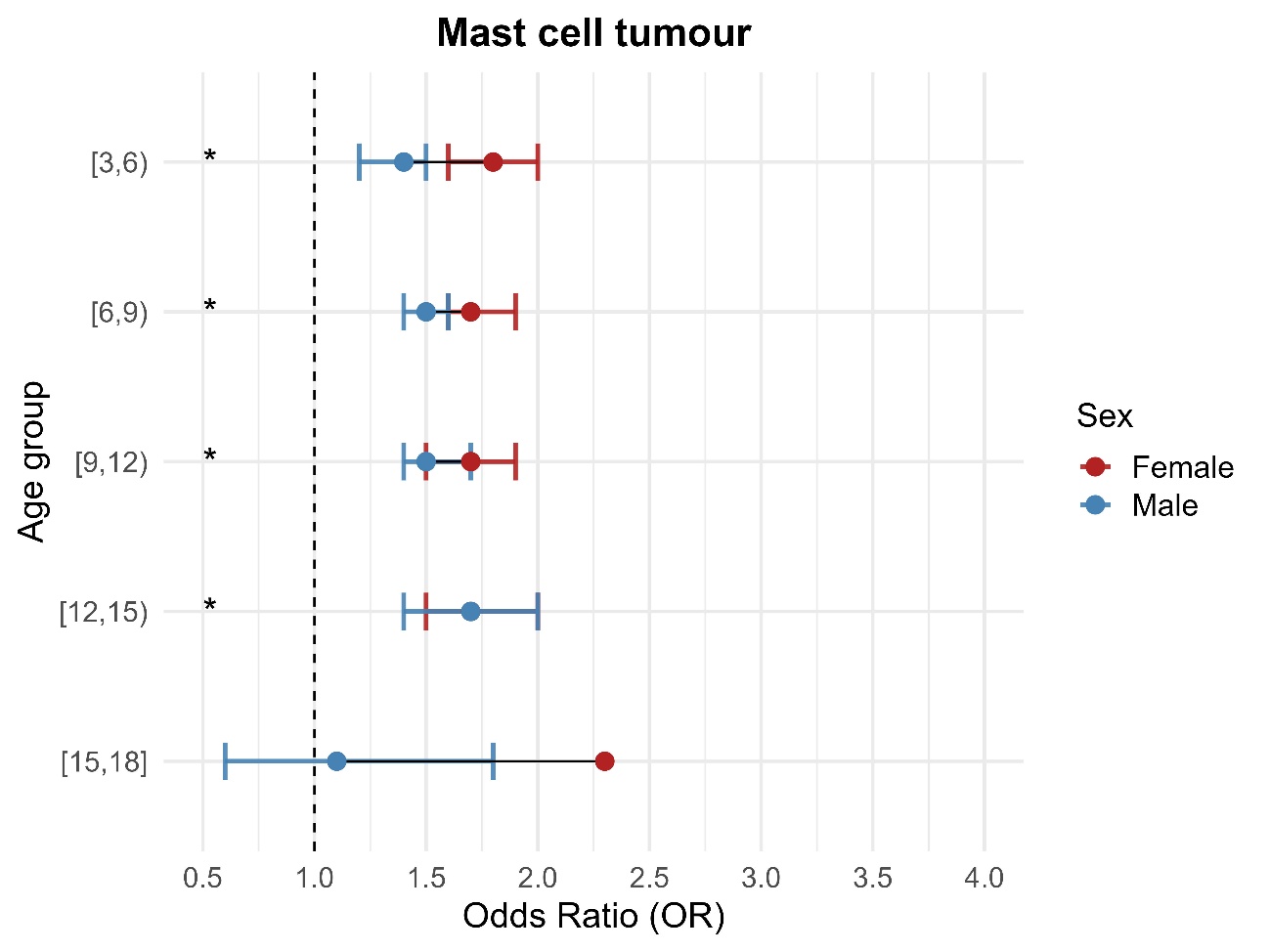


## Supplementary Figure 11: Sex-specific associations between neuter status and tumour diagnosis across age groups: Oral melanoma.


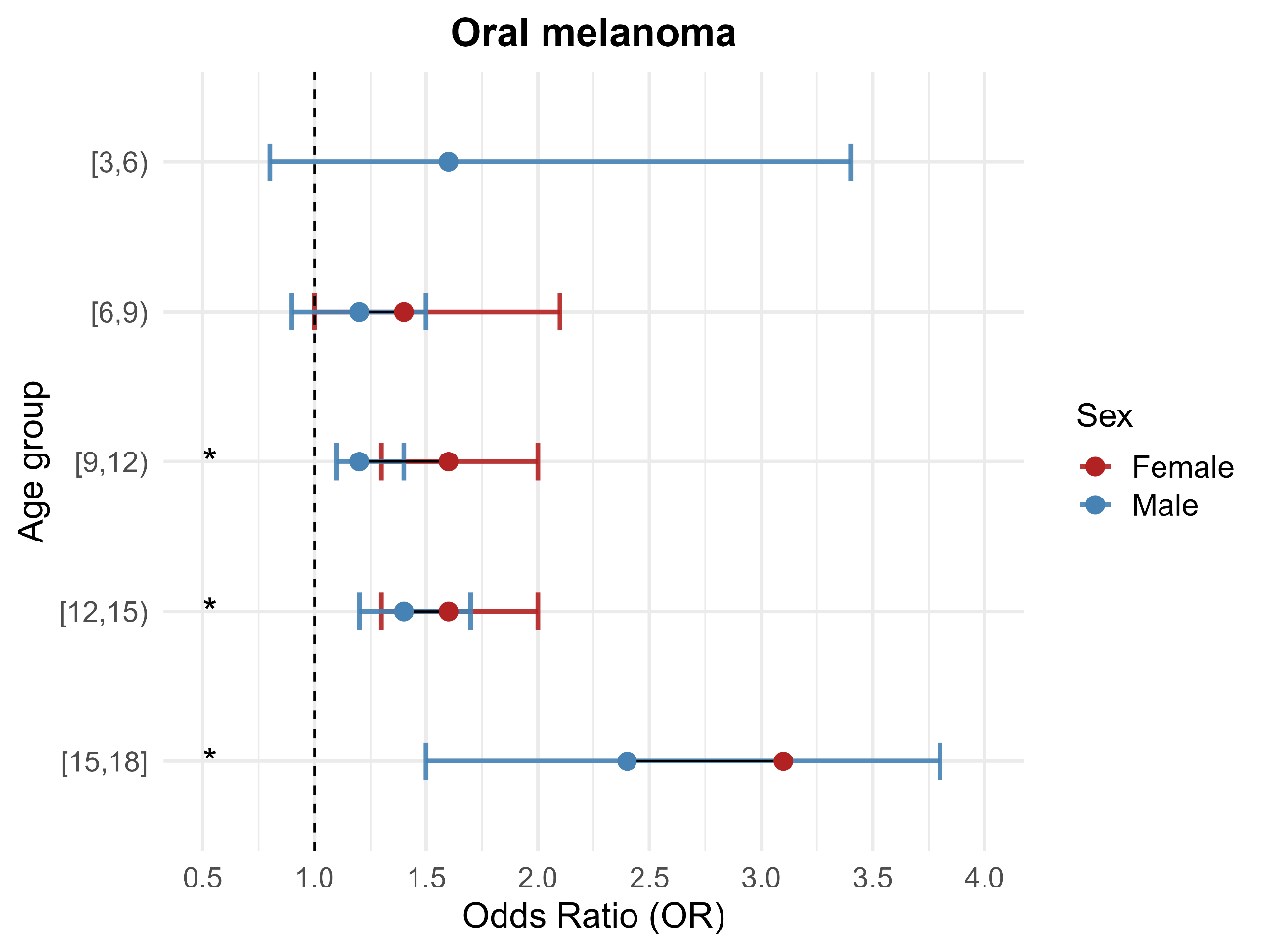


## Supplementary Figure 12: Sex-specific associations between neuter status and tumour diagnosis across age groups: Cutaneous melanoma.


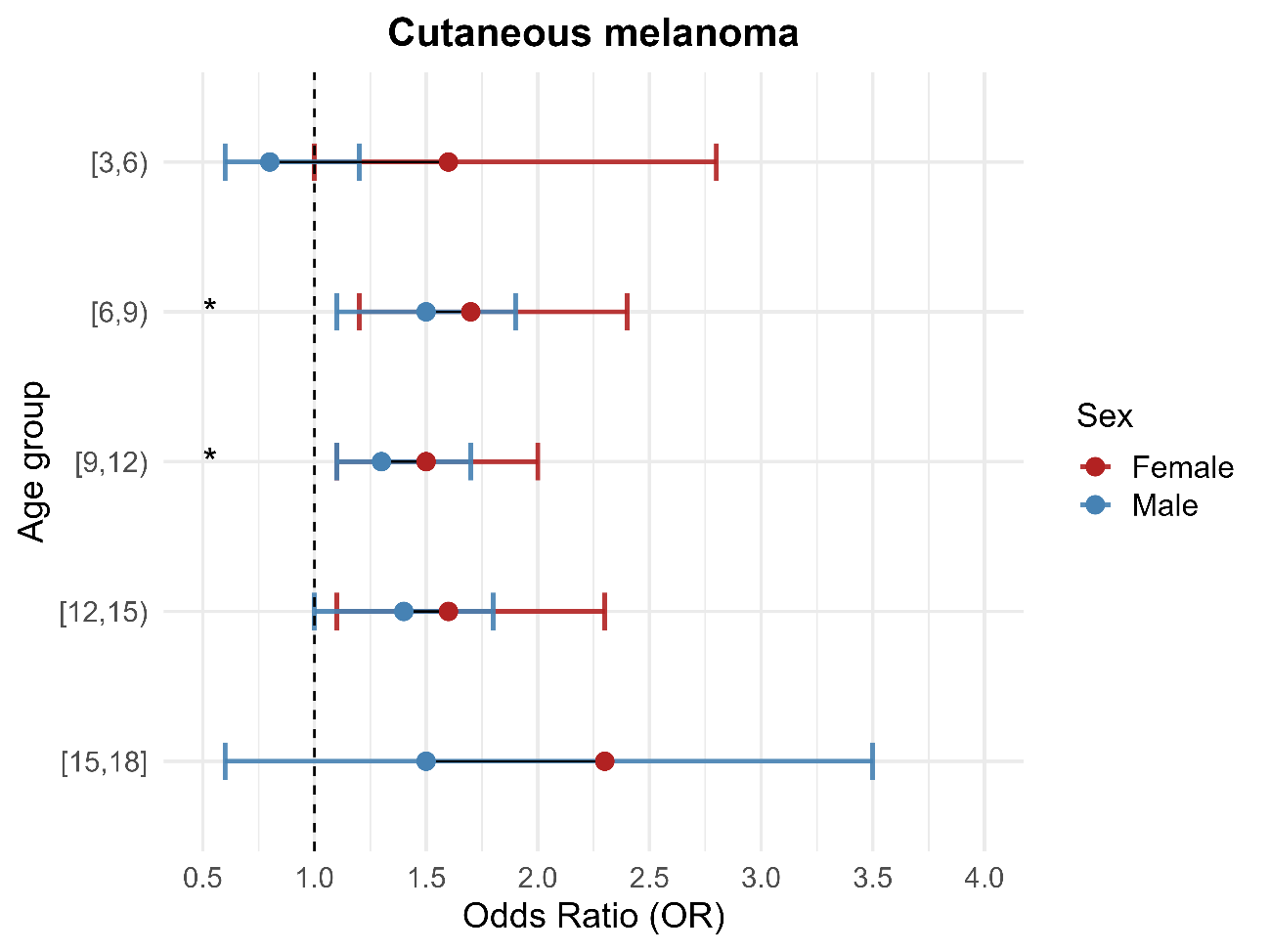


## Supplementary Figure 13: Sex-specific associations between neuter status and tumour diagnosis across age groups: Cutaneous digital melanoma.


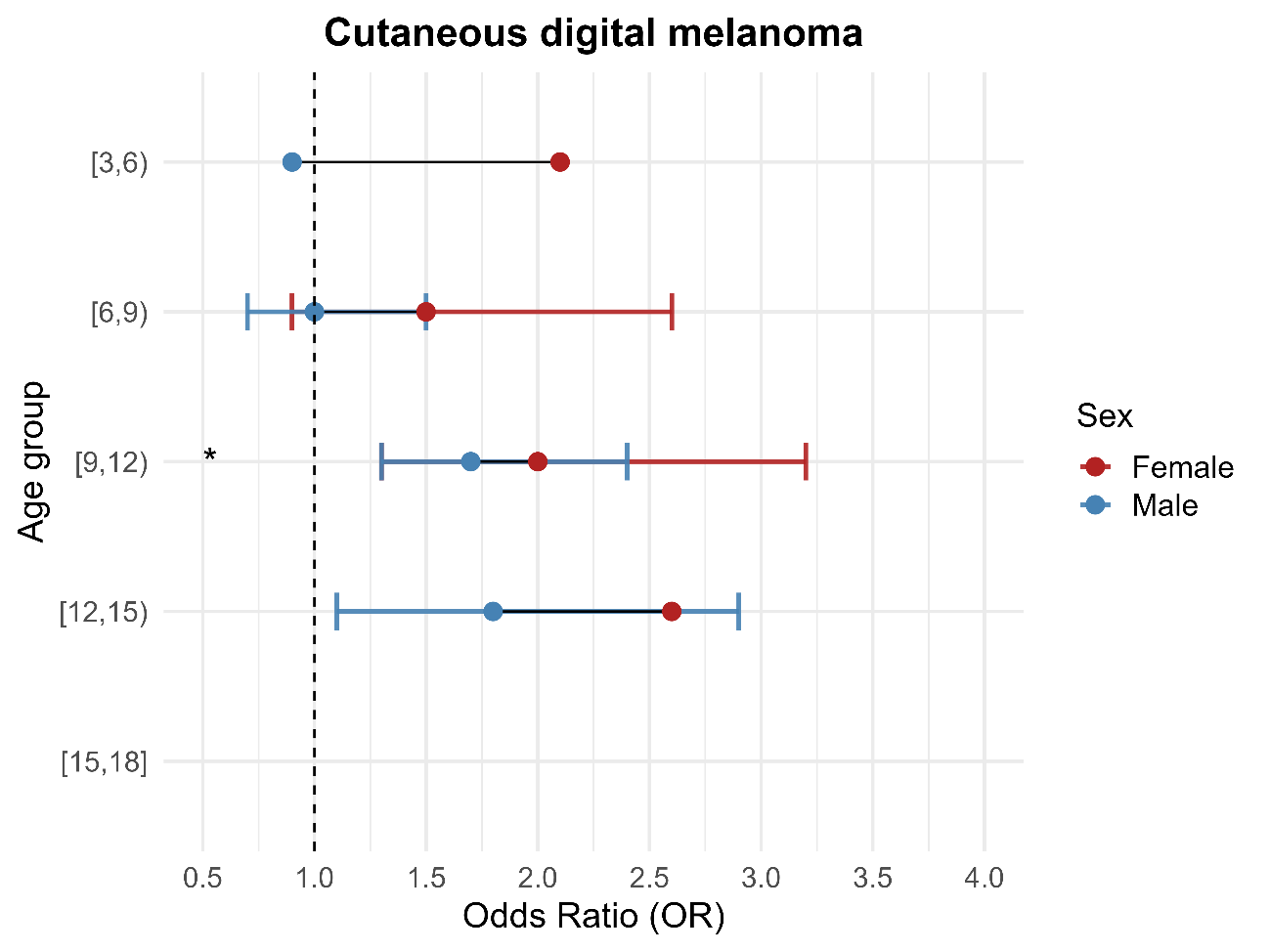


## Supplementary Figure 14: Sex-specific associations between neuter status and tumour diagnosis across age groups: Uveal melanoma.


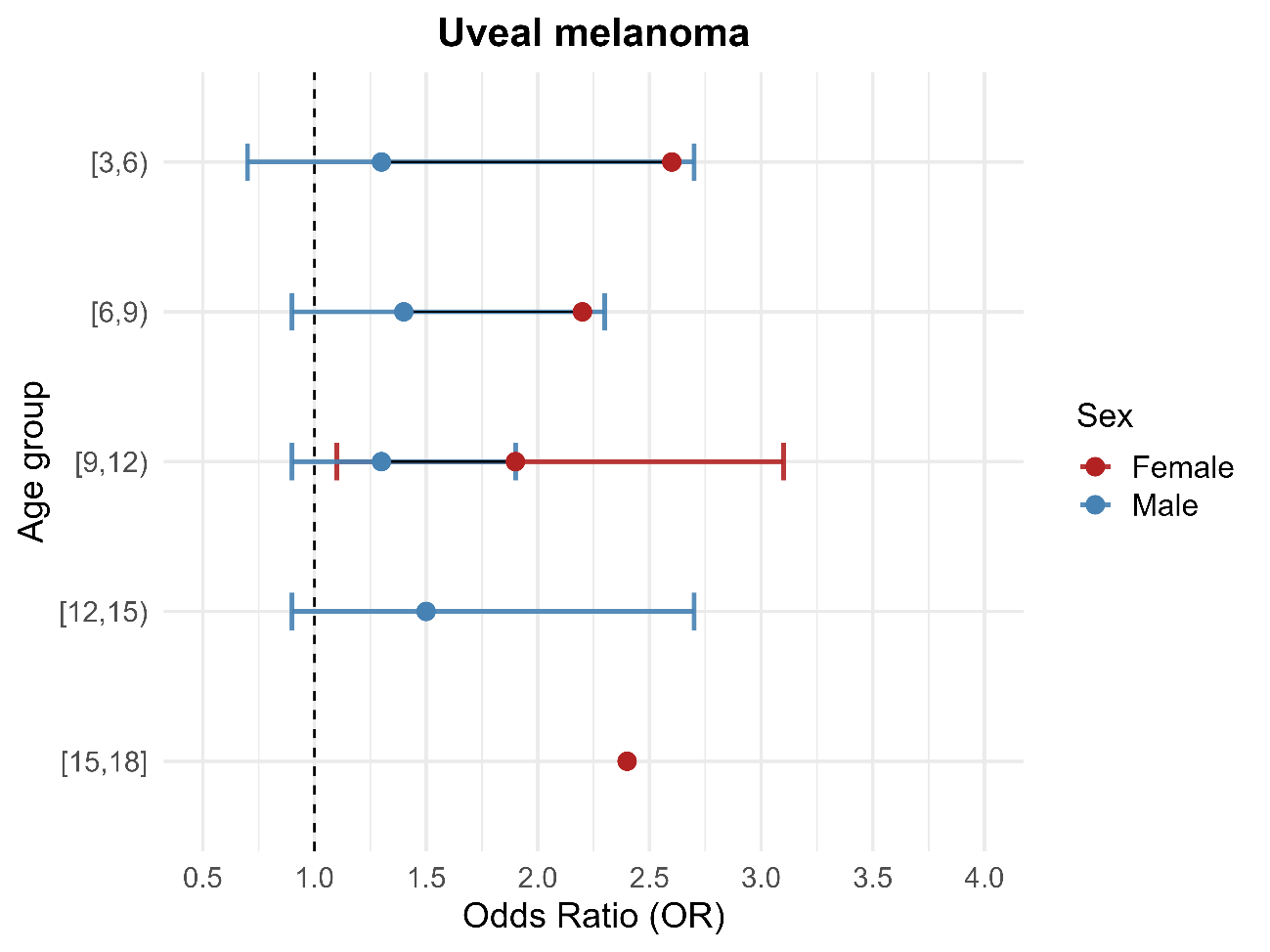


## Supplementary Figure 15: Sex-specific associations between neuter status and tumour diagnosis across age groups: Splenic haemangiosarcoma.


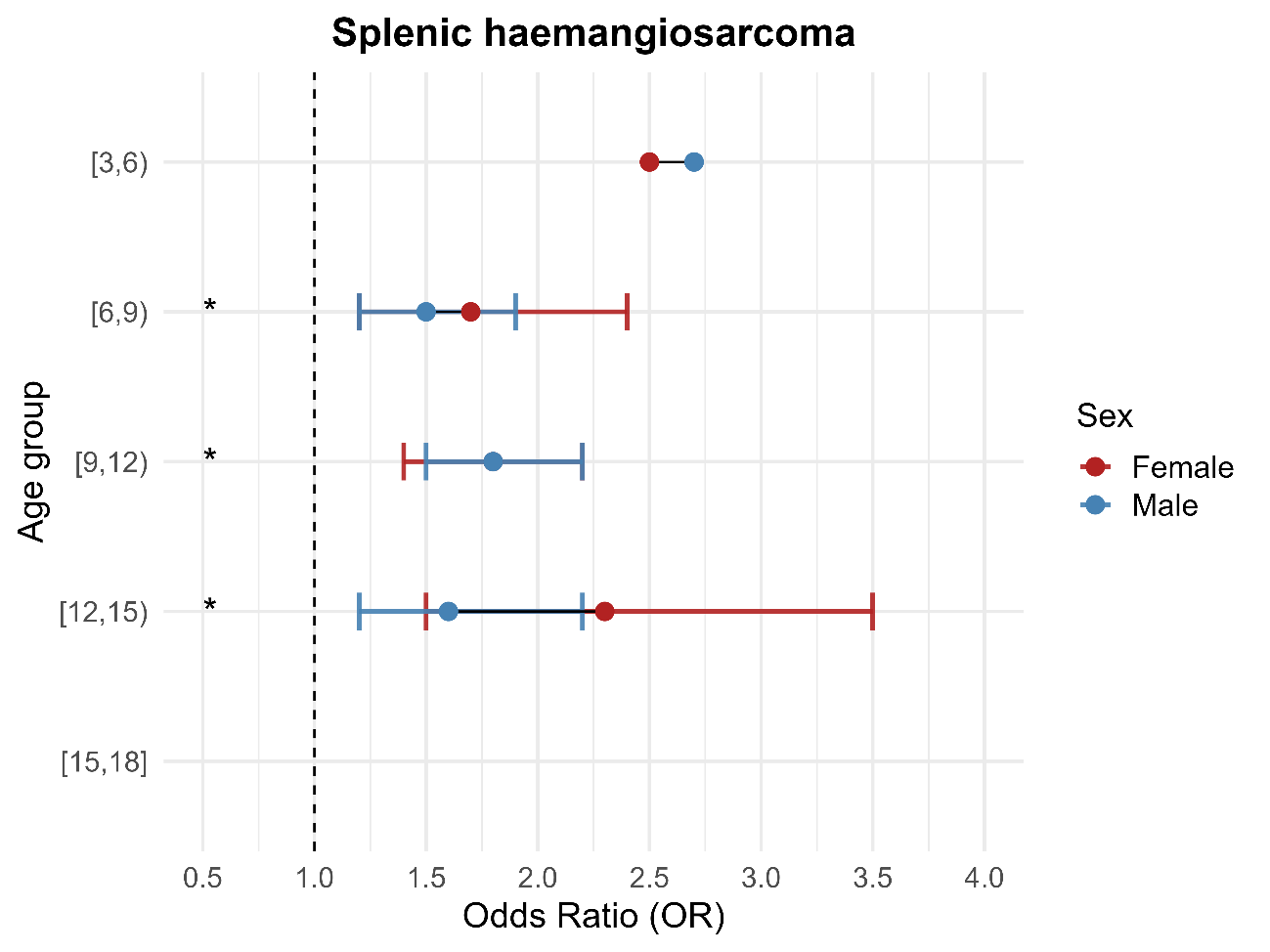


## Supplementary Figure 16: Sex-specific associations between neuter status and tumour diagnosis across age groups: Cutaneous haemangiosarcoma.


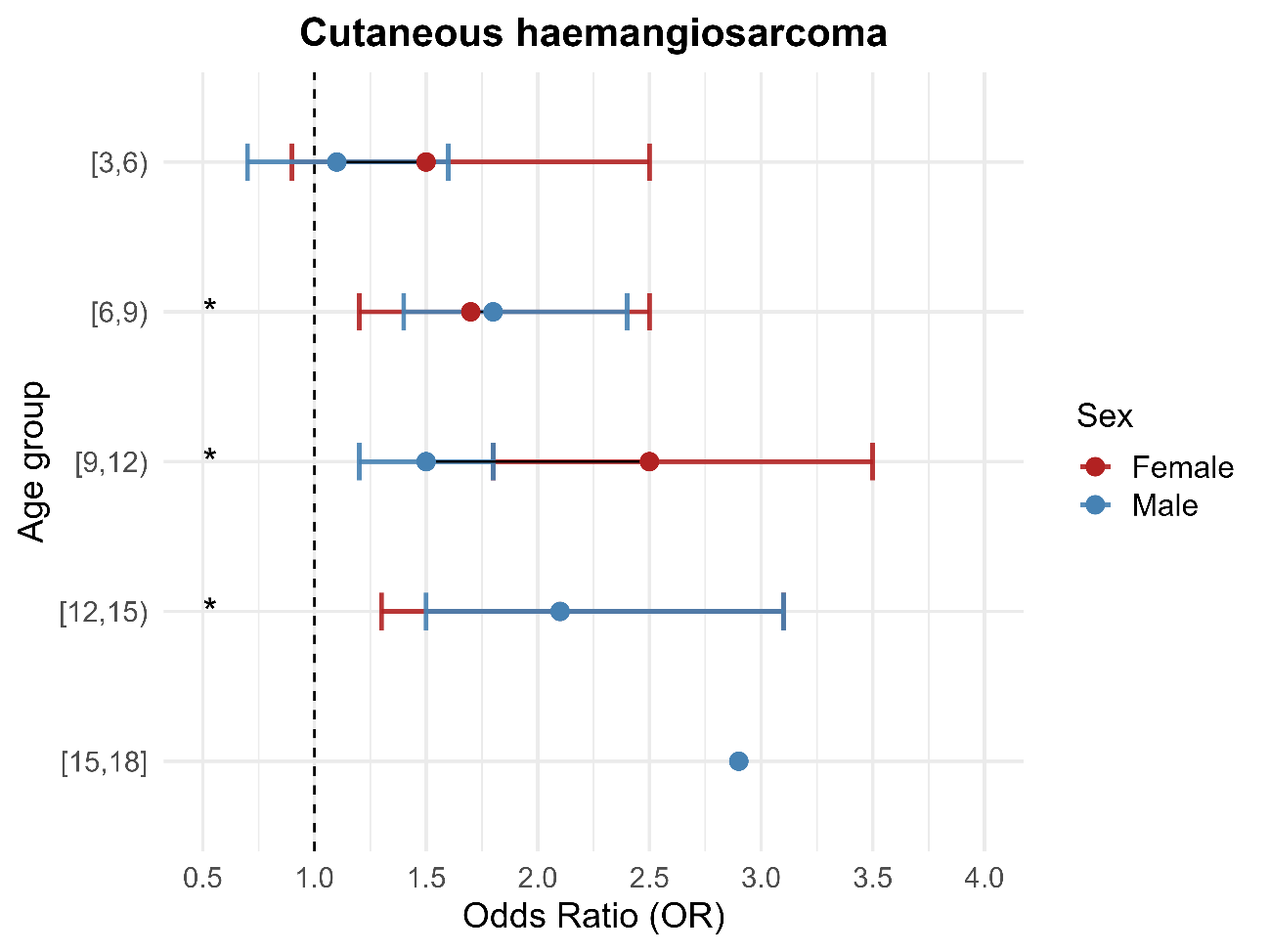


## Supplementary Figure 17: Sex-specific associations between neuter status and tumour diagnosis across age groups: Osteosarcoma.


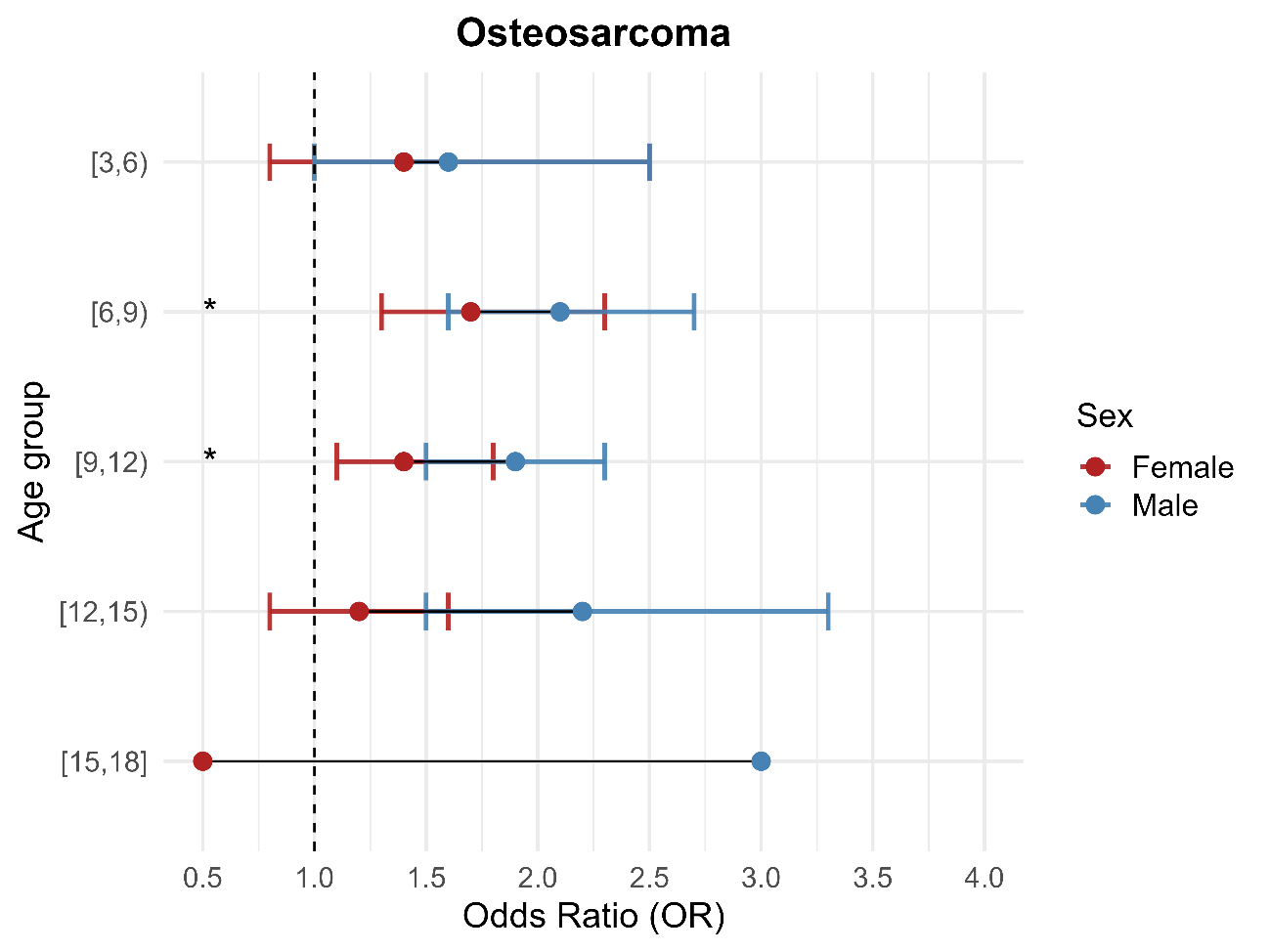


# Supplementary Tables (n = 20).

## Note to readers about the supplementary tables.

**The following conventions apply across all supplementary tables presented in this document:**

- **Overall Proportion*** refers to the proportion calculated using the total number of tumours, including those with missing or non-valid entries.
- **Valid Proportion*** refers to the proportion calculated excluding cases marked as NA or Not Otherwise Specified (NOS).
- **NOS (Not Otherwise Specified)** indicates cases where further subtyping was not provided in the original diagnostic report.
- **NA (Not Available)** indicates missing data in a given category or when a specific variable was not applicable or recorded.
- **Age intervals** are represented using standard mathematical interval notation:
  - Square brackets **[ ]** indicate the value is included in the interval.
  - Round brackets **( )** indicate the value is excluded.
  - For example, **[3,6)** means “greater than or equal to 3 and less than 6 years old.”

**Additional notes:**

- **Full data tables (e.g., Table 5):** These tables present odds ratios (ORs), confidence intervals, and p-values for the main logistic regression analysis comparing breeds, sexes, and age groups. Reference categories are explicitly stated in the Methods section.
- **Emmeans tables (e.g., Table 8):** These tables show post hoc pairwise comparisons between groups based on estimated marginal means (EMMs) from the logistic regression models.
  - **Contrast:** Pairwise group comparison.
  - **OR:** Estimated odds ratio for the contrast.
  - **SE:** Standard error of the estimate.
  - **Reference value:** Value of the odds ratio under the Reference value hypothesis (always 1, indicating no difference).
  - **Z ratio:** Standardized test statistic (estimate divided by SE).
  - **p-value:** Associated p-value for the test of the contrast.

**Abbreviations for sex and neuter status used in contrasts:**

- **FE** = Female entire
- **FN** = Female neutered
- **ME** = Male entire
- **MN** = Male neutered

**Extreme confidence intervals:** In rare cases where upper bounds of confidence intervals were extremely large (e.g., due to low sample size or separation), they were capped at a maximum display value of **2.3e+24** to maintain consistency and readability across tables.

These notes aim to facilitate interpretation and ensure consistent understanding of the supplementary tables.

## Supplementary Table 1: Mean number of diagnoses per dog per year for each tumour type, along with the 95% confidence interval (CI) for the mean.

| **Tumor type** | **Mean diagnoses per dog** | **Standard Deviation (SD)** | **Dogs (n)** | **Standard Error (SE)** | **CI lower bound** | **CI upper bound** |
| --- | --- | --- | --- | --- | --- | --- |
| Mast Cell Tumour | 1.102443 | 0.035 | 88,118 | 0 | 1.102 | 1.103 |
| Melanoma | 1.025884 | 0.011 | 16,104 | 0 | 1.026 | 1.026 |
| Haemangiosarcoma | 1.033523 | 0.016 | 10,868 | 0 | 1.033 | 1.034 |
| Osteosarcoma | 1.011756 | 0.011 | 4,599 | 0 | 1.011 | 1.012 |
| This table presents the mean number of diagnoses per affected dog for each tumor type over the study period, including measures of variability such as standard deviation (SD), standard error (SE), and 95% confidence intervals (CI). | | | | | | |

## Supplementary Table 2: Anatomical distribution of mast cell tumours

### Table 2.a: Distribution of mast cell tumours on the skin and on non-skin locations.

| **Location** | **Count (n)** | **Overall Proportion*** | **Valid Proportion*** |
| --- | --- | --- | --- |
| Skin | 86,087 | 88.0% | 91.0% |
| No skin | 8,485 | 8.7% | 9.0% |
| NA | 3,307 | 3.4% | - |
| Total | 97,879 | - | - |

### Table 2.b: Specific anatomical distribution of mast cell tumours

| **Location** | **Count (n)** | **Overall Proportion*** | **Valid Proportion*** |
| --- | --- | --- | --- |
| skin head and face | 15,653 | 16.0% | 16.7% |
| skin hindlimb | 13,886 | 14.2% | 14.8% |
| skin NOS* | 10,478 | 10.7% | 11.2% |
| skin thorax | 9,577 | 9.8% | 10.2% |
| skin trunk | 8,813 | 9.0% | 9.4% |
| skin forelimb | 7,494 | 7.7% | 8.0% |
| skin abdominal and inguinal region | 5,038 | 5.1% | 5.4% |
| NA* | 3,940 | 4.0% | - |
| skin ear | 3,593 | 3.7% | 3.8% |
| skin neck | 2,704 | 2.8% | 2.9% |
| skin testis, scrotum and penis | 2,444 | 2.5% | 2.6% |
| oral cavity | 1,831 | 1.9% | 1.9% |
| skin carpus and metacarpus | 1,709 | 1.7% | 1.8% |
| lymph node | 1,629 | 1.7% | 1.7% |
| skin anal and perianal region | 1,501 | 1.5% | 1.6% |
| skin pelvic region | 1,440 | 1.5% | 1.5% |
| skin tail | 1,155 | 1.2% | 1.2% |
| female reproductive system | 1,043 | 1.1% | 1.1% |
| eye and adnexa | 1,041 | 1.1% | 1.1% |
| trunk | 531 | 0.5% | 0.6% |
| thorax | 493 | 0.5% | 0.5% |
| skin tarsus and metatarsus | 306 | 0.3% | 0.3% |
| accessory and paranasal sinuses | 245 | 0.3% | 0.3% |
| skin paws | 215 | 0.2% | 0.2% |
| alimentary tract | 194 | 0.2% | 0.2% |
| mammary gland | 183 | 0.2% | 0.2% |
| nasal cavity | 145 | 0.1% | 0.2% |
| neck | 133 | 0.1% | 0.1% |
| liver and pancreas | 90 | 0.1% | 0.1% |
| skin limb | 81 | 0.1% | 0.1% |
| testis, scrotum and penis | 55 | 0.1% | 0.1% |
| pelvic region | 42 | 0.0% | 0.0% |
| spleen | 41 | 0.0% | 0.0% |
| respiratory tract | 39 | 0.0% | 0.0% |
| multiple lymph nodes | 21 | 0.0% | 0.0% |
| joint | 19 | 0.0% | 0.0% |
| nerve | 13 | 0.0% | 0.0% |
| tail | 12 | 0.0% | 0.0% |
| heart, mediastinum and pleura | 10 | 0.0% | 0.0% |
| kidney and urinary tract | 10 | 0.0% | 0.0% |
| salivary glands | 7 | 0.0% | 0.0% |
| gall and urinary bladder | 5 | 0.0% | 0.0% |
| retroperitoneum and peritoneum | 4 | 0.0% | 0.0% |
| thyroid gland | 4 | 0.0% | 0.0% |
| parathyroid gland | 3 | 0.0% | 0.0% |
| tarsus and metatarsus | 3 | 0.0% | 0.0% |
| brachial plexus | 2 | 0.0% | 0.0% |
| paws | 2 | 0.0% | 0.0% |
| brain | 1 | 0.0% | 0.0% |
| vertebral column | 1 | 0.0% | 0.0% |
| Total | 97,879 | - | - |
| Note: Overall Proportion* includes all cases, including those with NA values. Valid Proportion* includes only cases without NA values. NOS* stands for "Not otherwise specified". | | | |

## Supplementary Table 3: Distribution of mast cell tumours according to Patnaik 3-tier scheme

| **Grade** | **Count (n)** | **Overall Proportion*** | **Valid Proportion*** |
| --- | --- | --- | --- |
| Grade 1 | 4,317 | 4.4% | 6.1% |
| Grade 2 | 63,309 | 64.7% | 90.1% |
| Grade 3 | 2,650 | 2.7% | 3.8% |
| NA | 27,603 | 28.2% | - |
| Total | 97,879 | 100.0% | 100.0% |

## Supplementary Table 4: Distribution of Mast cell tumours according to Kiupel 2-tier scheme

| **Grade** | **Count (n)** | **Overall Proportion*** | **Valid Proportion*** |
| --- | --- | --- | --- |
| High-Grade | 8,298 | 8.5% | 15.5% |
| Low-Grade | 45,375 | 46.4% | 84.5% |
| NA | 44,206 | 45.2% | - |
| Total | 97,879 | 100.0% | 100.0% |

## Supplementary table 5: Full data table from the multivariable logistic regression analysis for mast cell tumours.

| **Effect** | **Value** | **OR** | **95% CI** | **p-value** | **n (%) (Controls)** | **n (%) (Cases)** |
| --- | --- | --- | --- | --- | --- | --- |
| Breed | Hungarian Vizsla | 2.6 | [2.2, 3.1] | <0.001 | 358 (0.4) | 310 (1.3) |
| Breed | Irish Terrier | 2.2 | [1.5, 3.2] | <0.001 | 72 (0.1) | 62 (0.3) |
| Breed | Pharaoh Hound | 2.2 | [0.5, 10.1] | 0.31 | 6 (0.0) | 5 (0.0) |
| Breed | Manchester Terrier | 2.1 | [1.2, 3.7] | 0.01 | 30 (0.0) | 21 (0.1) |
| Breed | Retriever (Labrador) | 2.1 | [2.0, 2.2] | <0.001 | 10847 (13.0) | 9131 (36.9) |
| Breed | Bernese Mountain Dog | 1.9 | [1.4, 2.7] | <0.001 | 116 (0.1) | 77 (0.3) |
| Breed | Rhodesian Ridgeback | 1.9 | [1.5, 2.4] | <0.001 | 218 (0.3) | 153 (0.6) |
| Breed | American Bulldog | 1.5 | [1.0, 2.1] | 0.05 | 105 (0.1) | 42 (0.2) |
| Breed | English Setter | 1.5 | [1.0, 2.1] | 0.04 | 102 (0.1) | 54 (0.2) |
| Breed | Italian Greyhound | 1.5 | [0.8, 2.9] | 0.18 | 38 (0.0) | 16 (0.1) |
| Breed | Retriever (Nova Scotia Duck Tolling) | 1.3 | [0.6, 2.8] | 0.45 | 23 (0.0) | 13 (0.1) |
| Breed | Schnauzer | 1.3 | [1.0, 1.7] | 0.08 | 198 (0.2) | 99 (0.4) |
| Breed | Dogue De Bordeaux | 1.2 | [0.8, 1.7] | 0.43 | 128 (0.2) | 49 (0.2) |
| Breed | Foxhound | 1.2 | [0.4, 3.4] | 0.71 | 17 (0.0) | 5 (0.0) |
| Breed | English Pointer | 1.1 | [0.9, 1.5] | 0.33 | 237 (0.3) | 97 (0.4) |
| Breed | Great Dane | 1.1 | [0.8, 1.5] | 0.54 | 154 (0.2) | 60 (0.2) |
| Breed | Miniature Schnauzer | 1.1 | [1.0, 1.3] | 0.08 | 867 (1.0) | 375 (1.5) |
| Breed | Crossbreed | 1 |  |  | 17598 (21.1) | 6696 (27.1) |
| Breed | Bolognese | 1 | [0.4, 2.8] | 0.93 | 16 (0.0) | 6 (0.0) |
| Breed | Collie (Rough) | 1 | [0.8, 1.2] | 0.87 | 274 (0.3) | 102 (0.4) |
| Breed | Maltese | 1 | [0.7, 1.3] | 0.78 | 173 (0.2) | 52 (0.2) |
| Breed | Dobermann | 0.8 | [0.6, 1.0] | 0.06 | 283 (0.3) | 78 (0.3) |
| Breed | Japanese Shiba Inu | 0.8 | [0.4, 1.9] | 0.67 | 32 (0.0) | 9 (0.0) |
| Breed | Alaskan Malamute | 0.7 | [0.5, 1.0] | 0.07 | 144 (0.2) | 32 (0.1) |
| Breed | Basset Hound | 0.7 | [0.5, 1.0] | 0.02 | 241 (0.3) | 65 (0.3) |
| Breed | German Shorthaired Pointer | 0.7 | [0.5, 0.9] | 0.01 | 304 (0.4) | 84 (0.3) |
| Breed | Irish Wolfhound | 0.7 | [0.2, 1.8] | 0.41 | 34 (0.0) | 5 (0.0) |
| Breed | Italian Spinone | 0.7 | [0.4, 1.1] | 0.15 | 87 (0.1) | 20 (0.1) |
| Breed | Jack Russell Terrier | 0.7 | [0.7, 0.8] | <0.001 | 6180 (7.4) | 1755 (7.1) |
| Breed | Retriever (Flat Coated) | 0.7 | [0.6, 0.9] | 0.02 | 301 (0.4) | 77 (0.3) |
| Breed | Rottweiler | 0.7 | [0.6, 0.9] | <0.001 | 580 (0.7) | 167 (0.7) |
| Breed | Shih Tzu | 0.7 | [0.6, 0.7] | <0.001 | 1662 (2.0) | 402 (1.6) |
| Breed | Siberian Husky | 0.7 | [0.6, 0.9] | 0.01 | 441 (0.5) | 114 (0.5) |
| Breed | Soft Coated Wheaten Terrier | 0.7 | [0.4, 1.2] | 0.19 | 59 (0.1) | 14 (0.1) |
| Breed | Australian Cattle Dog | 0.6 | [0.2, 1.7] | 0.36 | 22 (0.0) | 5 (0.0) |
| Breed | Basset Griffon Vendeen (Petit) | 0.6 | [0.2, 1.8] | 0.37 | 18 (0.0) | 5 (0.0) |
| Breed | Bichon Frise | 0.6 | [0.5, 0.6] | <0.001 | 1029 (1.2) | 231 (0.9) |
| Breed | Briard | 0.6 | [0.3, 1.5] | 0.30 | 31 (0.0) | 7 (0.0) |
| Breed | Labradoodle | 0.6 | [0.5, 0.7] | <0.001 | 686 (0.8) | 143 (0.6) |
| Breed | Shetland Sheepdog | 0.6 | [0.5, 0.8] | 0.01 | 237 (0.3) | 59 (0.2) |
| Breed | Terrier (Generic) | 0.6 | [0.5, 0.7] | <0.001 | 809 (1.0) | 186 (0.8) |
| Breed | Tibetan Terrier | 0.6 | [0.4, 0.8] | <0.001 | 255 (0.3) | 61 (0.2) |
| Breed | Chihuahua | 0.5 | [0.4, 0.6] | <0.001 | 1085 (1.3) | 167 (0.7) |
| Breed | Dachshund (Standard) | 0.5 | [0.4, 0.6] | <0.001 | 727 (0.9) | 109 (0.4) |
| Breed | Giant Schnauzer | 0.5 | [0.2, 1.0] | 0.06 | 42 (0.1) | 8 (0.0) |
| Breed | Griffon Bruxellois | 0.5 | [0.2, 1.4] | 0.19 | 26 (0.0) | 5 (0.0) |
| Breed | Kerry Blue Terrier | 0.5 | [0.2, 1.2] | 0.12 | 33 (0.0) | 8 (0.0) |
| Breed | Leonberger | 0.5 | [0.2, 1.1] | 0.08 | 47 (0.1) | 9 (0.0) |
| Breed | Spaniel (English Springer) | 0.5 | [0.4, 0.5] | <0.001 | 3896 (4.7) | 734 (3.0) |
| Breed | Belgian Shepherd Dog | 0.4 | [0.2, 0.8] | 0.01 | 66 (0.1) | 9 (0.0) |
| Breed | Cairn Terrier | 0.4 | [0.3, 0.5] | <0.001 | 516 (0.6) | 75 (0.3) |
| Breed | Lhasa Apso | 0.4 | [0.4, 0.5] | <0.001 | 1102 (1.3) | 191 (0.8) |
| Breed | Lurcher | 0.4 | [0.3, 0.5] | <0.001 | 1205 (1.4) | 188 (0.8) |
| Breed | Norfolk Terrier | 0.4 | [0.2, 0.6] | <0.001 | 185 (0.2) | 27 (0.1) |
| Breed | Parson Russell Terrier | 0.4 | [0.2, 0.5] | <0.001 | 299 (0.4) | 37 (0.1) |
| Breed | Patterdale Terrier | 0.4 | [0.3, 0.6] | <0.001 | 583 (0.7) | 91 (0.4) |
| Breed | Saluki | 0.4 | [0.2, 0.9] | 0.02 | 55 (0.1) | 7 (0.0) |
| Breed | Welsh Corgi | 0.4 | [0.2, 0.8] | 0.02 | 57 (0.1) | 8 (0.0) |
| Breed | Yorkshire Terrier | 0.4 | [0.3, 0.4] | <0.001 | 2166 (2.6) | 311 (1.3) |
| Breed | Bedlington Terrier | 0.3 | [0.1, 0.4] | <0.001 | 173 (0.2) | 17 (0.1) |
| Breed | Chinese Crested | 0.3 | [0.1, 0.6] | 0.01 | 88 (0.1) | 10 (0.0) |
| Breed | Chow Chow | 0.3 | [0.1, 0.7] | 0.01 | 52 (0.1) | 5 (0.0) |
| Breed | Cockapoo | 0.3 | [0.2, 0.4] | <0.001 | 881 (1.1) | 71 (0.3) |
| Breed | Gordon Setter | 0.3 | [0.1, 0.6] | 0.01 | 73 (0.1) | 7 (0.0) |
| Breed | Greyhound | 0.3 | [0.2, 0.3] | <0.001 | 1252 (1.5) | 140 (0.6) |
| Breed | Newfoundland | 0.3 | [0.1, 0.5] | <0.001 | 143 (0.2) | 14 (0.1) |
| Breed | Norwich Terrier | 0.3 | [0.1, 0.9] | 0.04 | 32 (0.0) | 5 (0.0) |
| Breed | Papillon | 0.3 | [0.2, 0.6] | <0.001 | 150 (0.2) | 18 (0.1) |
| Breed | Poodle (Generic) | 0.3 | [0.2, 0.5] | <0.001 | 264 (0.3) | 34 (0.1) |
| Breed | Whippet | 0.3 | [0.2, 0.4] | <0.001 | 770 (0.9) | 80 (0.3) |
| Breed | Akita | 0.2 | [0.1, 0.4] | <0.001 | 143 (0.2) | 8 (0.0) |
| Breed | Bearded Collie | 0.2 | [0.1, 0.3] | <0.001 | 271 (0.3) | 19 (0.1) |
| Breed | Border Collie | 0.2 | [0.2, 0.2] | <0.001 | 4543 (5.4) | 299 (1.2) |
| Breed | Dachshund (Miniature) | 0.2 | [0.2, 0.3] | <0.001 | 486 (0.6) | 45 (0.2) |
| Breed | Fox Terrier | 0.2 | [0.2, 0.4] | <0.001 | 194 (0.2) | 19 (0.1) |
| Breed | German Shepherd Dog | 0.2 | [0.1, 0.2] | <0.001 | 2154 (2.6) | 129 (0.5) |
| Breed | Lakeland Terrier | 0.2 | [0.1, 0.4] | <0.001 | 223 (0.3) | 18 (0.1) |
| Breed | Old English Sheepdog | 0.2 | [0.1, 0.4] | <0.001 | 89 (0.1) | 5 (0.0) |
| Breed | Pomeranian | 0.2 | [0.1, 0.4] | <0.001 | 267 (0.3) | 18 (0.1) |
| Breed | Scottish Terrier | 0.2 | [0.1, 0.3] | <0.001 | 220 (0.3) | 19 (0.1) |
| Breed | Spaniel (Cocker) | 0.2 | [0.2, 0.2] | <0.001 | 5384 (6.5) | 387 (1.6) |
| Breed | Spaniel (Welsh Springer) | 0.2 | [0.1, 0.4] | <0.001 | 136 (0.2) | 11 (0.0) |
| Breed | Unknown | 0.2 | [0.1, 0.4] | <0.001 | 201 (0.2) | 15 (0.1) |
| Breed | West Highland White Terrier | 0.2 | [0.2, 0.2] | <0.001 | 2646 (3.2) | 239 (1.0) |
| Breed | Border Terrier | 0.1 | [0.1, 0.1] | <0.001 | 2030 (2.4) | 82 (0.3) |
| Breed | Dalmatian | 0.1 | [0.1, 0.2] | <0.001 | 397 (0.5) | 23 (0.1) |
| Breed | Irish Setter | 0.1 | [0.1, 0.2] | <0.001 | 223 (0.3) | 9 (0.0) |
| Breed | Poodle (Standard) | 0.1 | [0.1, 0.2] | <0.001 | 429 (0.5) | 24 (0.1) |
| Breed | Poodle (Toy) | 0.1 | [0.0, 0.2] | <0.001 | 291 (0.3) | 7 (0.0) |
| Breed | Sprocker | 0.1 | [0.1, 0.2] | <0.001 | 292 (0.4) | 13 (0.1) |
| Breed | Cavalier King Charles Spaniel | 0 | [0.0, 0.0] | <0.001 | 1766 (2.1) | 17 (0.1) |
| Sex | Female neutered | 1 |  |  | 28367 (34.0) | 9880 (39.9) |
| Sex | Female entire | 0.6 | [0.5, 0.6] | <0.001 | 14238 (17.1) | 2528 (10.2) |
| Sex | Male neutered | 0.9 | [0.9, 1.0] | 0.11 | 25372 (30.4) | 7143 (28.9) |
| Sex | Male entire | 0.7 | [0.6, 0.8] | <0.001 | 15388 (18.5) | 2936 (11.9) |
| Age | [3,6) | 1 |  |  | 24089 (28.9) | 4188 (16.9) |
| Age | [6,9) | 2.5 | [2.3, 2.6] | <0.001 | 21866 (26.2) | 9191 (37.2) |
| Age | [9,12) | 3.1 | [2.9, 3.3] | <0.001 | 17304 (20.8) | 8557 (34.6) |
| Age | [12,15) | 1.6 | [1.5, 1.7] | <0.001 | 9580 (11.5) | 2433 (9.8) |
| Age | [15,18] | 0.5 | [0.4, 0.7] | <0.001 | 1994 (2.4) | 167 (0.7) |

## Supplementary table 6: Full results from the univariable logistic regression analysis for mast cell tumours. Breeds with significantly increased odds of mast cell tumour diagnosis compared to Crossbreeds, based on a model restricted to dogs diagnosed with tumours.

| **Breed** | **OR** | **95% CI** | | **p-value** | | **n (%) (Controls)** | | **n (%) (Cases)** | |
| --- | --- | --- | --- | --- | --- | --- | --- | --- | --- |
| Boston Terrier | 6.8 | [6.0, 7.6] | <0.001 | | 651 (0.1) | | 589 (0.9) | |  |
| Retriever (Curly Coated) | 5.9 | [4.1, 8.6] | <0.001 | | 62 (0.0) | | 49 (0.1) | |  |
| Hungarian Wirehaired Vizsla | 5.2 | [2.0, 13.7] | <0.001 | | 10 (0.0) | | 7 (0.0) | |  |
| Shar Pei | 3.8 | [3.4, 4.3] | <0.001 | | 883 (0.2) | | 452 (0.7) | |  |
| Boxer | 3.7 | [3.6, 3.8] | <0.001 | | 13746 (3.1) | | 6834 (10.0) | |  |
| Staffordshire Bull Terrier | 3.6 | [3.5, 3.7] | <0.001 | | 23617 (5.4) | | 11301 (16.6) | |  |
| American Bulldog | 3.5 | [2.5, 4.8] | <0.001 | | 108 (0.0) | | 50 (0.1) | |  |
| Pit Bull Terrier | 3.3 | [1.3, 7.9] | 0.009 | | 16 (0.0) | | 7 (0.0) | |  |
| Spaniel (Sussex) | 3.2 | [2.1, 4.8] | <0.001 | | 77 (0.0) | | 33 (0.0) | |  |
| Weimaraner | 3 | [2.8, 3.3] | <0.001 | | 2352 (0.5) | | 954 (1.4) | |  |
| Retriever (Nova Scotia Duck Tolling) | 2.9 | [1.6, 5.4] | <0.001 | | 36 (0.0) | | 14 (0.0) | |  |
| American Staffordshire Terrier | 2.8 | [2.0, 3.7] | <0.001 | | 152 (0.0) | | 56 (0.1) | |  |
| Pharaoh Hound | 2.5 | [1.1, 5.9] | 0.037 | | 21 (0.0) | | 7 (0.0) | |  |
| French Bulldog | 2.4 | [2.2, 2.5] | <0.001 | | 4749 (1.1) | | 1512 (2.2) | |  |
| Retriever (Golden) | 2.3 | [2.2, 2.4] | <0.001 | | 11952 (2.7) | | 3670 (5.4) | |  |
| Spaniel (Irish Water) | 2.3 | [1.6, 3.1] | <0.001 | | 152 (0.0) | | 46 (0.1) | |  |
| Bullmastiff | 2.2 | [1.9, 2.5] | <0.001 | | 1206 (0.3) | | 353 (0.5) | |  |
| Retriever (Labrador) | 2.1 | [2.1, 2.2] | <0.001 | | 52471 (11.9) | | 15062 (22.1) | |  |
| Retriever (Generic) | 2.1 | [1.9, 2.3] | <0.001 | | 2161 (0.5) | | 610 (0.9) | |  |
| Canaan Dog | 1.9 | [0.8, 4.6] | 0.172 | | 24 (0.0) | | 6 (0.0) | |  |
| Manchester Terrier | 1.9 | [1.3, 2.7] | <0.001 | | 140 (0.0) | | 35 (0.1) | |  |
| Mastiff | 1.7 | [1.4, 2.0] | <0.001 | | 750 (0.2) | | 169 (0.2) | |  |
| Bulldog | 1.6 | [1.5, 1.7] | <0.001 | | 4027 (0.9) | | 845 (1.2) | |  |
| Bull Terrier | 1.6 | [1.4, 1.7] | <0.001 | | 1941 (0.4) | | 404 (0.6) | |  |
| Irish Terrier | 1.4 | [1.1, 1.8] | 0.004 | | 424 (0.1) | | 81 (0.1) | |  |
| New Zealand Huntaway | 1.4 | [0.5, 3.6] | 0.506 | | 27 (0.0) | | 5 (0.0) | |  |
| Italian Greyhound | 1.4 | [0.9, 2.1] | 0.165 | | 138 (0.0) | | 25 (0.0) | |  |
| Bernese Mountain Dog | 1.3 | [1.1, 1.6] | 0.006 | | 790 (0.2) | | 137 (0.2) | |  |
| Goldendoodle | 1.3 | [0.5, 3.3] | 0.602 | | 29 (0.0) | | 5 (0.0) | |  |
| Beagle | 1.2 | [1.1, 1.3] | <0.001 | | 5654 (1.3) | | 927 (1.4) | |  |
| Unknown | 1.2 | [0.7, 2.0] | 0.431 | | 110 (0.0) | | 18 (0.0) | |  |
| Hungarian Vizsla | 1.2 | [1.1, 1.3] | <0.001 | | 2778 (0.6) | | 447 (0.7) | |  |
| Maltese | 1.2 | [1.0, 1.5] | 0.120 | | 638 (0.1) | | 101 (0.1) | |  |
| Jack Russell Terrier | 1.1 | [1.1, 1.2] | <0.001 | | 18683 (4.2) | | 2795 (4.1) | |  |
| Norwich Terrier | 1.1 | [0.5, 2.1] | 0.857 | | 63 (0.0) | | 9 (0.0) | |  |
| Parson Russell Terrier | 1 | [0.8, 1.4] | 0.792 | | 388 (0.1) | | 54 (0.1) | |  |
| Great Dane | 1 | [0.8, 1.3] | 0.774 | | 724 (0.2) | | 100 (0.1) | |  |
| Norfolk Terrier | 1 | [0.7, 1.4] | 0.971 | | 334 (0.1) | | 45 (0.1) | |  |
| Crossbreed | 1 |  |  | | 76258 (17.3) | | 10214 (15.0) | |  |
| Terrier (Generic) | 1 | [0.8, 1.1] | 0.538 | | 2213 (0.5) | | 285 (0.4) | |  |
| Affenpinscher | 0.9 | [0.4, 2.2] | 0.873 | | 48 (0.0) | | 6 (0.0) | |  |
| Papillon | 0.9 | [0.6, 1.3] | 0.679 | | 266 (0.1) | | 33 (0.0) | |  |
| Miniature Schnauzer | 0.9 | [0.8, 1.0] | 0.094 | | 4423 (1.0) | | 548 (0.8) | |  |
| Rhodesian Ridgeback | 0.9 | [0.8, 1.0] | 0.208 | | 2033 (0.5) | | 250 (0.4) | |  |
| Shetland Sheepdog | 0.9 | [0.7, 1.1] | 0.300 | | 731 (0.2) | | 87 (0.1) | |  |
| Labradoodle | 0.9 | [0.8, 1.0] | 0.162 | | 1328 (0.3) | | 158 (0.2) | |  |
| Griffon Bruxellois | 0.8 | [0.3, 2.1] | 0.692 | | 45 (0.0) | | 5 (0.0) | |  |
| English Setter | 0.8 | [0.6, 1.0] | 0.062 | | 703 (0.2) | | 75 (0.1) | |  |
| Lucas Terrier | 0.8 | [0.3, 2.0] | 0.624 | | 47 (0.0) | | 5 (0.0) | |  |
| Foxhound | 0.7 | [0.3, 1.9] | 0.533 | | 50 (0.0) | | 5 (0.0) | |  |
| Patterdale Terrier | 0.7 | [0.6, 0.9] | <0.001 | | 1442 (0.3) | | 138 (0.2) | |  |
| Basset Griffon Vendeen (Petit) | 0.7 | [0.3, 1.5] | 0.357 | | 84 (0.0) | | 8 (0.0) | |  |
| English Pointer | 0.7 | [0.6, 0.8] | <0.001 | | 1457 (0.3) | | 129 (0.2) | |  |
| Bouvier Des Flandres | 0.7 | [0.3, 1.4] | 0.254 | | 91 (0.0) | | 8 (0.0) | |  |
| Lhasa Apso | 0.6 | [0.6, 0.7] | <0.001 | | 3378 (0.8) | | 294 (0.4) | |  |
| Chihuahua | 0.6 | [0.6, 0.7] | <0.001 | | 2405 (0.5) | | 208 (0.3) | |  |
| German Shorthaired Pointer | 0.6 | [0.5, 0.8] | <0.001 | | 1212 (0.3) | | 103 (0.2) | |  |
| Dogue De Bordeaux | 0.6 | [0.5, 0.8] | <0.001 | | 1067 (0.2) | | 90 (0.1) | |  |
| Schnauzer | 0.6 | [0.5, 0.7] | <0.001 | | 1739 (0.4) | | 131 (0.2) | |  |
| Spaniel (English Springer) | 0.5 | [0.5, 0.6] | <0.001 | | 17147 (3.9) | | 1246 (1.8) | |  |
| Cairn Terrier | 0.5 | [0.4, 0.6] | <0.001 | | 2012 (0.5) | | 143 (0.2) | |  |
| Japanese Shiba Inu | 0.5 | [0.3, 1.0] | 0.052 | | 141 (0.0) | | 10 (0.0) | |  |
| Rottweiler | 0.5 | [0.4, 0.6] | <0.001 | | 5029 (1.1) | | 338 (0.5) | |  |
| Shih Tzu | 0.5 | [0.5, 0.5] | <0.001 | | 8138 (1.8) | | 546 (0.8) | |  |
| Basset Hound | 0.5 | [0.4, 0.6] | <0.001 | | 1601 (0.4) | | 107 (0.2) | |  |
| Sprocker | 0.5 | [0.3, 0.9] | 0.015 | | 195 (0.0) | | 13 (0.0) | |  |
| Yorkshire Terrier | 0.5 | [0.5, 0.5] | <0.001 | | 7774 (1.8) | | 516 (0.8) | |  |
| Australian Cattle Dog | 0.5 | [0.2, 1.0] | 0.053 | | 111 (0.0) | | 7 (0.0) | |  |
| Leonberger | 0.5 | [0.3, 0.7] | <0.001 | | 325 (0.1) | | 20 (0.0) | |  |
| German Spitz | 0.4 | [0.2, 1.0] | 0.038 | | 117 (0.0) | | 7 (0.0) | |  |
| Lurcher | 0.4 | [0.4, 0.5] | <0.001 | | 4587 (1.0) | | 273 (0.4) | |  |
| Chinese Crested | 0.4 | [0.3, 0.8] | 0.004 | | 221 (0.1) | | 13 (0.0) | |  |
| Spaniel (Generic) | 0.4 | [0.4, 0.5] | <0.001 | | 3118 (0.7) | | 182 (0.3) | |  |
| Briard | 0.4 | [0.2, 0.7] | 0.002 | | 245 (0.1) | | 14 (0.0) | |  |
| Italian Spinone | 0.4 | [0.3, 0.6] | <0.001 | | 599 (0.1) | | 34 (0.0) | |  |
| Spaniel (Welsh Springer) | 0.4 | [0.3, 0.7] | <0.001 | | 372 (0.1) | | 21 (0.0) | |  |
| Greyhound | 0.4 | [0.4, 0.5] | <0.001 | | 4151 (0.9) | | 228 (0.3) | |  |
| Dachshund (Standard) | 0.4 | [0.3, 0.5] | <0.001 | | 3203 (0.7) | | 172 (0.3) | |  |
| Tibetan Terrier | 0.4 | [0.3, 0.5] | <0.001 | | 1767 (0.4) | | 93 (0.1) | |  |
| Collie (Rough) | 0.4 | [0.3, 0.5] | <0.001 | | 3885 (0.9) | | 204 (0.3) | |  |
| Siberian Husky | 0.4 | [0.3, 0.4] | <0.001 | | 3083 (0.7) | | 155 (0.2) | |  |
| Dobermann | 0.4 | [0.3, 0.4] | <0.001 | | 2669 (0.6) | | 134 (0.2) | |  |
| Norwegian Elkhound | 0.4 | [0.2, 0.8] | 0.018 | | 121 (0.0) | | 6 (0.0) | |  |
| Belgian Shepherd Dog | 0.4 | [0.2, 0.6] | <0.001 | | 349 (0.1) | | 17 (0.0) | |  |
| Spaniel (American Cocker) | 0.4 | [0.2, 0.7] | <0.001 | | 227 (0.1) | | 11 (0.0) | |  |
| Bichon Frise | 0.3 | [0.3, 0.4] | <0.001 | | 6760 (1.5) | | 316 (0.5) | |  |
| Portuguese Water Dog | 0.3 | [0.1, 0.8] | 0.012 | | 117 (0.0) | | 5 (0.0) | |  |
| Border Collie | 0.3 | [0.3, 0.3] | <0.001 | | 11793 (2.7) | | 495 (0.7) | |  |
| Tibetan Spaniel | 0.3 | [0.2, 0.6] | 0.001 | | 195 (0.0) | | 8 (0.0) | |  |
| Dachshund (Miniature) | 0.3 | [0.2, 0.4] | <0.001 | | 1401 (0.3) | | 57 (0.1) | |  |
| Saluki | 0.3 | [0.2, 0.5] | <0.001 | | 425 (0.1) | | 17 (0.0) | |  |
| German Wirehaired Pointer | 0.3 | [0.1, 0.7] | 0.003 | | 153 (0.0) | | 6 (0.0) | |  |
| Lakeland Terrier | 0.3 | [0.2, 0.4] | <0.001 | | 525 (0.1) | | 20 (0.0) | |  |
| West Highland White Terrier | 0.3 | [0.3, 0.3] | <0.001 | | 11211 (2.5) | | 425 (0.6) | |  |
| Japanese Spitz | 0.3 | [0.1, 0.5] | <0.001 | | 268 (0.1) | | 10 (0.0) | |  |
| Whippet | 0.3 | [0.2, 0.3] | <0.001 | | 2882 (0.7) | | 107 (0.2) | |  |
| Pekingese | 0.3 | [0.1, 0.5] | <0.001 | | 220 (0.0) | | 8 (0.0) | |  |
| Alaskan Malamute | 0.3 | [0.2, 0.4] | <0.001 | | 1046 (0.2) | | 38 (0.1) | |  |
| Bedlington Terrier | 0.3 | [0.2, 0.4] | <0.001 | | 732 (0.2) | | 26 (0.0) | |  |
| Irish Wolfhound | 0.3 | [0.1, 0.6] | 0.001 | | 170 (0.0) | | 6 (0.0) | |  |
| Retriever (Flat Coated) | 0.3 | [0.2, 0.3] | <0.001 | | 3172 (0.7) | | 111 (0.2) | |  |
| Fox Terrier | 0.3 | [0.2, 0.4] | <0.001 | | 806 (0.2) | | 27 (0.0) | |  |
| Bearded Collie | 0.2 | [0.2, 0.4] | <0.001 | | 896 (0.2) | | 30 (0.0) | |  |
| Pomeranian | 0.2 | [0.2, 0.4] | <0.001 | | 773 (0.2) | | 25 (0.0) | |  |
| Giant Schnauzer | 0.2 | [0.1, 0.4] | <0.001 | | 395 (0.1) | | 12 (0.0) | |  |
| Newfoundland | 0.2 | [0.1, 0.4] | <0.001 | | 664 (0.2) | | 20 (0.0) | |  |
| Poodle (Standard) | 0.2 | [0.2, 0.3] | <0.001 | | 1341 (0.3) | | 39 (0.1) | |  |
| Cockapoo | 0.2 | [0.2, 0.3] | <0.001 | | 2579 (0.6) | | 75 (0.1) | |  |
| St. Bernard | 0.2 | [0.1, 0.5] | <0.001 | | 212 (0.0) | | 6 (0.0) | |  |
| Poodle (Generic) | 0.2 | [0.2, 0.3] | <0.001 | | 2010 (0.5) | | 56 (0.1) | |  |
| Cavachon | 0.2 | [0.1, 0.5] | <0.001 | | 180 (0.0) | | 5 (0.0) | |  |
| Scottish Terrier | 0.2 | [0.2, 0.3] | <0.001 | | 1649 (0.4) | | 45 (0.1) | |  |
| Chow Chow | 0.2 | [0.1, 0.5] | <0.001 | | 223 (0.1) | | 6 (0.0) | |  |
| Welsh Corgi | 0.2 | [0.1, 0.4] | <0.001 | | 341 (0.1) | | 9 (0.0) | |  |
| Poodle (Toy) | 0.2 | [0.1, 0.3] | <0.001 | | 781 (0.2) | | 20 (0.0) | |  |
| Gordon Setter | 0.2 | [0.1, 0.3] | <0.001 | | 491 (0.1) | | 12 (0.0) | |  |
| Border Terrier | 0.2 | [0.2, 0.2] | <0.001 | | 4978 (1.1) | | 121 (0.2) | |  |
| Soft Coated Wheaten Terrier | 0.2 | [0.1, 0.3] | <0.001 | | 742 (0.2) | | 18 (0.0) | |  |
| Dalmatian | 0.2 | [0.1, 0.2] | <0.001 | | 1625 (0.4) | | 37 (0.1) | |  |
| Irish Setter | 0.1 | [0.1, 0.2] | <0.001 | | 1430 (0.3) | | 27 (0.0) | |  |
| German Shepherd Dog | 0.1 | [0.1, 0.2] | <0.001 | | 12782 (2.9) | | 237 (0.3) | |  |
| Old English Sheepdog | 0.1 | [0.1, 0.2] | <0.001 | | 864 (0.2) | | 16 (0.0) | |  |
| Spaniel (Cocker) | 0.1 | [0.1, 0.1] | <0.001 | | 31119 (7.1) | | 573 (0.8) | |  |
| Kerry Blue Terrier | 0.1 | [0.1, 0.2] | <0.001 | | 632 (0.1) | | 11 (0.0) | |  |
| Akita | 0.1 | [0.0, 0.2] | <0.001 | | 886 (0.2) | | 10 (0.0) | |  |
| Airedale Terrier | 0.1 | [0.0, 0.1] | <0.001 | | 1104 (0.3) | | 9 (0.0) | |  |
| Cavalier King Charles Spaniel | 0 | [0.0, 0.1] | <0.001 | | 5502 (1.2) | | 30 (0.0) | |  |

## Supplementary table 7: Breed distribution according to Patnaik 3-tier scheme

| **Breed** | **Grade 1 (%)** | **Grade 2 (%)** | **Grade 3 (%)** | **Total** |
| --- | --- | --- | --- | --- |
| Gordon Setter | 0.0 | 57.1 | 42.9 | 14 |
| Leonberger | 0.0 | 70.6 | 29.4 | 17 |
| Shar Pei | 3.0 | 73.9 | 23.1 | 368 |
| Greyhound | 1.2 | 84.4 | 14.4 | 167 |
| Scottish Terrier | 8.6 | 77.1 | 14.3 | 35 |
| Dalmatian | 4.2 | 83.3 | 12.5 | 24 |
| Rottweiler | 3.3 | 85.1 | 11.6 | 276 |
| Italian Spinone | 3.8 | 84.6 | 11.5 | 26 |
| Italian Greyhound | 5.6 | 83.3 | 11.1 | 18 |
| Pomeranian | 0.0 | 88.9 | 11.1 | 18 |
| Whippet | 6.7 | 82.7 | 10.7 | 75 |
| Japanese Spitz | 0.0 | 90.0 | 10.0 | 10 |
| Briard | 0.0 | 90.9 | 9.1 | 11 |
| Yorkshire Terrier | 6.5 | 84.8 | 8.8 | 400 |
| Retriever (Flat Coated) | 9.8 | 81.7 | 8.5 | 82 |
| Mastiff | 4.5 | 87.0 | 8.4 | 154 |
| Dogue De Bordeaux | 5.6 | 86.1 | 8.3 | 72 |
| Newfoundland | 0.0 | 91.7 | 8.3 | 12 |
| Old English Sheepdog | 0.0 | 91.7 | 8.3 | 12 |
| Saluki | 0.0 | 91.7 | 8.3 | 12 |
| German Shorthaired Pointer | 1.4 | 90.4 | 8.2 | 73 |
| Basset Hound | 4.1 | 87.8 | 8.1 | 74 |
| West Highland White Terrier | 11.6 | 80.3 | 8.1 | 335 |
| Lhasa Apso | 11.9 | 80.1 | 8.0 | 176 |
| Border Collie | 3.0 | 89.0 | 7.9 | 365 |
| Lurcher | 4.4 | 87.7 | 7.9 | 203 |
| Dachshund (Miniature) | 2.6 | 89.7 | 7.7 | 39 |
| Unknown | 0.0 | 92.3 | 7.7 | 13 |
| Shih Tzu | 7.2 | 85.3 | 7.5 | 307 |
| Maltese | 0.0 | 92.7 | 7.3 | 55 |
| Belgian Shepherd Dog | 7.1 | 85.7 | 7.1 | 14 |
| Spaniel (Generic) | 3.1 | 89.9 | 7.0 | 129 |
| Dobermann | 6.9 | 86.1 | 6.9 | 101 |
| Manchester Terrier | 3.4 | 89.7 | 6.9 | 29 |
| Bulldog | 5.6 | 87.5 | 6.8 | 746 |
| Spaniel (Cocker) | 2.8 | 90.9 | 6.4 | 362 |
| Cavalier King Charles Spaniel | 6.2 | 87.5 | 6.2 | 16 |
| German Shepherd Dog | 3.1 | 90.7 | 6.2 | 162 |
| Retriever (Curly Coated) | 3.1 | 90.6 | 6.2 | 32 |
| Spaniel (Welsh Springer) | 0.0 | 93.8 | 6.2 | 16 |
| Cairn Terrier | 6.1 | 87.8 | 6.1 | 131 |
| Chihuahua | 9.5 | 84.4 | 6.1 | 147 |
| Dachshund (Standard) | 6.9 | 87.0 | 6.1 | 131 |
| Bichon Frise | 8.1 | 86.2 | 5.7 | 283 |
| Bullmastiff | 4.0 | 90.4 | 5.6 | 301 |
| Collie (Rough) | 4.9 | 89.5 | 5.6 | 143 |
| Jack Russell Terrier | 5.5 | 89.0 | 5.5 | 2,075 |
| Spaniel (Irish Water) | 8.1 | 86.5 | 5.4 | 37 |
| American Bulldog | 0.0 | 94.7 | 5.3 | 38 |
| Norfolk Terrier | 10.3 | 84.6 | 5.1 | 39 |
| Fox Terrier | 0.0 | 95.0 | 5.0 | 20 |
| Crossbreed | 5.5 | 89.5 | 4.9 | 7,992 |
| French Bulldog | 5.5 | 89.7 | 4.8 | 1,305 |
| Irish Terrier | 12.9 | 82.3 | 4.8 | 62 |
| Terrier (Generic) | 4.8 | 90.4 | 4.8 | 229 |
| Parson Russell Terrier | 14.0 | 81.4 | 4.7 | 43 |
| Siberian Husky | 3.9 | 91.3 | 4.7 | 127 |
| Bearded Collie | 0.0 | 95.5 | 4.5 | 22 |
| Schnauzer | 7.9 | 87.6 | 4.5 | 89 |
| Bernese Mountain Dog | 5.2 | 90.5 | 4.3 | 116 |
| Spaniel (English Springer) | 2.9 | 92.7 | 4.3 | 882 |
| Spaniel (Sussex) | 0.0 | 95.8 | 4.2 | 24 |
| Patterdale Terrier | 4.0 | 92.1 | 4.0 | 101 |
| Beagle | 3.2 | 93.0 | 3.8 | 688 |
| Retriever (Labrador) | 4.1 | 92.0 | 3.8 | 11,943 |
| Border Terrier | 2.5 | 93.8 | 3.7 | 81 |
| Tibetan Terrier | 14.3 | 82.1 | 3.6 | 56 |
| <na> | 4.0 | 92.6 | 3.3 | 10,818 |
| Miniature Schnauzer | 10.8 | 86.0 | 3.3 | 399 |
| Staffordshire Bull Terrier | 6.3 | 90.4 | 3.3 | 10,098 |
| Alaskan Malamute | 3.1 | 93.8 | 3.1 | 32 |
| Hungarian Vizsla | 7.3 | 89.7 | 3.0 | 301 |
| Labradoodle | 4.9 | 92.2 | 2.9 | 102 |
| Shetland Sheepdog | 12.5 | 84.7 | 2.8 | 72 |
| Retriever (Generic) | 1.7 | 95.8 | 2.5 | 530 |
| Poodle (Generic) | 4.5 | 93.2 | 2.3 | 44 |
| Weimaraner | 12.8 | 85.1 | 2.1 | 1,071 |
| Cockapoo | 4.1 | 93.9 | 2.0 | 49 |
| Boxer | 10.6 | 87.5 | 1.9 | 6,348 |
| English Pointer | 8.2 | 90.0 | 1.8 | 110 |
| Retriever (Golden) | 3.1 | 95.4 | 1.5 | 3,196 |
| Boston Terrier | 7.9 | 90.8 | 1.3 | 520 |
| Bull Terrier | 6.3 | 92.9 | 0.8 | 364 |
| Pug | 16.9 | 82.4 | 0.7 | 3,245 |
| Rhodesian Ridgeback | 3.8 | 95.7 | 0.5 | 209 |
| American Staffordshire Terrier | 9.6 | 90.4 | 0.0 | 52 |
| English Setter | 0.0 | 100.0 | 0.0 | 52 |
| Giant Schnauzer | 0.0 | 100.0 | 0.0 | 13 |
| Great Dane | 11.1 | 88.9 | 0.0 | 81 |
| Irish Setter | 0.0 | 100.0 | 0.0 | 20 |
| Papillon | 0.0 | 100.0 | 0.0 | 22 |
| Poodle (Standard) | 16.1 | 83.9 | 0.0 | 31 |
| Poodle (Toy) | 0.0 | 100.0 | 0.0 | 12 |
| Soft Coated Wheaten Terrier | 20.0 | 80.0 | 0.0 | 10 |
| Breeds with at least 10 cases of MCT diagnosed | | | | |

## Supplementary table 8: Breed distribution according to according to Kiupel 2-tier scheme

| Breed | Low-Grade(%) | High-Grade(%) | Total |
| --- | --- | --- | --- |
| Scottish Terrier | 41.2 | 58.8 | 17 |
| Gordon Setter | 41.7 | 58.3 | 12 |
| Newfoundland | 45.5 | 54.5 | 11 |
| Shar Pei | 45.9 | 54.1 | 268 |
| Belgian Shepherd Dog | 54.5 | 45.5 | 11 |
| Greyhound | 56.5 | 43.5 | 115 |
| Saluki | 60.0 | 40.0 | 10 |
| Whippet | 61.7 | 38.3 | 60 |
| West Highland White Terrier | 62.2 | 37.8 | 230 |
| Shih Tzu | 65.7 | 34.3 | 271 |
| Rottweiler | 68.2 | 31.8 | 151 |
| Cairn Terrier | 70.5 | 29.5 | 78 |
| Bearded Collie | 70.6 | 29.4 | 17 |
| Dogue De Bordeaux | 70.7 | 29.3 | 41 |
| Basset Hound | 71.2 | 28.8 | 59 |
| Yorkshire Terrier | 71.3 | 28.7 | 289 |
| Fox Terrier | 71.4 | 28.6 | 14 |
| Poodle (Standard) | 71.4 | 28.6 | 21 |
| Lhasa Apso | 72.0 | 28.0 | 125 |
| Spaniel (Generic) | 72.4 | 27.6 | 105 |
| Siberian Husky | 73.2 | 26.8 | 123 |
| Mastiff | 73.6 | 26.4 | 106 |
| Spaniel (Irish Water) | 74.2 | 25.8 | 31 |
| Bichon Frise | 74.4 | 25.6 | 242 |
| Pomeranian | 75.0 | 25.0 | 16 |
| Bullmastiff | 75.4 | 24.6 | 191 |
| Spaniel (Cocker) | 75.6 | 24.4 | 291 |
| Poodle (Generic) | 75.7 | 24.3 | 37 |
| Dobermann | 76.4 | 23.6 | 72 |
| German Shepherd Dog | 76.4 | 23.6 | 106 |
| Maltese | 76.6 | 23.4 | 47 |
| Jack Russell Terrier | 76.7 | 23.3 | 1,523 |
| Bulldog | 76.9 | 23.1 | 629 |
| Patterdale Terrier | 77.4 | 22.6 | 84 |
| Lurcher | 77.5 | 22.5 | 151 |
| Spaniel (English Springer) | 78.5 | 21.5 | 567 |
| Cavalier King Charles Spaniel | 78.6 | 21.4 | 14 |
| Schnauzer | 78.8 | 21.2 | 80 |
| Border Collie | 79.9 | 20.1 | 249 |
| Dalmatian | 80.0 | 20.0 | 15 |
| Norfolk Terrier | 80.0 | 20.0 | 20 |
| German Shorthaired Pointer | 80.6 | 19.4 | 67 |
| American Staffordshire Terrier | 80.8 | 19.2 | 52 |
| Parson Russell Terrier | 81.1 | 18.9 | 37 |
| Border Terrier | 81.2 | 18.8 | 64 |
| Chihuahua | 81.7 | 18.3 | 142 |
| French Bulldog | 81.9 | 18.1 | 1,307 |
| Terrier (Generic) | 82.2 | 17.8 | 180 |
| English Pointer | 83.0 | 17.0 | 88 |
| Bernese Mountain Dog | 83.3 | 16.7 | 78 |
| <na> | 83.5 | 16.5 | 10,229 |
| Retriever (Labrador) | 83.6 | 16.4 | 8,204 |
| Hungarian Vizsla | 83.7 | 16.3 | 252 |
| Crossbreed | 83.8 | 16.2 | 6,159 |
| Beagle | 84.3 | 15.7 | 586 |
| Retriever (Flat Coated) | 85.2 | 14.8 | 54 |
| Miniature Schnauzer | 85.4 | 14.6 | 316 |
| Papillon | 85.7 | 14.3 | 14 |
| Tibetan Terrier | 85.7 | 14.3 | 42 |
| Labradoodle | 86.2 | 13.8 | 109 |
| Collie (Rough) | 86.4 | 13.6 | 81 |
| Italian Spinone | 86.7 | 13.3 | 15 |
| Staffordshire Bull Terrier | 87.1 | 12.9 | 7,581 |
| Retriever (Generic) | 87.5 | 12.5 | 272 |
| Shetland Sheepdog | 87.5 | 12.5 | 56 |
| Cockapoo | 88.7 | 11.3 | 53 |
| Retriever (Curly Coated) | 88.9 | 11.1 | 18 |
| Rhodesian Ridgeback | 89.2 | 10.8 | 148 |
| Dachshund (Standard) | 89.4 | 10.6 | 104 |
| Boxer | 89.6 | 10.4 | 3,690 |
| American Bulldog | 89.7 | 10.3 | 39 |
| Alaskan Malamute | 90.3 | 9.7 | 31 |
| Dachshund (Miniature) | 90.3 | 9.7 | 31 |
| Manchester Terrier | 90.5 | 9.5 | 21 |
| Soft Coated Wheaten Terrier | 90.9 | 9.1 | 11 |
| Spaniel (Sussex) | 90.9 | 9.1 | 11 |
| Great Dane | 91.1 | 8.9 | 56 |
| Weimaraner | 91.1 | 8.9 | 637 |
| Irish Setter | 91.7 | 8.3 | 12 |
| Retriever (Golden) | 91.9 | 8.1 | 2,111 |
| Unknown | 92.9 | 7.1 | 14 |
| Bull Terrier | 93.7 | 6.3 | 252 |
| Italian Greyhound | 93.8 | 6.2 | 16 |
| Boston Terrier | 94.0 | 6.0 | 501 |
| Irish Terrier | 94.6 | 5.4 | 56 |
| Pug | 95.1 | 4.9 | 2,988 |
| English Setter | 97.9 | 2.1 | 47 |
| Breeds with at least 10 cases of MCT diagnosed | | | |

## Supplementary table 9: emmeans for pairwise comparison between sex and neuter status for mast cell tumours.

| **Comparison** | **OR** | **SE** | **Reference value** | **Z ratio** | **95% CI** | **p-value** |
| --- | --- | --- | --- | --- | --- | --- |
| FN - FE within [3,6) | 1.8 | 0,059 | 1 | 10,086 | [1.6, 2.0] | <0.001 |
| FN - MN within [3,6) | 1.1 | 0,043 | 1 | 1,602 | [1.0, 1.2] | 0.375 |
| FN - ME within [3,6) | 1.5 | 0,055 | 1 | 7,145 | [1.3, 1.6] | <0.001 |
| FE - MN within [3,6) | 0.6 | 0,06 | 1 | -8,769 | [0.5, 0.7] | <0.001 |
| FE - ME within [3,6) | 0.8 | 0,069 | 1 | -2,929 | [0.7, 0.9] | 0.018 |
| MN - ME within [3,6) | 1.4 | 0,056 | 1 | 5,787 | [1.2, 1.5] | <0.001 |
| FN - FE within [6,9) | 1.7 | 0,045 | 1 | 12,273 | [1.6, 1.9] | <0.001 |
| FN - MN within [6,9) | 1.2 | 0,032 | 1 | 4,888 | [1.1, 1.2] | <0.001 |
| FN - ME within [6,9) | 1.7 | 0,043 | 1 | 13 | [1.6, 1.9] | <0.001 |
| FE - MN within [6,9) | 0.7 | 0,046 | 1 | -8,606 | [0.6, 0.7] | <0.001 |
| FE - ME within [6,9) | 1 | 0,054 | 1 | 0,124 | [0.9, 1.1] | 0.999 |
| MN - ME within [6,9) | 1.5 | 0,044 | 1 | 9,149 | [1.4, 1.6] | <0.001 |
| FN - FE within [9,12) | 1.7 | 0,047 | 1 | 11,16 | [1.5, 1.9] | <0.001 |
| FN - MN within [9,12) | 1.3 | 0,035 | 1 | 7,255 | [1.2, 1.4] | <0.001 |
| FN - ME within [9,12) | 1.9 | 0,045 | 1 | 14,73 | [1.8, 2.1] | <0.001 |
| FE - MN within [9,12) | 0.8 | 0,049 | 1 | -5,523 | [0.7, 0.8] | <0.001 |
| FE - ME within [9,12) | 1.1 | 0,056 | 1 | 2,469 | [1.0, 1.3] | 0.067 |
| MN - ME within [9,12) | 1.5 | 0,047 | 1 | 8,7 | [1.4, 1.7] | <0.001 |
| FN - FE within [12,15) | 1.7 | 0,081 | 1 | 6,793 | [1.5, 2.0] | <0.001 |
| FN - MN within [12,15) | 1.2 | 0,059 | 1 | 2,963 | [1.1, 1.3] | 0.015 |
| FN - ME within [12,15) | 2 | 0,079 | 1 | 8,656 | [1.7, 2.3] | <0.001 |
| FE - MN within [12,15) | 0.7 | 0,085 | 1 | -4,416 | [0.6, 0.8] | <0.001 |
| FE - ME within [12,15) | 1.1 | 0,1 | 1 | 1,336 | [0.9, 1.4] | 0.541 |
| MN - ME within [12,15) | 1.7 | 0,083 | 1 | 6,132 | [1.4, 2.0] | <0.001 |
| FN - FE within [15,18] | 2.3 | 0,304 | 1 | 2,732 | [1.3, 4.2] | 0.032 |
| FN - MN within [15,18] | 1.3 | 0,213 | 1 | 1,063 | [0.8, 1.9] | 0.712 |
| FN - ME within [15,18] | 1.3 | 0,246 | 1 | 1,179 | [0.8, 2.2] | 0.639 |
| FE - MN within [15,18] | 0.5 | 0,323 | 1 | -1,87 | [0.3, 1.0] | 0.241 |
| FE - ME within [15,18] | 0.6 | 0,345 | 1 | -1,567 | [0.3, 1.1] | 0.398 |
| MN - ME within [15,18] | 1.1 | 0,269 | 1 | 0,236 | [0.6, 1.8] | 0.995 |

## Supplementary table 10: Full data table from the multivariable logistic regression analysis for melanomas.

### Table 10.a: Oral melanomas

| **Effect** | **Value** | **OR** | **95% CI** | **p-value** | **n (%) (Controls)** | **n (%) (Cases)** |
| --- | --- | --- | --- | --- | --- | --- |
| Breed | Rottweiler | 12.2 | [9.5, 15.6] | <0.001 | 580 (0.7) | 118 (3.0) |
| Breed | Shar Pei | 10.3 | [6.1, 17.4] | <0.001 | 174 (0.2) | 24 (0.6) |
| Breed | Chow Chow | 8.7 | [3.6, 21.1] | <0.001 | 52 (0.1) | 11 (0.3) |
| Breed | Mastiff | 8.7 | [2.9, 25.4] | <0.001 | 53 (0.1) | 5 (0.1) |
| Breed | Bernese Mountain Dog | 8.5 | [3.9, 18.6] | <0.001 | 116 (0.1) | 8 (0.2) |
| Breed | Dogue De Bordeaux | 7 | [2.7, 18.0] | <0.001 | 128 (0.1) | 5 (0.1) |
| Breed | Newfoundland | 6.9 | [3.9, 12.2] | <0.001 | 143 (0.2) | 16 (0.4) |
| Breed | Bullmastiff | 6.1 | [2.7, 13.8] | <0.001 | 112 (0.1) | 9 (0.2) |
| Breed | Hungarian Vizsla | 5.4 | [3.5, 8.4] | <0.001 | 358 (0.4) | 29 (0.7) |
| Breed | Irish Setter | 5.4 | [3.5, 8.4] | <0.001 | 223 (0.3) | 29 (0.7) |
| Breed | Spaniel (Generic) | 5.2 | [3.2, 8.6] | <0.001 | 110 (0.1) | 27 (0.7) |
| Breed | Kerry Blue Terrier | 5.1 | [1.7, 15.4] | 0.00 | 33 (0.0) | 7 (0.2) |
| Breed | Pug | 5.1 | [3.6, 7.0] | <0.001 | 795 (0.9) | 53 (1.4) |
| Breed | Scottish Terrier | 5 | [3.4, 7.6] | <0.001 | 221 (0.3) | 34 (0.9) |
| Breed | Retriever (Golden) | 4.6 | [3.9, 5.5] | <0.001 | 1912 (2.2) | 286 (7.4) |
| Breed | Great Dane | 4.5 | [2.0, 9.9] | <0.001 | 154 (0.2) | 8 (0.2) |
| Breed | Retriever (Generic) | 4.4 | [3.0, 6.6] | <0.001 | 208 (0.2) | 36 (0.9) |
| Breed | Alaskan Malamute | 4.3 | [2.2, 8.7] | <0.001 | 144 (0.2) | 10 (0.3) |
| Breed | Pekingese | 4.1 | [1.8, 9.4] | <0.001 | 58 (0.1) | 7 (0.2) |
| Breed | Retriever (Flat Coated) | 3.9 | [2.4, 6.6] | <0.001 | 301 (0.3) | 21 (0.5) |
| Breed | Spaniel (Cocker) | 3.6 | [3.2, 4.2] | <0.001 | 5382 (6.1) | 456 (11.7) |
| Breed | French Bulldog | 3.5 | [1.7, 7.0] | <0.001 | 577 (0.7) | 9 (0.2) |
| Breed | Bulldog | 3.4 | [1.9, 6.1] | <0.001 | 493 (0.6) | 14 (0.4) |
| Breed | Tibetan Spaniel | 3.2 | [1.2, 8.7] | 0.02 | 34 (0.0) | 5 (0.1) |
| Breed | Welsh Terrier | 3.2 | [1.5, 6.7] | 0.00 | 90 (0.1) | 10 (0.3) |
| Breed | Cockapoo | 3.1 | [1.9, 5.2] | <0.001 | 881 (1.0) | 19 (0.5) |
| Breed | Dachshund (Standard) | 3 | [2.2, 4.2] | <0.001 | 727 (0.8) | 53 (1.4) |
| Breed | Poodle (Generic) | 3 | [1.8, 5.0] | <0.001 | 264 (0.3) | 23 (0.6) |
| Breed | Tibetan Terrier | 3 | [1.9, 4.7] | <0.001 | 255 (0.3) | 25 (0.6) |
| Breed | Collie (Rough) | 2.9 | [1.8, 4.6] | <0.001 | 274 (0.3) | 23 (0.6) |
| Breed | English Pointer | 2.8 | [1.6, 4.9] | <0.001 | 237 (0.3) | 16 (0.4) |
| Breed | Norfolk Terrier | 2.6 | [1.4, 5.0] | 0.00 | 185 (0.2) | 12 (0.3) |
| Breed | Airedale Terrier | 2.5 | [1.3, 4.9] | 0.01 | 146 (0.2) | 11 (0.3) |
| Breed | Beagle | 2.4 | [1.6, 3.6] | <0.001 | 658 (0.7) | 31 (0.8) |
| Breed | Staffordshire Bull Terrier | 2.4 | [2.0, 2.8] | <0.001 | 3081 (3.5) | 230 (5.9) |
| Breed | Labradoodle | 2.2 | [1.3, 3.6] | 0.00 | 686 (0.8) | 19 (0.5) |
| Breed | Retriever (Labrador) | 2.1 | [1.8, 2.4] | <0.001 | 10863 (12.3) | 676 (17.4) |
| Breed | Schnauzer | 2 | [1.0, 3.9] | 0.06 | 198 (0.2) | 9 (0.2) |
| Breed | German Shorthaired Pointer | 1.8 | [1.0, 3.1] | 0.05 | 304 (0.3) | 15 (0.4) |
| Breed | Rhodesian Ridgeback | 1.8 | [0.8, 3.9] | 0.14 | 218 (0.2) | 8 (0.2) |
| Breed | Shetland Sheepdog | 1.8 | [1.0, 3.3] | 0.06 | 237 (0.3) | 13 (0.3) |
| Breed | Miniature Schnauzer | 1.7 | [1.2, 2.5] | 0.00 | 867 (1.0) | 37 (1.0) |
| Breed | Siberian Husky | 1.7 | [1.0, 3.0] | 0.05 | 441 (0.5) | 17 (0.4) |
| Breed | Dachshund (Miniature) | 1.6 | [1.0, 2.6] | 0.05 | 486 (0.5) | 21 (0.5) |
| Breed | Basset Hound | 1.5 | [0.7, 3.1] | 0.27 | 241 (0.3) | 8 (0.2) |
| Breed | Cavalier King Charles Spaniel | 1.4 | [1.0, 1.9] | 0.05 | 1766 (2.0) | 57 (1.5) |
| Breed | Dobermann | 1.4 | [0.6, 3.2] | 0.44 | 283 (0.3) | 7 (0.2) |
| Breed | Poodle (Standard) | 1.4 | [0.9, 2.3] | 0.17 | 429 (0.5) | 23 (0.6) |
| Breed | Border Terrier | 1.1 | [0.9, 1.4] | 0.45 | 2030 (2.3) | 76 (2.0) |
| Breed | German Shepherd Dog | 1.1 | [0.8, 1.5] | 0.63 | 2154 (2.4) | 56 (1.4) |
| Breed | Crossbreed | 1 |  |  | 17598 (19.9) | 590 (15.2) |
| Breed | Pomeranian | 1 | [0.4, 2.6] | 0.93 | 267 (0.3) | 6 (0.2) |
| Breed | Spaniel (Welsh Springer) | 1 | [0.4, 2.8] | 0.99 | 136 (0.2) | 6 (0.2) |
| Breed | Lakeland Terrier | 0.8 | [0.3, 1.7] | 0.50 | 223 (0.3) | 7 (0.2) |
| Breed | Patterdale Terrier | 0.8 | [0.4, 1.5] | 0.42 | 583 (0.7) | 10 (0.3) |
| Breed | Weimaraner | 0.8 | [0.4, 1.5] | 0.48 | 396 (0.4) | 10 (0.3) |
| Breed | Yorkshire Terrier | 0.8 | [0.6, 1.1] | 0.21 | 2167 (2.5) | 64 (1.6) |
| Breed | Jack Russell Terrier | 0.7 | [0.6, 0.8] | <0.001 | 6181 (7.0) | 145 (3.7) |
| Breed | Shih Tzu | 0.7 | [0.5, 1.1] | 0.14 | 1663 (1.9) | 32 (0.8) |
| Breed | Spaniel (English Springer) | 0.7 | [0.6, 0.9] | 0.01 | 3896 (4.4) | 89 (2.3) |
| Breed | Border Collie | 0.6 | [0.5, 0.7] | <0.001 | 4543 (5.1) | 88 (2.3) |
| Breed | King Charles Spaniel | 0.6 | [0.2, 1.5] | 0.26 | 382 (0.4) | 6 (0.2) |
| Breed | Poodle (Toy) | 0.6 | [0.3, 1.4] | 0.22 | 291 (0.3) | 7 (0.2) |
| Breed | Terrier (Generic) | 0.6 | [0.3, 0.9] | 0.03 | 808 (0.9) | 18 (0.5) |
| Breed | Boxer | 0.5 | [0.2, 0.9] | 0.02 | 1079 (1.2) | 10 (0.3) |
| Breed | Chihuahua | 0.5 | [0.2, 1.0] | 0.04 | 1085 (1.2) | 10 (0.3) |
| Breed | Lhasa Apso | 0.5 | [0.3, 0.8] | 0.01 | 1102 (1.2) | 17 (0.4) |
| Breed | Lurcher | 0.5 | [0.3, 0.8] | 0.001 | 1205 (1.4) | 16 (0.4) |
| Breed | Cairn Terrier | 0.4 | [0.2, 0.9] | 0.02 | 516 (0.6) | 7 (0.2) |
| Breed | Greyhound | 0.2 | [0.1, 0.5] | <0.001 | 1253 (1.4) | 6 (0.2) |
| Breed | West Highland White Terrier | 0.2 | [0.1, 0.3] | <0.001 | 2646 (3.0) | 23 (0.6) |
| Sex | Female neutered | 1 |  |  | 30083 (34.0) | 1106 (28.4) |
| Sex | Female entire | 0.2 | [0.1, 0.8] | 0.02 | 15202 (17.2) | 278 (7.1) |
| Sex | Male neutered | 1.2 | [0.7, 2.3] | 0.47 | 26466 (29.9) | 1342 (34.5) |
| Sex | Male entire | 0.8 | [0.4, 1.6] | 0.50 | 16631 (18.8) | 746 (19.2) |
| Age | [3,6) | 1 |  |  | 25577 (28.9) | 64 (1.6) |
| Age | [6,9) | 6.6 | [4.1, 10.5] | <0.001 | 23250 (26.3) | 470 (12.1) |
| Age | [9,12) | 28.6 | [18.2, 45.0] | <0.001 | 18423 (20.8) | 1609 (41.4) |
| Age | [12,15) | 59.3 | [37.7, 93.1] | <0.001 | 9970 (11.3) | 1533 (39.4) |
| Age | [15,18] | 62.8 | [37.9, 104.2] | <0.001 | 2001 (2.3) | 210 (5.4) |

### Table 10.b: Cutaneous melanomas

| **Effect** | **Value** | **OR** | **95% CI** | **p-value** | **n (%) (Controls)** | **n (%) (Cases)** | |
| --- | --- | --- | --- | --- | --- | --- | --- |
| Breed | Shar Pei | 16.2 | [10.6, 24.6] | <0.001 | 174 (0.2) | 34 (1.7) |  |
| Breed | Hungarian Vizsla | 15.5 | [11.6, 20.8] | <0.001 | 358 (0.4) | 77 (3.7) |  |
| Breed | Chow Chow | 12.4 | [5.8, 26.3] | <0.001 | 52 (0.1) | 9 (0.4) |  |
| Breed | Rottweiler | 10.6 | [8.1, 13.7] | <0.001 | 580 (0.7) | 102 (5.0) |  |
| Breed | Giant Schnauzer | 9.5 | [4.3, 20.9] | <0.001 | 42 (0.0) | 9 (0.4) |  |
| Breed | Mastiff | 9.5 | [3.9, 22.9] | <0.001 | 53 (0.1) | 6 (0.3) |  |
| Breed | Dogue De Bordeaux | 8.2 | [4.1, 16.7] | <0.001 | 128 (0.1) | 9 (0.4) |  |
| Breed | Irish Terrier | 8 | [4.1, 15.6] | <0.001 | 72 (0.1) | 11 (0.5) |  |
| Breed | Bernese Mountain Dog | 6.1 | [2.9, 12.8] | <0.001 | 116 (0.1) | 8 (0.4) |  |
| Breed | Rhodesian Ridgeback | 5.6 | [3.4, 9.2] | <0.001 | 218 (0.2) | 21 (1.0) |  |
| Breed | Dobermann | 5.5 | [3.5, 8.7] | <0.001 | 283 (0.3) | 24 (1.2) |  |
| Breed | Scottish Terrier | 4.8 | [3.0, 7.5] | <0.001 | 221 (0.3) | 27 (1.3) |  |
| Breed | Irish Setter | 4.6 | [2.8, 7.7] | <0.001 | 223 (0.3) | 21 (1.0) |  |
| Breed | Bullmastiff | 4.2 | [1.8, 9.8] | <0.001 | 112 (0.1) | 7 (0.3) |  |
| Breed | Chinese Crested | 4.2 | [1.8, 9.9] | <0.001 | 88 (0.1) | 6 (0.3) |  |
| Breed | Miniature Schnauzer | 4.2 | [3.1, 5.6] | <0.001 | 867 (1.0) | 67 (3.3) |  |
| Breed | Poodle (Generic) | 3.9 | [2.3, 6.5] | <0.001 | 264 (0.3) | 19 (0.9) |  |
| Breed | French Bulldog | 3.5 | [1.9, 6.1] | <0.001 | 577 (0.7) | 15 (0.7) |  |
| Breed | Schnauzer | 3.5 | [1.9, 6.4] | <0.001 | 198 (0.2) | 12 (0.6) |  |
| Breed | Airedale Terrier | 3.4 | [1.7, 6.5] | <0.001 | 146 (0.2) | 10 (0.5) |  |
| Breed | Retriever (Flat Coated) | 3.2 | [1.8, 5.6] | <0.001 | 301 (0.3) | 18 (0.9) |  |
| Breed | Spaniel (Generic) | 3.2 | [1.6, 6.8] | 0.00 | 110 (0.1) | 8 (0.4) |  |
| Breed | Welsh Terrier | 3 | [1.2, 7.5] | 0.02 | 90 (0.1) | 5 (0.2) |  |
| Breed | Newfoundland | 2.5 | [1.0, 6.3] | 0.05 | 143 (0.2) | 5 (0.2) |  |
| Breed | Retriever (Generic) | 2.4 | [1.3, 4.4] | 0.01 | 208 (0.2) | 13 (0.6) |  |
| Breed | Bulldog | 2.2 | [1.2, 4.3] | 0.01 | 493 (0.6) | 11 (0.5) |  |
| Breed | Retriever (Golden) | 2.2 | [1.7, 2.8] | <0.001 | 1912 (2.2) | 85 (4.1) |  |
| Breed | Staffordshire Bull Terrier | 2.2 | [1.8, 2.7] | <0.001 | 3081 (3.5) | 145 (7.0) |  |
| Breed | Pug | 2.1 | [1.3, 3.4] | 0.00 | 795 (0.9) | 22 (1.1) |  |
| Breed | Boxer | 1.9 | [1.3, 2.7] | <0.001 | 1079 (1.2) | 36 (1.7) |  |
| Breed | Dachshund (Standard) | 1.7 | [1.0, 2.8] | 0.03 | 727 (0.8) | 20 (1.0) |  |
| Breed | Lakeland Terrier | 1.5 | [0.7, 3.3] | 0.28 | 223 (0.3) | 7 (0.3) |  |
| Breed | Patterdale Terrier | 1.5 | [0.9, 2.5] | 0.17 | 583 (0.7) | 16 (0.8) |  |
| Breed | Spaniel (Cocker) | 1.4 | [1.2, 1.8] | <0.001 | 5384 (6.1) | 136 (6.6) |  |
| Breed | German Shepherd Dog | 1.3 | [0.9, 1.8] | 0.11 | 2154 (2.4) | 49 (2.4) |  |
| Breed | Norfolk Terrier | 1.3 | [0.5, 3.6] | 0.59 | 185 (0.2) | 6 (0.3) |  |
| Breed | Labradoodle | 1.2 | [0.7, 2.3] | 0.52 | 686 (0.8) | 11 (0.5) |  |
| Breed | Retriever (Labrador) | 1.2 | [1.0, 1.4] | 0.02 | 10863 (12.3) | 263 (12.8) |  |
| Breed | Tibetan Terrier | 1.2 | [0.5, 2.7] | 0.71 | 255 (0.3) | 6 (0.3) |  |
| Breed | Yorkshire Terrier | 1.2 | [0.9, 1.7] | 0.16 | 2167 (2.5) | 55 (2.7) |  |
| Breed | Weimaraner | 1.1 | [0.6, 2.2] | 0.79 | 396 (0.5) | 10 (0.5) |  |
| Breed | Crossbreed | 1 |  |  | 17602 (20.0) | 345 (16.8) |  |
| Breed | Bull Terrier | 1 | [0.4, 2.8] | 0.95 | 231 (0.3) | 5 (0.2) |  |
| Breed | Cairn Terrier | 1 | [0.6, 1.9] | 0.92 | 516 (0.6) | 12 (0.6) |  |
| Breed | Cockapoo | 1 | [0.5, 2.2] | 0.91 | 881 (1.0) | 10 (0.5) |  |
| Breed | Collie (Rough) | 1 | [0.4, 2.5] | 0.98 | 274 (0.3) | 6 (0.3) |  |
| Breed | Dachshund (Miniature) | 1 | [0.5, 2.0] | 0.93 | 486 (0.6) | 9 (0.4) |  |
| Breed | Terrier (Generic) | 1 | [0.6, 1.7] | 0.89 | 809 (0.9) | 21 (1.0) |  |
| Breed | German Shorthaired Pointer | 0.9 | [0.4, 2.3] | 0.88 | 304 (0.3) | 5 (0.2) |  |
| Breed | Beagle | 0.8 | [0.4, 1.7] | 0.60 | 658 (0.7) | 9 (0.4) |  |
| Breed | Border Terrier | 0.7 | [0.5, 1.1] | 0.14 | 2030 (2.3) | 29 (1.4) |  |
| Breed | Shih Tzu | 0.7 | [0.5, 1.2] | 0.20 | 1663 (1.9) | 20 (1.0) |  |
| Breed | Chihuahua | 0.6 | [0.3, 1.2] | 0.13 | 1085 (1.2) | 8 (0.4) |  |
| Breed | Poodle (Standard) | 0.6 | [0.3, 1.5] | 0.28 | 429 (0.5) | 6 (0.3) |  |
| Breed | Cavalier King Charles Spaniel | 0.4 | [0.2, 0.7] | 0.00 | 1766 (2.0) | 15 (0.7) |  |
| Breed | Lurcher | 0.4 | [0.2, 0.8] | 0.01 | 1205 (1.4) | 9 (0.4) |  |
| Breed | Whippet | 0.4 | [0.2, 1.0] | 0.06 | 770 (0.9) | 6 (0.3) |  |
| Breed | Bichon Frise | 0.3 | [0.1, 0.7] | 0.001 | 1029 (1.2) | 5 (0.2) |  |
| Breed | Greyhound | 0.3 | [0.1, 0.7] | 0.001 | 1253 (1.4) | 7 (0.3) |  |
| Breed | Jack Russell Terrier | 0.3 | [0.2, 0.5] | <0.001 | 6181 (7.0) | 43 (2.1) |  |
| Breed | Lhasa Apso | 0.3 | [0.2, 0.7] | 0.01 | 1102 (1.3) | 7 (0.3) |  |
| Breed | Spaniel (English Springer) | 0.3 | [0.2, 0.5] | <0.001 | 3896 (4.4) | 25 (1.2) |  |
| Breed | Border Collie | 0.2 | [0.2, 0.4] | <0.001 | 4543 (5.2) | 24 (1.2) |  |
| Breed | West Highland White Terrier | 0.2 | [0.1, 0.4] | <0.001 | 2646 (3.0) | 12 (0.6) |  |
| Sex | Female neutered | 1 |  |  | 29926 (34.0) | 627 (30.5) |  |
| Sex | Female entire | 0.6 | [0.4, 1.0] | 0.07 | 15088 (17.2) | 171 (8.3) |  |
| Sex | Male neutered | 1.4 | [1.0, 2.1] | 0.07 | 26462 (30.1) | 714 (34.7) |  |
| Sex | Male entire | 1.7 | [1.1, 2.5] | 0.01 | 16490 (18.7) | 378 (18.4) |  |
| Age | [3,6) | 1 |  |  | 25490 (29.0) | 186 (9.0) |  |
| Age | [6,9) | 3.8 | [2.7, 5.2] | <0.001 | 23083 (26.2) | 593 (28.8) |  |
| Age | [9,12) | 7.1 | [5.1, 9.7] | <0.001 | 18380 (20.9) | 811 (39.4) |  |
| Age | [12,15) | 8.6 | [6.2, 12.1] | <0.001 | 9916 (11.3) | 416 (20.2) |  |
| Age | [15,18] | 6.1 | [3.5, 10.8] | <0.001 | 1994 (2.3) | 44 (2.1) |  |

### Table 10.c: Cutaneous digital melanomas

| **Effect** | **Value** | **OR** | **95% CI** | **p-value** | **n (%) (Controls)** | **n (%) (Cases)** |
| --- | --- | --- | --- | --- | --- | --- |
| Breed | Giant Schnauzer | 50.3 | [24.7, 102.5] | <0.001 | 42 (0.1) | 13 (1.6) |
| Breed | Rottweiler | 37.5 | [27.5, 51.2] | <0.001 | 580 (0.9) | 98 (11.7) |
| Breed | Scottish Terrier | 19.6 | [12.5, 30.9] | <0.001 | 221 (0.4) | 30 (3.6) |
| Breed | Bullmastiff | 14.8 | [6.2, 35.3] | <0.001 | 112 (0.2) | 7 (0.8) |
| Breed | Schnauzer | 12.2 | [6.5, 23.0] | <0.001 | 198 (0.3) | 12 (1.4) |
| Breed | Miniature Schnauzer | 9.9 | [6.8, 14.5] | <0.001 | 867 (1.4) | 49 (5.9) |
| Breed | Retriever (Flat Coated) | 9.5 | [5.1, 17.8] | <0.001 | 301 (0.5) | 14 (1.7) |
| Breed | Dobermann | 6.6 | [3.2, 14.0] | <0.001 | 283 (0.5) | 8 (1.0) |
| Breed | Hungarian Vizsla | 4.4 | [1.9, 10.2] | <0.001 | 358 (0.6) | 7 (0.8) |
| Breed | English Pointer | 4.3 | [1.7, 10.8] | 0.00 | 237 (0.4) | 5 (0.6) |
| Breed | Retriever (Labrador) | 3.9 | [3.1, 5.0] | <0.001 | 10863 (17.3) | 259 (31.0) |
| Breed | Poodle (Generic) | 3.8 | [1.5, 9.6] | 0.00 | 264 (0.4) | 5 (0.6) |
| Breed | Irish Setter | 3.4 | [1.2, 9.4] | 0.02 | 223 (0.4) | 6 (0.7) |
| Breed | Retriever (Generic) | 3.4 | [1.4, 8.5] | 0.01 | 208 (0.3) | 6 (0.7) |
| Breed | Pug | 2.7 | [1.3, 5.9] | 0.01 | 795 (1.3) | 7 (0.8) |
| Breed | Retriever (Golden) | 2.7 | [1.8, 4.1] | <0.001 | 1912 (3.0) | 35 (4.2) |
| Breed | Labradoodle | 2.2 | [0.9, 5.5] | 0.09 | 686 (1.1) | 7 (0.8) |
| Breed | Spaniel (Cocker) | 2.2 | [1.6, 3.1] | <0.001 | 5383 (8.6) | 63 (7.5) |
| Breed | German Shepherd Dog | 1.9 | [1.2, 3.1] | 0.01 | 2154 (3.4) | 24 (2.9) |
| Breed | Boxer | 1.3 | [0.6, 2.8] | 0.54 | 1079 (1.7) | 7 (0.8) |
| Breed | Crossbreed | 1 |  |  | 17602 (28.1) | 106 (12.7) |
| Breed | West Highland White Terrier | 1 | [0.6, 1.7] | 0.96 | 2646 (4.2) | 19 (2.3) |
| Breed | Border Terrier | 0.9 | [0.4, 1.7] | 0.66 | 2030 (3.2) | 10 (1.2) |
| Breed | Spaniel (English Springer) | 0.6 | [0.4, 1.1] | 0.12 | 3896 (6.2) | 14 (1.7) |
| Breed | Staffordshire Bull Terrier | 0.5 | [0.3, 1.0] | 0.07 | 3081 (4.9) | 11 (1.3) |
| Breed | Yorkshire Terrier | 0.5 | [0.2, 1.1] | 0.08 | 2167 (3.5) | 6 (0.7) |
| Breed | Border Collie | 0.2 | [0.1, 0.5] | <0.001 | 4543 (7.2) | 7 (0.8) |
| Sex | Female neutered | 1 |  |  | 21504 (34.3) | 263 (31.5) |
| Sex | Female entire | 0.5 | [0.2, 1.5] | 0.20 | 10529 (16.8) | 59 (7.1) |
| Sex | Male neutered | 1.3 | [0.6, 2.6] | 0.52 | 18718 (29.8) | 289 (34.6) |
| Age | [3,6) | 1 |  |  | 17717 (28.2) | 52 (6.2) |
| Age | [6,9) | 4 | [2.3, 7.2] | <0.001 | 16387 (26.1) | 224 (26.8) |
| Age | [9,12) | 10.3 | [5.9, 17.9] | <0.001 | 13513 (21.5) | 375 (44.9) |
| Age | [12,15) | 11.4 | [6.3, 20.5] | <0.001 | 7344 (11.7) | 174 (20.8) |
| Age | [15,18] | 4.4 | [1.2, 15.4] | 0.02 | 1365 (2.2) | 9 (1.1) |

### Table 10.d: Uveal melanomas

| **Effect** | **Value** | | | **OR** | **95% CI** | | **p-value** | **n (%) (Controls)** | | **n (%) (Cases)** | |
| --- | --- | --- | --- | --- | --- | --- | --- | --- | --- | --- | --- |
| Breed | Dogue De Bordeaux | 30.1 | [14.9, 60.7] | | | <0.001 | 128 (0.2) | | 14 (2.2) | |  |
| Breed | Hungarian Vizsla | 18.1 | [11.5, 28.7] | | | <0.001 | 358 (0.5) | | 28 (4.3) | |  |
| Breed | Irish Terrier | 17.8 | [7.8, 40.3] | | | <0.001 | 72 (0.1) | | 7 (1.1) | |  |
| Breed | Shar Pei | 17.5 | [9.1, 34.0] | | | <0.001 | 174 (0.3) | | 11 (1.7) | |  |
| Breed | Spaniel (Generic) | 12.8 | [6.2, 26.4] | | | <0.001 | 110 (0.2) | | 10 (1.6) | |  |
| Breed | Bernese Mountain Dog | 11.9 | [4.7, 30.2] | | | <0.001 | 116 (0.2) | | 5 (0.8) | |  |
| Breed | Rottweiler | 7.3 | [4.4, 12.0] | | | <0.001 | 580 (0.8) | | 23 (3.6) | |  |
| Breed | Norfolk Terrier | 6.6 | [2.8, 15.5] | | | <0.001 | 185 (0.3) | | 6 (0.9) | |  |
| Breed | Rhodesian Ridgeback | 4.8 | [1.9, 12.0] | | | <0.001 | 218 (0.3) | | 5 (0.8) | |  |
| Breed | Bull Terrier | 4.3 | [1.7, 10.8] | | | 0.001 | 231 (0.3) | | 5 (0.8) | |  |
| Breed | Dobermann | 4 | [1.6, 10.0] | | | 0.001 | 283 (0.4) | | 6 (0.9) | |  |
| Breed | Beagle | 3.7 | [1.9, 6.9] | | | <0.001 | 658 (0.9) | | 11 (1.7) | |  |
| Breed | Shetland Sheepdog | 3 | [1.1, 8.2] | | | 0.03 | 237 (0.3) | | 5 (0.8) | |  |
| Breed | Miniature Schnauzer | 2.5 | [1.3, 4.7] | | | 0.001 | 867 (1.2) | | 12 (1.9) | |  |
| Breed | Staffordshire Bull Terrier | 2.5 | [1.7, 3.6] | | | <0.001 | 3081 (4.4) | | 50 (7.8) | |  |
| Breed | Spaniel (Cocker) | 2.3 | [1.6, 3.2] | | | <0.001 | 5384 (7.8) | | 61 (9.5) | |  |
| Breed | Dachshund (Standard) | 2.2 | [1.0, 4.9] | | | 0.04 | 727 (1.0) | | 8 (1.2) | |  |
| Breed | Retriever (Golden) | 2.1 | [1.3, 3.3] | | | 0.001 | 1912 (2.8) | | 26 (4.0) | |  |
| Breed | Boxer | 1.8 | [0.9, 3.4] | | | 0.09 | 1079 (1.6) | | 10 (1.6) | |  |
| Breed | German Shepherd Dog | 1.6 | [0.9, 2.7] | | | 0.08 | 2154 (3.1) | | 19 (3.0) | |  |
| Breed | Retriever (Labrador) | 1.6 | [1.2, 2.1] | | | 0.001 | 10863 (15.7) | | 108 (16.8) | |  |
| Breed | Pug | 1.4 | [0.5, 3.9] | | | 0.48 | 795 (1.1) | | 7 (1.1) | |  |
| Breed | Cavalier King Charles Spaniel | 1.3 | [0.7, 2.5] | | | 0.33 | 1766 (2.5) | | 12 (1.9) | |  |
| Breed | Yorkshire Terrier | 1.3 | [0.8, 2.3] | | | 0.30 | 2167 (3.1) | | 17 (2.6) | |  |
| Breed | Weimaraner | 1.2 | [0.4, 3.8] | | | 0.77 | 396 (0.6) | | 6 (0.9) | |  |
| Breed | Crossbreed | 1 |  | | |  | 17602 (25.4) | | 103 (16.0) | |  |
| Breed | Spaniel (English Springer) | 0.9 | [0.6, 1.5] | | | 0.79 | 3896 (5.6) | | 27 (4.2) | |  |
| Breed | Jack Russell Terrier | 0.7 | [0.5, 1.1] | | | 0.12 | 6181 (8.9) | | 28 (4.3) | |  |
| Breed | West Highland White Terrier | 0.4 | [0.2, 0.9] | | | 0.02 | 2646 (3.8) | | 7 (1.1) | |  |
| Breed | Border Collie | 0.2 | [0.1, 0.5] | | | <0.001 | 4543 (6.5) | | 7 (1.1) | |  |
| Sex | Female neutered | 1 |  | | |  | 23840 (34.3) | | 226 (35.1) | |  |
| Sex | Female entire | 0.4 | [0.2, 0.9] | | | 0.03 | 11818 (17.0) | | 45 (7.0) | |  |
| Sex | Male neutered | 0.9 | [0.5, 1.6] | | | 0.78 | 20659 (29.8) | | 200 (31.1) | |  |
| Sex | Male entire | 0.7 | [0.3, 1.4] | | | 0.29 | 13092 (18.9) | | 104 (16.1) | |  |
| Age | [3,6) | 1 |  | | |  | 19475 (28.1) | | 74 (11.5) | |  |
| Age | [6,9) | 2.3 | [1.5, 3.6] | | | <0.001 | 18251 (26.3) | | 180 (28.0) | |  |
| Age | [9,12) | 4.1 | [2.7, 6.3] | | | <0.001 | 14967 (21.6) | | 262 (40.7) | |  |
| Age | [12,15) | 4.1 | [2.5, 6.7] | | | <0.001 | 8198 (11.8) | | 117 (18.2) | |  |
| Age | [15,18] | 2.9 | [1.1, 7.5] | | | 0.03 | 1611 (2.3) | | 10 (1.6) | |  |

## Supplementary table 11: emmeans for pairwise comparison between sex and neuter status for melanomas.

### Table 11.a: Oral melanomas

| **Comparison** | **OR** | **SE** | **Reference value** | **Z ratio** | **95% CI** | **p-value** |
| --- | --- | --- | --- | --- | --- | --- |
| FN - FE within [3,6) | 4.4 | 0,619 | 1 | 2,402 | [1.3, 14.9] | 0.076 |
| FN - MN within [3,6) | 0.8 | 0,307 | 1 | -0,721 | [0.4, 1.5] | 0.888 |
| FN - ME within [3,6) | 1.3 | 0,388 | 1 | 0,68 | [0.6, 2.8] | 0.905 |
| FE - MN within [3,6) | 0.2 | 0,613 | 1 | -2,787 | [0.1, 0.6] | 0.028 |
| FE - ME within [3,6) | 0.3 | 0,658 | 1 | -1,859 | [0.1, 1.1] | 0.246 |
| MN - ME within [3,6) | 1.6 | 0,38 | 1 | 1,277 | [0.8, 3.4] | 0.577 |
| FN - FE within [6,9) | 1.4 | 0,181 | 1 | 2,052 | [1.0, 2.1] | 0.170 |
| FN - MN within [6,9) | 0.6 | 0,118 | 1 | -3,738 | [0.5, 0.8] | 0.001 |
| FN - ME within [6,9) | 0.7 | 0,137 | 1 | -2,172 | [0.6, 1.0] | 0.131 |
| FE - MN within [6,9) | 0.4 | 0,176 | 1 | -4,617 | [0.3, 0.6] | <0.001 |
| FE - ME within [6,9) | 0.5 | 0,189 | 1 | -3,539 | [0.4, 0.7] | 0.002 |
| MN - ME within [6,9) | 1.2 | 0,131 | 1 | 1,096 | [0.9, 1.5] | 0.689 |
| FN - FE within [9,12) | 1.6 | 0,11 | 1 | 4,489 | [1.3, 2.0] | <0.001 |
| FN - MN within [9,12) | 0.6 | 0,069 | 1 | -7,193 | [0.5, 0.7] | <0.001 |
| FN - ME within [9,12) | 0.7 | 0,078 | 1 | -3,785 | [0.6, 0.9] | 0.001 |
| FE - MN within [9,12) | 0.4 | 0,108 | 1 | -9,168 | [0.3, 0.5] | <0.001 |
| FE - ME within [9,12) | 0.5 | 0,115 | 1 | -6,861 | [0.4, 0.6] | <0.001 |
| MN - ME within [9,12) | 1.2 | 0,075 | 1 | 2,681 | [1.1, 1.4] | 0.038 |
| FN - FE within [12,15) | 1.6 | 0,113 | 1 | 4,325 | [1.3, 2.0] | <0.001 |
| FN - MN within [12,15) | 0.6 | 0,072 | 1 | -7,223 | [0.5, 0.7] | <0.001 |
| FN - ME within [12,15) | 0.8 | 0,083 | 1 | -2,156 | [0.7, 1.0] | 0.140 |
| FE - MN within [12,15) | 0.4 | 0,113 | 1 | -8,928 | [0.3, 0.5] | <0.001 |
| FE - ME within [12,15) | 0.5 | 0,12 | 1 | -5,564 | [0.4, 0.6] | <0.001 |
| MN - ME within [12,15) | 1.4 | 0,083 | 1 | 4,11 | [1.2, 1.7] | 0.000 |
| FN - FE within [15,18] | 3.1 | 0,322 | 1 | 3,478 | [1.6, 5.8] | 0.003 |
| FN - MN within [15,18] | 0.7 | 0,178 | 1 | -2,312 | [0.5, 0.9] | 0.094 |
| FN - ME within [15,18] | 1.6 | 0,245 | 1 | 1,842 | [1.0, 2.5] | 0.253 |
| FE - MN within [15,18] | 0.2 | 0,322 | 1 | -4,756 | [0.1, 0.4] | <0.001 |
| FE - ME within [15,18] | 0.5 | 0,364 | 1 | -1,837 | [0.3, 1.0] | 0.255 |
| MN - ME within [15,18] | 2.4 | 0,245 | 1 | 3,522 | [1.5, 3.8] | 0.002 |

### Table 11.b: Cutaneous melanomas

| **Comparison** | **OR** | **SE** | **Reference value** | **Z ratio** | **CI95** | **p-value** |
| --- | --- | --- | --- | --- | --- | --- |
| FN - FE within [3,6) | 1.6 | 0,279 | 1 | 1,791 | [1.0, 2.8] | 0.278 |
| FN - MN within [3,6) | 0.7 | 0,197 | 1 | -1,786 | [0.5, 1.0] | 0.278 |
| FN - ME within [3,6) | 0.6 | 0,207 | 1 | -2,534 | [0.4, 0.9] | 0.054 |
| FE - MN within [3,6) | 0.4 | 0,271 | 1 | -3,142 | [0.3, 0.7] | 0.009 |
| FN - ME within [3,6) | 0.4 | 0,278 | 1 | -3,684 | [0.2, 0.6] | 0.001 |
| MN - ME within [3,6) | 0.8 | 0,195 | 1 | -0,886 | [0.6, 1.2] | 0.813 |
| FN - FE within [6,9) | 1.7 | 0,169 | 1 | 3,203 | [1.2, 2.4] | 0.008 |
| FN - MN within [6,9) | 0.7 | 0,105 | 1 | -4,088 | [0.5, 0.8] | 0.000 |
| FN - ME within [6,9) | 1 | 0,13 | 1 | -0,351 | [0.7, 1.2] | 0.985 |
| FE - MN within [6,9) | 0.4 | 0,166 | 1 | -5,847 | [0.3, 0.5] | <0.001 |
| FN - ME within [6,9) | 0.6 | 0,183 | 1 | -3,208 | [0.4, 0.8] | 0.007 |
| MN - ME within [6,9) | 1.5 | 0,126 | 1 | 3,045 | [1.1, 1.9] | 0.012 |
| FN - FE within [9,12) | 1.5 | 0,142 | 1 | 2,768 | [1.1, 2.0] | 0.029 |
| FN - MN within [9,12) | 0.7 | 0,092 | 1 | -3,95 | [0.6, 0.8] | 0.000 |
| FN - ME within [9,12) | 0.9 | 0,109 | 1 | -0,616 | [0.8, 1.2] | 0.928 |
| FE - MN within [9,12) | 0.5 | 0,141 | 1 | -5,365 | [0.4, 0.6] | <0.001 |
| FN - ME within [9,12) | 0.6 | 0,152 | 1 | -3,028 | [0.5, 0.9] | 0.013 |
| MN - ME within [9,12) | 1.3 | 0,107 | 1 | 2,768 | [1.1, 1.7] | 0.030 |
| FN - FE within [12,15) | 1.6 | 0,191 | 1 | 2,405 | [1.1, 2.3] | 0.077 |
| FN - MN within [12,15) | 0.7 | 0,124 | 1 | -2,44 | [0.6, 0.9] | 0.071 |
| FN - ME within [12,15) | 1 | 0,149 | 1 | 0,097 | [0.8, 1.4] | 1.000 |
| FE - MN within [12,15) | 0.5 | 0,193 | 1 | -3,948 | [0.3, 0.7] | 0.000 |
| FN - ME within [12,15) | 0.6 | 0,209 | 1 | -2,129 | [0.4, 1.0] | 0.145 |
| MN - ME within [12,15) | 1.4 | 0,151 | 1 | 2,099 | [1.0, 1.8] | 0.152 |
| FN - FE within [15,18] | 2.3 | 0,563 | 1 | 1,468 | [0.8, 6.9] | 0.457 |
| FN - MN within [15,18] | 0.8 | 0,362 | 1 | -0,635 | [0.4, 1.6] | 0.921 |
| FN - ME within [15,18] | 1.2 | 0,438 | 1 | 0,342 | [0.5, 2.7] | 0.986 |
| FE - MN within [15,18] | 0.3 | 0,57 | 1 | -1,853 | [0.1, 1.1] | 0.249 |
| FN - ME within [15,18] | 0.5 | 0,621 | 1 | -1,09 | [0.2, 1.7] | 0.696 |
| MN - ME within [15,18] | 1.5 | 0,447 | 1 | 0,849 | [0.6, 3.5] | 0.831 |

### Table 11.c: Cutaneous digital melanomas

| **Comparison** | **OR** | **SE** | **Reference value** | **Z ratio** | **95% CI** | **p-value** |
| --- | --- | --- | --- | --- | --- | --- |
| FN - FE within [3,6) | 2.1 | 0,569 | 1 | 1,27 | [0.7, 6.3] | 0.582 |
| FN - MN within [3,6) | 0.8 | 0,368 | 1 | -0,641 | [0.4, 1.6] | 0.919 |
| FN - ME within [3,6) | 0.7 | 0,388 | 1 | -0,976 | [0.3, 1.5] | 0.763 |
| FE - MN within [3,6) | 0.4 | 0,561 | 1 | -1,708 | [0.1, 1.2] | 0.319 |
| FE - ME within [3,6) | 0.3 | 0,574 | 1 | -1,918 | [0.1, 1.0] | 0.220 |
| MN - ME within [3,6) | 0.9 | 0,376 | 1 | -0,379 | [0.4, 1.8] | 0.981 |
| FN - FE within [6,9) | 1.5 | 0,271 | 1 | 1,538 | [0.9, 2.6] | 0.415 |
| FN - MN within [6,9) | 0.7 | 0,174 | 1 | -2,339 | [0.5, 0.9] | 0.090 |
| FN - ME within [6,9) | 0.7 | 0,196 | 1 | -1,851 | [0.5, 1.0] | 0.249 |
| FE - MN within [6,9) | 0.4 | 0,268 | 1 | -3,074 | [0.3, 0.7] | 0.011 |
| FE - ME within [6,9) | 0.5 | 0,282 | 1 | -2,765 | [0.3, 0.8] | 0.029 |
| MN - ME within [6,9) | 1 | 0,191 | 1 | 0,232 | [0.7, 1.5] | 0.996 |
| FN - FE within [9,12) | 2 | 0,23 | 1 | 3,08 | [1.3, 3.2] | 0.011 |
| FN - MN within [9,12) | 0.7 | 0,131 | 1 | -3,058 | [0.5, 0.9] | 0.012 |
| FN - ME within [9,12) | 1.2 | 0,163 | 1 | 0,945 | [0.8, 1.6] | 0.780 |
| FE - MN within [9,12) | 0.3 | 0,228 | 1 | -4,864 | [0.2, 0.5] | <0.001 |
| FE - ME within [9,12) | 0.6 | 0,247 | 1 | -2,244 | [0.4, 0.9] | 0.112 |
| MN - ME within [9,12) | 1.7 | 0,161 | 1 | 3,445 | [1.3, 2.4] | 0.003 |
| FN - FE within [12,15) | 2.6 | 0,364 | 1 | 2,637 | [1.3, 5.3] | 0.041 |
| FN - MN within [12,15) | 0.6 | 0,189 | 1 | -2,287 | [0.4, 0.9] | 0.102 |
| FN - ME within [12,15) | 1.2 | 0,239 | 1 | 0,623 | [0.7, 1.9] | 0.924 |
| FE - MN within [12,15) | 0.2 | 0,364 | 1 | -3,824 | [0.1, 0.5] | 0.001 |
| FE - ME within [12,15) | 0.4 | 0,392 | 1 | -2,068 | [0.2, 1.0] | 0.163 |
| MN - ME within [12,15) | 1.8 | 0,239 | 1 | 2,432 | [1.1, 2.9] | 0.072 |
| FN - FE within [15,18] | 0.4 | 0,782 | 1 | -1,066 | [0.1, 2.0] | 0.711 |
| FN - MN within [15,18] | 0.9 | 0,921 | 1 | -0,061 | [0.2, 5.7] | 1.000 |
| FN - ME within [15,18] | 97158.5 | 144,677 | 1 | 0,079 | [0.0, 2.3e+24] | 1.000 |
| FE - MN within [15,18] | 2.2 | 0,886 | 1 | 0,877 | [0.4, 12.3] | 0.817 |
| FE - ME within [15,18] | 223539.4 | 144,677 | 1 | 0,085 | [0.0, 2.3e+24] | 1.000 |
| MN - ME within [15,18] | 102794.9 | 144,678 | 1 | 0,08 | [0.0, 2.3e+24] | 1.000 |

### Table 11.d: Uveal melanomas

| **Comparison** | **OR** | **SE** | **Reference value** | **Z ratio** | **95% CI** | **p-value** |
| --- | --- | --- | --- | --- | --- | --- |
| FN - FE within [3,6) | 2,6 | 0,425 | 1 | 2,228 | [1.1, 5.9] | 0.116 |
| FN - MN within [3,6) | 1,1 | 0,283 | 1 | 0,275 | [0.6, 1.9] | 0.993 |
| FN - ME within [3,6) | 1,4 | 0,348 | 1 | 1,063 | [0.7, 2.9] | 0.712 |
| FE - MN within [3,6) | 0,4 | 0,434 | 1 | -2,002 | [0.2, 1.0] | 0.187 |
| FE - ME within [3,6) | 0,6 | 0,478 | 1 | -1,207 | [0.2, 1.4] | 0.622 |
| MN - ME within [3,6) | 1,3 | 0,359 | 1 | 0,814 | [0.7, 2.7] | 0.848 |
| FN - FE within [6,9) | 2,2 | 0,307 | 1 | 2,632 | [1.2, 4.1] | 0.042 |
| FN - MN within [6,9) | 1 | 0,186 | 1 | -0,202 | [0.7, 1.4] | 0.997 |
| FN - ME within [6,9) | 1,4 | 0,236 | 1 | 1,311 | [0.9, 2.2] | 0.555 |
| FE - MN within [6,9) | 0,4 | 0,311 | 1 | -2,719 | [0.2, 0.8] | 0.033 |
| FE - ME within [6,9) | 0,6 | 0,343 | 1 | -1,454 | [0.3, 1.2] | 0.465 |
| MN - ME within [6,9) | 1,4 | 0,241 | 1 | 1,439 | [0.9, 2.3] | 0.475 |
| FN - FE within [9,12) | 1,9 | 0,252 | 1 | 2,515 | [1.1, 3.1] | 0.058 |
| FN - MN within [9,12) | 0,8 | 0,157 | 1 | -1,283 | [0.6, 1.1] | 0.576 |
| FN - ME within [9,12) | 1,1 | 0,185 | 1 | 0,27 | [0.7, 1.5] | 0.993 |
| FE - MN within [9,12) | 0,4 | 0,253 | 1 | -3,301 | [0.3, 0.7] | 0.005 |
| FE - ME within [9,12) | 0,6 | 0,271 | 1 | -2,154 | [0.3, 0.9] | 0.136 |
| MN - ME within [9,12) | 1,3 | 0,186 | 1 | 1,351 | [0.9, 1.9] | 0.531 |
| FN - FE within [12,15) | 5,8 | 0,599 | 1 | 2,938 | [1.8, 18.8] | 0.017 |
| FN - MN within [12,15) | 0,8 | 0,227 | 1 | -1,002 | [0.5, 1.2] | 0.748 |
| FN - ME within [12,15) | 1,2 | 0,278 | 1 | 0,691 | [0.7, 2.1] | 0.901 |
| FE - MN within [12,15) | 0,1 | 0,603 | 1 | -3,295 | [0.0, 0.4] | 0.005 |
| FE - ME within [12,15) | 0,2 | 0,624 | 1 | -2,512 | [0.1, 0.7] | 0.058 |
| MN - ME within [12,15) | 1,5 | 0,286 | 1 | 1,467 | [0.9, 2.7] | 0.459 |
| FN - FE within [15,18] | 2,4 | 1,1 | 1 | 0,8 | [0.3, 20.8] | 0.855 |
| FN - MN within [15,18] | 1,7 | 0,842 | 1 | 0,628 | [0.3, 8.8] | 0.923 |
| FN - ME within [15,18] | 334021,3 | 226,067 | 1 | 0,056 | [0.0, 2.3e+24] | 1.000 |
| FE - MN within [15,18] | 0,7 | 1,23 | 1 | -0,286 | [0.1, 7.8] | 0.992 |
| FE - ME within [15,18] | 138588 | 226,068 | 1 | 0,052 | [0.0, 2.3e+24] | 1.000 |
| MN - ME within [15,18] | 196905,1 | 226,067 | 1 | 0,054 | [0.0, 2.3e+24] | 1.000 |

## Supplementary table 12: Anatomical distribution of Haemangiosarcomas

| Location | Count (n) | percent | Valid Proportion* |
| --- | --- | --- | --- |
| spleen | 5,262 | 46.7% | 47.7% |
| skin | 4,104 | 36.4% | 37.2% |
| liver and pancreas | 401 | 3.6% | 3.6% |
| retroperitoneum and peritoneum | 203 | 1.8% | 1.8% |
| oral cavity | 202 | 1.8% | 1.8% |
| muscle | 178 | 1.6% | 1.6% |
| eye and adnexa | 156 | 1.4% | 1.4% |
| lymph node | 129 | 1.1% | 1.2% |
| kidney and urinary tract | 77 | 0.7% | 0.7% |
| mammary gland | 67 | 0.6% | 0.6% |
| alimentary tract | 65 | 0.6% | 0.6% |
| accessory and paranasal sinuses | 35 | 0.3% | 0.3% |
| female reproductive system | 35 | 0.3% | 0.3% |
| heart, mediastinum and pleura | 34 | 0.3% | 0.3% |
| respiratory tract | 31 | 0.3% | 0.3% |
| gall and urinary bladder | 26 | 0.2% | 0.2% |
| nasal cavity | 12 | 0.1% | 0.1% |
| urinary system | 8 | 0.1% | 0.1% |
| thyroid gland | 6 | 0.1% | 0.1% |
| bone and joints | 5 | 0.0% | 0.0% |
| salivary glands | 2 | 0.0% | 0.0% |
| brain | 1 | 0.0% | 0.0% |
| nerve | 1 | 0.0% | 0.0% |
| NA* | 231 | 2.0% | - |
|  | | | |

## Supplementary table 13: Full data table from the multivariable logistic regression analysis for haemangiosarcomas.

## Table 13.a: Splenic haemangiosarcoma

| **Effect** | **Value** | **OR** | **95% CI** | **p-value** | **n (%) (Controls)** | **n (%) (Cases)** |
| --- | --- | --- | --- | --- | --- | --- |
| Breed | German Shepherd Dog | 7 | [5.9, 8.3] | <0.001 | 2154 (2.7) | 324 (13.6) |
| Breed | Retriever (Flat Coated) | 6.2 | [4.2, 9.3] | <0.001 | 301 (0.4) | 37 (1.6) |
| Breed | Hungarian Vizsla | 5.6 | [3.7, 8.5] | <0.001 | 358 (0.4) | 29 (1.2) |
| Breed | Italian Spinone | 5.5 | [2.4, 12.4] | <0.001 | 87 (0.1) | 8 (0.3) |
| Breed | Belgian Shepherd Dog | 5.1 | [2.0, 13.3] | <0.001 | 66 (0.1) | 5 (0.2) |
| Breed | Briard | 5 | [1.4, 17.4] | 0.01 | 31 (0.0) | 6 (0.3) |
| Breed | Dogue De Bordeaux | 5 | [2.0, 12.9] | <0.001 | 128 (0.2) | 6 (0.3) |
| Breed | Collie (Rough) | 4.4 | [2.9, 6.9] | <0.001 | 274 (0.3) | 26 (1.1) |
| Breed | Soft Coated Wheaten Terrier | 4.2 | [1.6, 10.8] | 0.00 | 59 (0.1) | 5 (0.2) |
| Breed | Irish Terrier | 3.4 | [1.3, 8.6] | 0.01 | 72 (0.1) | 5 (0.2) |
| Breed | Spaniel (Generic) | 3.2 | [1.6, 6.5] | 0.00 | 110 (0.1) | 9 (0.4) |
| Breed | Miniature Schnauzer | 3 | [2.2, 4.1] | <0.001 | 867 (1.1) | 55 (2.3) |
| Breed | German Shorthaired Pointer | 2.8 | [1.7, 4.6] | <0.001 | 304 (0.4) | 17 (0.7) |
| Breed | Bullmastiff | 2.7 | [1.0, 7.5] | 0.06 | 112 (0.1) | 7 (0.3) |
| Breed | Shar Pei | 2.5 | [1.0, 6.3] | 0.05 | 174 (0.2) | 6 (0.3) |
| Breed | Retriever (Labrador) | 2.4 | [2.1, 2.7] | <0.001 | 10862 (13.6) | 607 (25.5) |
| Breed | Bulldog | 2.3 | [1.2, 4.5] | 0.01 | 493 (0.6) | 10 (0.4) |
| Breed | Schnauzer | 2.2 | [1.0, 4.5] | 0.04 | 198 (0.2) | 9 (0.4) |
| Breed | Retriever (Generic) | 2.1 | [1.2, 3.8] | 0.02 | 208 (0.3) | 14 (0.6) |
| Breed | Beagle | 1.8 | [1.1, 2.8] | 0.02 | 658 (0.8) | 21 (0.9) |
| Breed | Boxer | 1.8 | [1.3, 2.6] | <0.001 | 1079 (1.4) | 47 (2.0) |
| Breed | Rhodesian Ridgeback | 1.8 | [0.8, 3.8] | 0.15 | 218 (0.3) | 7 (0.3) |
| Breed | Tibetan Terrier | 1.8 | [1.0, 3.4] | 0.06 | 255 (0.3) | 11 (0.5) |
| Breed | Irish Setter | 1.7 | [0.8, 3.5] | 0.15 | 223 (0.3) | 8 (0.3) |
| Breed | Labradoodle | 1.7 | [1.0, 2.9] | 0.05 | 686 (0.9) | 15 (0.6) |
| Breed | Retriever (Golden) | 1.6 | [1.3, 2.1] | <0.001 | 1911 (2.4) | 79 (3.3) |
| Breed | Airedale Terrier | 1.4 | [0.6, 3.5] | 0.45 | 146 (0.2) | 5 (0.2) |
| Breed | Border Collie | 1.2 | [1.0, 1.5] | 0.08 | 4541 (5.7) | 129 (5.4) |
| Breed | Rottweiler | 1.2 | [0.7, 2.1] | 0.59 | 580 (0.7) | 15 (0.6) |
| Breed | Siberian Husky | 1.2 | [0.6, 2.3] | 0.66 | 441 (0.6) | 11 (0.5) |
| Breed | Staffordshire Bull Terrier | 1.2 | [0.9, 1.5] | 0.21 | 3081 (3.9) | 88 (3.7) |
| Breed | Weimaraner | 1.2 | [0.7, 2.2] | 0.48 | 396 (0.5) | 16 (0.7) |
| Breed | Crossbreed | 1 |  |  | 17602 (22.0) | 404 (17.0) |
| Breed | Bichon Frise | 0.9 | [0.6, 1.5] | 0.80 | 1029 (1.3) | 24 (1.0) |
| Breed | Lurcher | 0.9 | [0.6, 1.4] | 0.68 | 1205 (1.5) | 24 (1.0) |
| Breed | Scottish Terrier | 0.9 | [0.4, 2.1] | 0.75 | 221 (0.3) | 5 (0.2) |
| Breed | Spaniel (Cocker) | 0.9 | [0.7, 1.2] | 0.61 | 5384 (6.7) | 99 (4.2) |
| Breed | Dachshund (Standard) | 0.8 | [0.4, 1.6] | 0.53 | 727 (0.9) | 9 (0.4) |
| Breed | Bearded Collie | 0.7 | [0.3, 1.9] | 0.46 | 271 (0.3) | 5 (0.2) |
| Breed | Cockapoo | 0.7 | [0.2, 1.8] | 0.42 | 881 (1.1) | 6 (0.3) |
| Breed | Cairn Terrier | 0.6 | [0.3, 1.2] | 0.15 | 516 (0.6) | 7 (0.3) |
| Breed | Terrier (Generic) | 0.6 | [0.3, 1.0] | 0.07 | 809 (1.0) | 12 (0.5) |
| Breed | Dalmatian | 0.5 | [0.2, 1.3] | 0.15 | 397 (0.5) | 5 (0.2) |
| Breed | Dachshund (Miniature) | 0.4 | [0.2, 1.2] | 0.10 | 486 (0.6) | 5 (0.2) |
| Breed | Chihuahua | 0.3 | [0.1, 0.7] | 0.01 | 1085 (1.4) | 5 (0.2) |
| Breed | Greyhound | 0.3 | [0.2, 0.7] | 0.001 | 1253 (1.6) | 9 (0.4) |
| Breed | Jack Russell Terrier | 0.3 | [0.2, 0.5] | <0.001 | 6181 (7.7) | 48 (2.0) |
| Breed | Spaniel (English Springer) | 0.3 | [0.2, 0.5] | <0.001 | 3896 (4.9) | 30 (1.3) |
| Breed | West Highland White Terrier | 0.3 | [0.2, 0.5] | <0.001 | 2646 (3.3) | 21 (0.9) |
| Breed | Yorkshire Terrier | 0.3 | [0.1, 0.5] | <0.001 | 2166 (2.7) | 14 (0.6) |
| Breed | Border Terrier | 0.2 | [0.1, 0.4] | <0.001 | 2030 (2.5) | 11 (0.5) |
| Sex | Female neutered | 1 |  |  | 27403 (34.3) | 740 (31.1) |
| Sex | Female entire | 0.4 | [0.1, 1.4] | 0.16 | 13535 (16.9) | 171 (7.2) |
| Sex | Male neutered | 1.5 | [0.7, 3.1] | 0.32 | 24084 (30.2) | 885 (37.2) |
| Sex | Male entire | 0.5 | [0.2, 1.7] | 0.29 | 14836 (18.6) | 355 (14.9) |
| Age | [3,6) | 1 |  |  | 22771 (28.5) | 38 (1.6) |
| Age | [6,9) | 12.5 | [6.9, 22.4] | <0.001 | 20854 (26.1) | 610 (25.6) |
| Age | [9,12) | 42.6 | [24.0, 75.9] | <0.001 | 16977 (21.3) | 1314 (55.2) |
| Age | [12,15) | 29.2 | [16.2, 52.7] | <0.001 | 9328 (11.7) | 405 (17.0) |
| Age | [15,18] | 6.4 | [2.2, 18.3] | <0.001 | 1869 (2.3) | 13 (0.5) |

## Table 13.b: Cutaneous haemangiosarcoma

| **Effect** | **Value** | **OR** | **95% CI** | **p-value** | **n (%) (Controls)** | **n (%) (Cases)** |
| --- | --- | --- | --- | --- | --- | --- |
| Breed | Mastiff | 20.8 | [10.6, 40.9] | <0.001 | 53 (0.1) | 12 (0.7) |
| Breed | Bullmastiff | 14.2 | [8.4, 23.9] | <0.001 | 112 (0.1) | 23 (1.3) |
| Breed | Dogue De Bordeaux | 13.3 | [7.4, 24.0] | <0.001 | 128 (0.2) | 15 (0.8) |
| Breed | Bulldog | 5.9 | [3.9, 9.1] | <0.001 | 493 (0.6) | 27 (1.5) |
| Breed | Boxer | 5.4 | [4.2, 6.9] | <0.001 | 1079 (1.3) | 103 (5.7) |
| Breed | Italian Spinone | 5.2 | [2.2, 12.3] | <0.001 | 87 (0.1) | 6 (0.3) |
| Breed | Beagle | 4.2 | [3.0, 5.9] | <0.001 | 658 (0.8) | 43 (2.4) |
| Breed | English Pointer | 3.5 | [1.9, 6.2] | <0.001 | 237 (0.3) | 13 (0.7) |
| Breed | German Shepherd Dog | 3.3 | [2.6, 4.1] | <0.001 | 2154 (2.6) | 126 (6.9) |
| Breed | Collie (Rough) | 2.8 | [1.6, 4.9] | <0.001 | 274 (0.3) | 14 (0.8) |
| Breed | Alaskan Malamute | 2.6 | [1.0, 6.5] | 0.04 | 144 (0.2) | 6 (0.3) |
| Breed | Hungarian Vizsla | 2.6 | [1.4, 4.7] | 0.001 | 358 (0.4) | 15 (0.8) |
| Breed | Retriever (Flat Coated) | 2.5 | [1.3, 4.6] | 0.001 | 301 (0.4) | 11 (0.6) |
| Breed | Whippet | 2.3 | [1.6, 3.5] | <0.001 | 770 (0.9) | 30 (1.6) |
| Breed | Retriever (Golden) | 2.2 | [1.7, 2.8] | <0.001 | 1912 (2.3) | 81 (4.5) |
| Breed | French Bulldog | 2.1 | [1.0, 4.3] | 0.04 | 577 (0.7) | 12 (0.7) |
| Breed | Greyhound | 2.1 | [1.5, 2.9] | <0.001 | 1253 (1.5) | 51 (2.8) |
| Breed | Poodle (Generic) | 2.1 | [1.1, 4.1] | 0.03 | 264 (0.3) | 9 (0.5) |
| Breed | Lurcher | 2 | [1.4, 2.7] | <0.001 | 1205 (1.5) | 46 (2.5) |
| Breed | Staffordshire Bull Terrier | 1.8 | [1.4, 2.3] | <0.001 | 3081 (3.7) | 108 (5.9) |
| Breed | Bichon Frise | 1.7 | [1.2, 2.5] | 0.00 | 1029 (1.2) | 33 (1.8) |
| Breed | Spaniel (Generic) | 1.7 | [0.6, 4.7] | 0.29 | 110 (0.1) | 5 (0.3) |
| Breed | Bedlington Terrier | 1.3 | [0.5, 3.6] | 0.61 | 173 (0.2) | 5 (0.3) |
| Breed | German Shorthaired Pointer | 1.3 | [0.6, 2.9] | 0.46 | 304 (0.4) | 7 (0.4) |
| Breed | Labradoodle | 1.3 | [0.7, 2.4] | 0.36 | 686 (0.8) | 12 (0.7) |
| Breed | Retriever (Generic) | 1.3 | [0.6, 3.0] | 0.49 | 208 (0.3) | 9 (0.5) |
| Breed | Rottweiler | 1.3 | [0.7, 2.3] | 0.46 | 579 (0.7) | 15 (0.8) |
| Breed | Weimaraner | 1.3 | [0.7, 2.5] | 0.36 | 396 (0.5) | 11 (0.6) |
| Breed | Dobermann | 1.2 | [0.5, 3.0] | 0.65 | 283 (0.3) | 5 (0.3) |
| Breed | Siberian Husky | 1.2 | [0.6, 2.5] | 0.62 | 441 (0.5) | 13 (0.7) |
| Breed | Tibetan Terrier | 1.2 | [0.5, 2.8] | 0.65 | 255 (0.3) | 7 (0.4) |
| Breed | Pug | 1.1 | [0.6, 2.1] | 0.79 | 795 (1.0) | 10 (0.5) |
| Breed | Retriever (Labrador) | 1.1 | [0.9, 1.3] | 0.33 | 10863 (13.1) | 239 (13.1) |
| Breed | Shetland Sheepdog | 1.1 | [0.5, 2.8] | 0.80 | 237 (0.3) | 7 (0.4) |
| Breed | Crossbreed | 1 |  |  | 17601 (21.3) | 339 (18.6) |
| Breed | Dachshund (Standard) | 1 | [0.5, 1.9] | 0.95 | 727 (0.9) | 13 (0.7) |
| Breed | Irish Setter | 1 | [0.4, 2.9] | 0.93 | 223 (0.3) | 6 (0.3) |
| Breed | Border Collie | 0.7 | [0.5, 0.9] | 0.02 | 4543 (5.5) | 63 (3.5) |
| Breed | Cockapoo | 0.7 | [0.3, 1.7] | 0.46 | 881 (1.1) | 6 (0.3) |
| Breed | Dachshund (Miniature) | 0.7 | [0.3, 1.7] | 0.49 | 486 (0.6) | 6 (0.3) |
| Breed | Jack Russell Terrier | 0.7 | [0.5, 0.9] | 0.00 | 6181 (7.5) | 87 (4.8) |
| Breed | Dalmatian | 0.6 | [0.3, 1.5] | 0.31 | 397 (0.5) | 5 (0.3) |
| Breed | Miniature Schnauzer | 0.6 | [0.3, 1.2] | 0.19 | 867 (1.0) | 11 (0.6) |
| Breed | West Highland White Terrier | 0.6 | [0.4, 0.8] | 0.001 | 2646 (3.2) | 35 (1.9) |
| Breed | Patterdale Terrier | 0.5 | [0.2, 1.3] | 0.18 | 583 (0.7) | 5 (0.3) |
| Breed | Spaniel (Cocker) | 0.5 | [0.4, 0.7] | <0.001 | 5384 (6.5) | 46 (2.5) |
| Breed | Spaniel (English Springer) | 0.5 | [0.3, 0.7] | <0.001 | 3896 (4.7) | 36 (2.0) |
| Breed | Terrier (Generic) | 0.4 | [0.2, 0.9] | 0.03 | 809 (1.0) | 7 (0.4) |
| Breed | Border Terrier | 0.3 | [0.2, 0.5] | <0.001 | 2030 (2.5) | 11 (0.6) |
| Breed | Cavalier King Charles Spaniel | 0.2 | [0.1, 0.5] | <0.001 | 1766 (2.1) | 6 (0.3) |
| Breed | Yorkshire Terrier | 0.2 | [0.1, 0.4] | <0.001 | 2167 (2.6) | 9 (0.5) |
| Sex | Female neutered | 1 |  |  | 28290 (34.2) | 565 (31.0) |
| Sex | Female entire | 0.7 | [0.4, 1.2] | 0.17 | 14152 (17.1) | 123 (6.8) |
| Sex | Male neutered | 1.6 | [1.1, 2.3] | 0.02 | 24764 (29.9) | 683 (37.5) |
| Sex | Male entire | 1.5 | [1.0, 2.3] | 0.07 | 15480 (18.7) | 288 (15.8) |
| Age | [3,6) | 1 |  |  | 23814 (28.8) | 188 (10.3) |
| Age | [6,9) | 3 | [2.1, 4.2] | <0.001 | 21683 (26.2) | 529 (29.1) |
| Age | [9,12) | 7.2 | [5.2, 9.9] | <0.001 | 17416 (21.1) | 744 (40.9) |
| Age | [12,15) | 8.2 | [5.8, 11.6] | <0.001 | 9390 (11.4) | 329 (18.1) |
| Age | [15,18] | 3.2 | [1.5, 6.6] | 0.001 | 1854 (2.2) | 21 (1.2) |

## Supplementary table 14: emmeans for pairwise comparison between sex and neuter status for haemangiosarcomas.

### Table 14.a: Splenic haemangiosarcoma

| **Comparison** | **OR** | **SE** | **Reference value** | | **Z ratio** | | **95% CI** | | **p-value** | |
| --- | --- | --- | --- | --- | --- | --- | --- | --- | --- | --- |
| FN - FE within [3,6) | 2.5 | 0,646 | 1 | 1,389 | | [0.7, 8.7] | | 0.506 | |  |
| FN - MN within [3,6) | 0.7 | 0,383 | 1 | -0,988 | | [0.3, 1.5] | | 0.755 | |  |
| FN - ME within [3,6) | 1.8 | 0,578 | 1 | 1,06 | | [0.6, 5.7] | | 0.714 | |  |
| FE - MN within [3,6) | 0.3 | 0,63 | 1 | -2,025 | | [0.1, 1.0] | | 0.178 | |  |
| FE - ME within [3,6) | 0.8 | 0,764 | 1 | -0,373 | | [0.2, 3.4] | | 0.982 | |  |
| MN - ME within [3,6) | 2.7 | 0,56 | 1 | 1,77 | | [0.9, 8.1] | | 0.288 | |  |
| FN - FE within [6,9) | 1.7 | 0,177 | 1 | 2,886 | | [1.2, 2.4] | | 0.020 | |  |
| FN - MN within [6,9) | 0.5 | 0,106 | 1 | -5,816 | | [0.4, 0.7] | | <0.001 | |  |
| FN - ME within [6,9) | 0.8 | 0,131 | 1 | -1,579 | | [0.6, 1.1] | | 0.391 | |  |
| FE - MN within [6,9) | 0.3 | 0,17 | 1 | -6,631 | | [0.2, 0.5] | | <0.001 | |  |
| FE - ME within [6,9) | 0.5 | 0,187 | 1 | -3,837 | | [0.3, 0.7] | | 0.001 | |  |
| MN - ME within [6,9) | 1.5 | 0,122 | 1 | 3,358 | | [1.2, 1.9] | | 0.004 | |  |
| FN - FE within [9,12) | 1.8 | 0,116 | 1 | 5,027 | | [1.4, 2.2] | | <0.001 | |  |
| FN - MN within [9,12) | 0.7 | 0,071 | 1 | -5,383 | | [0.6, 0.8] | | <0.001 | |  |
| FN - ME within [9,12) | 1.2 | 0,092 | 1 | 2,416 | | [1.0, 1.5] | | 0.076 | |  |
| FE - MN within [9,12) | 0.4 | 0,114 | 1 | -8,468 | | [0.3, 0.5] | | <0.001 | |  |
| FE - ME within [9,12) | 0.7 | 0,128 | 1 | -2,819 | | [0.5, 0.9] | | 0.026 | |  |
| MN - ME within [9,12) | 1.8 | 0,091 | 1 | 6,643 | | [1.5, 2.2] | | <0.001 | |  |
| FN - FE within [12,15) | 2.3 | 0,22 | 1 | 3,772 | | [1.5, 3.5] | | 0.001 | |  |
| FN - MN within [12,15) | 0.8 | 0,125 | 1 | -2,111 | | [0.6, 1.0] | | 0.150 | |  |
| FN - ME within [12,15) | 1.2 | 0,158 | 1 | 1,375 | | [0.9, 1.7] | | 0.514 | |  |
| FE - MN within [12,15) | 0.3 | 0,223 | 1 | -4,905 | | [0.2, 0.5] | | <0.001 | |  |
| FE - ME within [12,15) | 0.5 | 0,243 | 1 | -2,521 | | [0.3, 0.9] | | 0.056 | |  |
| MN - ME within [12,15) | 1.6 | 0,162 | 1 | 2,97 | | [1.2, 2.2] | | 0.015 | |  |
| FN - FE within [15,18] | 112797.8 | 127,862 | 1 | 0,091 | | [0.0, 2.3e+24] | | 1.000 | |  |
| FN - MN within [15,18] | 0.4 | 0,575 | 1 | -1,599 | | [0.1, 1.2] | | 0.379 | |  |
| FN - ME within [15,18] | 98307.1 | 123,121 | 1 | 0,093 | | [0.0, 2.3e+24] | | 1.000 | |  |
| FE - MN within [15,18] | 0 | 127,862 | 1 | -0,098 | | [0.0, 2.3e+24] | | 1.000 | |  |
| FE - ME within [15,18] | 0.9 | 177,502 | 1 | -0,001 | | [0.0, 2.3e+24] | | 1.000 | |  |
| MN - ME within [15,18] | 246555.3 | 123,12 | 1 | 0,101 | | [0.0, 2.3e+24] | | 1.000 | |  |

### Table 14.b: Cutaneous haemangiosarcoma

| **Comparison** | **OR** | **SE** | **Reference value** | **Z ratio** | **95% CI** | **p-value** |  |
| --- | --- | --- | --- | --- | --- | --- | --- |
| FN - FE within [3,6) | 1.5 | 0,275 | 1 | 1,388 | [0.9, 2.5] | 0.508 |  |
| FN - MN within [3,6) | 0.6 | 0,197 | 1 | -2,263 | [0.4, 0.9] | 0.106 | |
| FN - ME within [3,6) | 0.7 | 0,218 | 1 | -1,786 | [0.4, 1.0] | 0.282 |  |
| FE - MN within [3,6) | 0.4 | 0,264 | 1 | -3,135 | [0.3, 0.7] | 0.009 |  |
| FE - ME within [3,6) | 0.5 | 0,28 | 1 | -2,754 | [0.3, 0.8] | 0.030 |  |
| MN - ME within [3,6) | 1.1 | 0,204 | 1 | 0,277 | [0.7, 1.6] | 0.993 |  |
| FN - FE within [6,9) | 1.7 | 0,191 | 1 | 2,777 | [1.2, 2.5] | 0.028 |  |
| FN - MN within [6,9) | 0.5 | 0,113 | 1 | -6,32 | [0.4, 0.6] | <0.001 |  |
| FN - ME within [6,9) | 0.9 | 0,146 | 1 | -0,751 | [0.7, 1.2] | 0.875 |  |
| FE - MN within [6,9) | 0.3 | 0,182 | 1 | -6,839 | [0.2, 0.4] | <0.001 |  |
| FE - ME within [6,9) | 0.5 | 0,204 | 1 | -3,138 | [0.4, 0.8] | 0.009 |  |
| MN - ME within [6,9) | 1.8 | 0,134 | 1 | 4,511 | [1.4, 2.4] | <0.001 |  |
| FN - FE within [9,12) | 2.5 | 0,172 | 1 | 5,36 | [1.8, 3.5] | <0.001 |  |
| FN - MN within [9,12) | 0.7 | 0,092 | 1 | -3,494 | [0.6, 0.9] | 0.003 |  |
| FN - ME within [9,12) | 1.1 | 0,114 | 1 | 0,45 | [0.8, 1.3] | 0.969 |  |
| FE - MN within [9,12) | 0.3 | 0,171 | 1 | -7,271 | [0.2, 0.4] | <0.001 |  |
| FE - ME within [9,12) | 0.4 | 0,183 | 1 | -4,758 | [0.3, 0.6] | <0.001 |  |
| MN - ME within [9,12) | 1.5 | 0,112 | 1 | 3,328 | [1.2, 1.8] | 0.005 |  |
| FN - FE within [12,15) | 2.1 | 0,217 | 1 | 3,326 | [1.3, 3.1] | 0.005 |  |
| FN - MN within [12,15) | 0.9 | 0,132 | 1 | -0,87 | [0.7, 1.2] | 0.820 |  |
| FN - ME within [12,15) | 1.9 | 0,189 | 1 | 3,431 | [1.3, 2.8] | 0.003 |  |
| FE - MN within [12,15) | 0.4 | 0,222 | 1 | -3,768 | [0.3, 0.7] | 0.001 |  |
| FE - ME within [12,15) | 0.9 | 0,26 | 1 | -0,282 | [0.6, 1.5] | 0.992 |  |
| MN - ME within [12,15) | 2.1 | 0,194 | 1 | 3,934 | [1.5, 3.1] | 0.000 |  |
| FN - FE within [15,18] | 4.5 | 1,057 | 1 | 1,43 | [0.6, 36.0] | 0.481 |  |
| FN - MN within [15,18] | 0.8 | 0,49 | 1 | -0,497 | [0.3, 2.0] | 0.960 |  |
| FN - ME within [15,18] | 2.3 | 0,785 | 1 | 1,061 | [0.5, 10.7] | 0.713 |  |
| FE - MN within [15,18] | 0.2 | 1,064 | 1 | -1,65 | [0.0, 1.4] | 0.351 |  |
| FE - ME within [15,18] | 0.5 | 1,228 | 1 | -0,552 | [0.0, 5.6] | 0.946 |  |
| MN - ME within [15,18] | 2.9 | 0,795 | 1 | 1,355 | [0.6, 13.9] | 0.528 |  |

## Supplementary table 15: Anatomical distribution of all osteosarcomas.

| Location | Count (n) | Valid Proportion* |
| --- | --- | --- |
| oral cavity | 1,091 | 23.4% |
| hindlimb | 648 | 13.9% |
| forelimb | 593 | 12.7% |
| mammary gland | 431 | 9.3% |
| bone NOS* | 313 | 6.7% |
| thorax | 240 | 5.2% |
| head and face | 207 | 4.4% |
| carpus and metacarpus | 202 | 4.3% |
| lymph node | 101 | 2.2% |
| eye and adnexa | 98 | 2.1% |
| alimentary tract | 77 | 1.7% |
| muscle | 77 | 1.7% |
| respiratory tract | 73 | 1.6% |
| nasal cavity | 59 | 1.3% |
| abdominal and inguinal region | 45 | 1.0% |
| skin NOS* | 45 | 1.0% |
| pelvic region | 43 | 0.9% |
| spleen | 42 | 0.9% |
| limb | 30 | 0.6% |
| accessory and paranasal sinuses | 28 | 0.6% |
| neck | 28 | 0.6% |
| anal and perianal region | 25 | 0.5% |
| trunk | 21 | 0.5% |
| paws | 19 | 0.4% |
| ear | 18 | 0.4% |
| joint | 15 | 0.3% |
| female reproductive system | 13 | 0.3% |
| tarsus and metatarsus | 13 | 0.3% |
| brain | 11 | 0.2% |
| tail | 8 | 0.2% |
| heart, mediastinum and pleura | 6 | 0.1% |
| kidney and urinary tract | 6 | 0.1% |
| liver and pancreas | 6 | 0.1% |
| retroperitoneum and peritoneum | 5 | 0.1% |

## Supplementary table 16: Anatomical distribution of Osteosarcomas in high-odds breeds.

| Location in high-risk breeds | Count (n) | Valid Proportion* |
| --- | --- | --- |
| hindlimb | 193 | 25.0% |
| forelimb | 152 | 19.7% |
| oral cavity | 112 | 14.5% |
| bone NOS* | 49 | 6.3% |
| thorax | 43 | 5.6% |
| carpus and metacarpus | 33 | 4.3% |
| head and face | 23 | 3.0% |
| respiratory tract | 23 | 3.0% |
| lymph node | 21 | 2.7% |
| mammary gland | 16 | 2.1% |
| muscle | 16 | 2.1% |
| alimentary tract | 14 | 1.8% |
| eye and adnexa | 13 | 1.7% |
| limb | 10 | 1.3% |
| accessory and paranasal sinuses | 7 | 0.9% |
| abdominal and inguinal region | 6 | 0.8% |
| anal and perianal region | 6 | 0.8% |
| nasal cavity | 5 | 0.6% |
| pelvic region | 5 | 0.6% |
| skin NOS* | 5 | 0.6% |

## Supplementary table 17: Anatomical distribution of Osteosarcomas in low-odds breeds.

| Location in low-risk breeds | Count (n) | Valid Proportion* |
| --- | --- | --- |
| oral cavity | 123 | 25.2% |
| mammary gland | 82 | 16.8% |
| hindlimb | 41 | 8.4% |
| forelimb | 35 | 7.2% |
| head and face | 34 | 7.0% |
| bone NOS* | 24 | 4.9% |
| carpus and metacarpus | 22 | 4.5% |
| thorax | 22 | 4.5% |
| lymph node | 14 | 2.9% |
| eye and adnexa | 12 | 2.5% |
| spleen | 11 | 2.2% |
| nasal cavity | 10 | 2.0% |
| abdominal and inguinal region | 7 | 1.4% |
| alimentary tract | 7 | 1.4% |
| respiratory tract | 6 | 1.2% |
| skin NOS* | 6 | 1.2% |
| accessory and paranasal sinuses | 5 | 1.0% |
| muscle | 5 | 1.0% |
| neck | 5 | 1.0% |

## Supplementary table 18: Full data table from the multivariable logistic regression analysis for osteosarcomas.

| **Effect** | **Value** | **OR** | **95% CI** | **p-value** | **n (%) (Controls)** | **n (%) (Cases)** |
| --- | --- | --- | --- | --- | --- | --- |
| Breed | Leonberger | 17.2 | [8.6, 34.2] | <0.001 | 47 (0.1) | 12 (0.6) |
| Breed | Mastiff | 13.6 | [6.7, 27.4] | <0.001 | 53 (0.1) | 13 (0.6) |
| Breed | Rottweiler | 10.6 | [8.4, 13.3] | <0.001 | 580 (0.7) | 131 (6.0) |
| Breed | Bullmastiff | 9.6 | [5.6, 16.6] | <0.001 | 112 (0.1) | 18 (0.8) |
| Breed | Deerhound | 8.2 | [3.4, 20.1] | <0.001 | 54 (0.1) | 6 (0.3) |
| Breed | St. Bernard | 6.6 | [2.5, 17.1] | <0.001 | 64 (0.1) | 6 (0.3) |
| Breed | Dobermann | 6.3 | [4.3, 9.4] | <0.001 | 283 (0.3) | 39 (1.8) |
| Breed | Italian Spinone | 5.4 | [2.5, 11.6] | <0.001 | 87 (0.1) | 9 (0.4) |
| Breed | Old English Sheepdog | 5 | [2.4, 10.1] | <0.001 | 89 (0.1) | 9 (0.4) |
| Breed | Rhodesian Ridgeback | 4.6 | [2.8, 7.4] | <0.001 | 218 (0.3) | 21 (1.0) |
| Breed | Great Dane | 4.4 | [2.3, 8.4] | <0.001 | 154 (0.2) | 12 (0.6) |
| Breed | Bernese Mountain Dog | 4.3 | [1.9, 9.4] | <0.001 | 116 (0.1) | 7 (0.3) |
| Breed | Greyhound | 4.2 | [3.4, 5.2] | <0.001 | 1253 (1.5) | 133 (6.1) |
| Breed | Belgian Shepherd Dog | 4 | [1.6, 10.2] | 0.00 | 66 (0.1) | 7 (0.3) |
| Breed | Collie (Rough) | 4 | [2.6, 6.2] | <0.001 | 274 (0.3) | 24 (1.1) |
| Breed | Bulldog | 3 | [1.8, 5.1] | <0.001 | 493 (0.6) | 22 (1.0) |
| Breed | French Bulldog | 2.9 | [1.7, 5.2] | <0.001 | 577 (0.7) | 19 (0.9) |
| Breed | Spaniel (Generic) | 2.8 | [1.3, 5.8] | 0.01 | 110 (0.1) | 9 (0.4) |
| Breed | Bull Terrier | 2.7 | [1.5, 4.8] | <0.001 | 231 (0.3) | 16 (0.7) |
| Breed | Retriever (Flat Coated) | 2.7 | [1.6, 4.6] | <0.001 | 301 (0.4) | 17 (0.8) |
| Breed | Lurcher | 2.5 | [1.9, 3.2] | <0.001 | 1205 (1.4) | 71 (3.3) |
| Breed | Hungarian Vizsla | 2.2 | [1.2, 3.9] | 0.01 | 358 (0.4) | 14 (0.6) |
| Breed | Akita | 2.1 | [0.9, 5.3] | 0.11 | 143 (0.2) | 7 (0.3) |
| Breed | Boxer | 1.8 | [1.3, 2.5] | <0.001 | 1079 (1.3) | 46 (2.1) |
| Breed | Staffordshire Bull Terrier | 1.8 | [1.5, 2.2] | <0.001 | 3081 (3.7) | 148 (6.8) |
| Breed | Fox Terrier | 1.6 | [0.7, 3.5] | 0.23 | 194 (0.2) | 7 (0.3) |
| Breed | Retriever (Generic) | 1.6 | [0.8, 3.1] | 0.19 | 208 (0.3) | 11 (0.5) |
| Breed | Weimaraner | 1.5 | [0.9, 2.5] | 0.12 | 396 (0.5) | 17 (0.8) |
| Breed | Siberian Husky | 1.4 | [0.8, 2.6] | 0.23 | 441 (0.5) | 13 (0.6) |
| Breed | English Pointer | 1.3 | [0.6, 2.9] | 0.59 | 237 (0.3) | 6 (0.3) |
| Breed | Labradoodle | 1.3 | [0.8, 2.3] | 0.30 | 686 (0.8) | 15 (0.7) |
| Breed | German Shepherd Dog | 1.2 | [0.9, 1.6] | 0.31 | 2154 (2.6) | 64 (3.0) |
| Breed | Retriever (Labrador) | 1.2 | [1.0, 1.4] | 0.05 | 10863 (13.1) | 317 (14.6) |
| Breed | German Shorthaired Pointer | 1.1 | [0.5, 2.3] | 0.89 | 304 (0.4) | 8 (0.4) |
| Breed | Retriever (Golden) | 1.1 | [0.8, 1.5] | 0.63 | 1912 (2.3) | 59 (2.7) |
| Breed | Tibetan Terrier | 1.1 | [0.5, 2.4] | 0.75 | 255 (0.3) | 7 (0.3) |
| Breed | Crossbreed | 1 |  |  | 17601 (21.2) | 431 (19.9) |
| Breed | Scottish Terrier | 1 | [0.5, 2.4] | 0.93 | 221 (0.3) | 6 (0.3) |
| Breed | Beagle | 0.8 | [0.4, 1.5] | 0.52 | 658 (0.8) | 10 (0.5) |
| Breed | Dalmatian | 0.8 | [0.4, 1.6] | 0.53 | 397 (0.5) | 8 (0.4) |
| Breed | Irish Setter | 0.8 | [0.3, 2.3] | 0.73 | 223 (0.3) | 5 (0.2) |
| Breed | Miniature Schnauzer | 0.7 | [0.4, 1.2] | 0.21 | 867 (1.0) | 13 (0.6) |
| Breed | Shih Tzu | 0.6 | [0.4, 1.0] | 0.03 | 1663 (2.0) | 22 (1.0) |
| Breed | Dachshund (Standard) | 0.5 | [0.2, 1.1] | 0.07 | 727 (0.9) | 6 (0.3) |
| Breed | Patterdale Terrier | 0.5 | [0.2, 1.1] | 0.10 | 583 (0.7) | 6 (0.3) |
| Breed | Spaniel (Cocker) | 0.5 | [0.4, 0.7] | <0.001 | 5384 (6.5) | 58 (2.7) |
| Breed | Whippet | 0.5 | [0.3, 1.1] | 0.10 | 770 (0.9) | 8 (0.4) |
| Breed | Yorkshire Terrier | 0.5 | [0.3, 0.7] | <0.001 | 2167 (2.6) | 25 (1.2) |
| Breed | Bichon Frise | 0.4 | [0.2, 0.8] | 0.01 | 1029 (1.2) | 9 (0.4) |
| Breed | Border Collie | 0.4 | [0.3, 0.6] | <0.001 | 4543 (5.5) | 52 (2.4) |
| Breed | Jack Russell Terrier | 0.4 | [0.3, 0.5] | <0.001 | 6181 (7.4) | 63 (2.9) |
| Breed | West Highland White Terrier | 0.4 | [0.3, 0.6] | <0.001 | 2646 (3.2) | 36 (1.7) |
| Breed | Border Terrier | 0.3 | [0.2, 0.6] | <0.001 | 2030 (2.4) | 18 (0.8) |
| Breed | Spaniel (English Springer) | 0.3 | [0.2, 0.4] | <0.001 | 3895 (4.7) | 31 (1.4) |
| Breed | Cavalier King Charles Spaniel | 0.2 | [0.1, 0.4] | <0.001 | 1766 (2.1) | 10 (0.5) |
| Breed | Lhasa Apso | 0.2 | [0.1, 0.6] | <0.001 | 1102 (1.3) | 9 (0.4) |
| Sex | Female neutered | 1 |  |  | 28389 (34.1) | 753 (34.8) |
| Sex | Female entire | 0.7 | [0.4, 1.2] | 0.21 | 14296 (17.2) | 229 (10.6) |
| Sex | Male neutered | 1.7 | [1.1, 2.5] | 0.01 | 24900 (30.0) | 718 (33.1) |
| Sex | Male entire | 1.1 | [0.7, 1.8] | 0.78 | 15546 (18.7) | 248 (11.4) |
| Age | [3,6) | 1 |  |  | 23748 (28.6) | 168 (7.8) |
| Age | [6,9) | 5.4 | [3.9, 7.6] | <0.001 | 21953 (26.4) | 653 (30.1) |
| Age | [9,12) | 10.5 | [7.6, 14.6] | <0.001 | 17675 (21.3) | 947 (43.7) |
| Age | [12,15) | 9 | [6.3, 12.8] | <0.001 | 9488 (11.4) | 346 (16.0) |
| Age | [15,18] | 2.7 | [1.2, 6.2] | 0.01 | 1856 (2.2) | 25 (1.2) |

## Supplementary table 19 emmeans for pairwise comparison between sex and neuter status for osteosarcomas.

| **Comparison** | **OR** | **SE** | **Reference value** | **Z ratio** | **95% CI** | **p-value** |
| --- | --- | --- | --- | --- | --- | --- |
| FN - FE within [3,6) | 1.4 | 0,29 | 1 | 1,261 | [0.8, 2.5] | 0.589 |
| FN - MN within [3,6) | 0.6 | 0,203 | 1 | -2,533 | [0.4, 0.9] | 0.055 |
| FN - ME within [3,6) | 0.9 | 0,253 | 1 | -0,276 | [0.6, 1.5] | 0.993 |
| FE - MN within [3,6) | 0.4 | 0,276 | 1 | -3,188 | [0.2, 0.7] | 0.008 |
| FE - ME within [3,6) | 0.6 | 0,314 | 1 | -1,387 | [0.3, 1.2] | 0.507 |
| MN - ME within [3,6) | 1.6 | 0,236 | 1 | 1,883 | [1.0, 2.5] | 0.235 |
| FN - FE within [6,9) | 1.7 | 0,15 | 1 | 3,529 | [1.3, 2.3] | 0.002 |
| FN - MN within [6,9) | 0.8 | 0,097 | 1 | -2,15 | [0.7, 1.0] | 0.135 |
| FN - ME within [6,9) | 1.7 | 0,141 | 1 | 3,692 | [1.3, 2.2] | 0.001 |
| FE - MN within [6,9) | 0.5 | 0,149 | 1 | -4,952 | [0.4, 0.6] | <0.001 |
| FE - ME within [6,9) | 1 | 0,181 | 1 | -0,048 | [0.7, 1.4] | 1.000 |
| MN - ME within [6,9) | 2.1 | 0,14 | 1 | 5,208 | [1.6, 2.7] | <0.001 |
| FN - FE within [9,12) | 1.4 | 0,121 | 1 | 2,679 | [1.1, 1.8] | 0.037 |
| FN - MN within [9,12) | 0.8 | 0,083 | 1 | -2,316 | [0.7, 1.0] | 0.095 |
| FN - ME within [9,12) | 1.5 | 0,114 | 1 | 3,799 | [1.2, 1.9] | 0.001 |
| FE - MN within [9,12) | 0.6 | 0,122 | 1 | -4,233 | [0.5, 0.8] | 0.000 |
| FE - ME within [9,12) | 1.1 | 0,145 | 1 | 0,751 | [0.8, 1.5] | 0.876 |
| MN - ME within [9,12) | 1.9 | 0,115 | 1 | 5,438 | [1.5, 2.3] | <0.001 |
| FN - FE within [12,15) | 1.2 | 0,175 | 1 | 0,8 | [0.8, 1.6] | 0.854 |
| FN - MN within [12,15) | 1 | 0,137 | 1 | -0,286 | [0.7, 1.3] | 0.992 |
| FN - ME within [12,15) | 2.1 | 0,2 | 1 | 3,722 | [1.4, 3.1] | 0.001 |
| FE - MN within [12,15) | 0.8 | 0,183 | 1 | -0,979 | [0.6, 1.2] | 0.761 |
| FE - ME within [12,15) | 1.8 | 0,234 | 1 | 2,583 | [1.2, 2.9] | 0.048 |
| MN - ME within [12,15) | 2.2 | 0,207 | 1 | 3,786 | [1.5, 3.3] | 0.001 |
| FN - FE within [15,18] | 0.5 | 0,542 | 1 | -1,177 | [0.2, 1.5] | 0.641 |
| FN - MN within [15,18] | 0.6 | 0,522 | 1 | -1,031 | [0.2, 1.6] | 0.732 |
| FN - ME within [15,18] | 1.8 | 0,806 | 1 | 0,713 | [0.4, 8.6] | 0.892 |
| FE - MN within [15,18] | 1.1 | 0,525 | 1 | 0,191 | [0.4, 3.1] | 0.998 |
| FE - ME within [15,18] | 3.4 | 0,808 | 1 | 1,501 | [0.7, 16.4] | 0.437 |
| MN - ME within [15,18] | 3 | 0,795 | 1 | 1,399 | [0.6, 14.5] | 0.500 |

# Supplementary Statistical Analysis (n=4)

## Note to readers about the statistical test outputs:

The following section provides the full outputs of the statistical tests conducted in support of the results reported in the main manuscript. These include non-parametric comparisons (e.g., Wilcoxon rank-sum, Kruskal-Wallis, Conover’s post-hoc tests) evaluating differences in age at diagnosis and other relevant variables. These outputs are provided for transparency and to facilitate reproducibility and critical appraisal. While the key findings are already summarized in the main text, this supplementary section allows interested readers to review the detailed statistical evidence.

## Supplementary statistical analysis for Mast cell tumours

### MCT: Ages

| Tumour | Count (n) | Mean ± sd | MedianIQR |
| --- | --- | --- | --- |
| Mast cell tumour | 72,038 | 8.05 ± 2.74 | 8.00 [6.00, 10.00] |

### MCT: Age at diagnosis according to Grade 2-tier

| Grade | Count (n) | Mean ± sd | MedianIQR |
| --- | --- | --- | --- |
| High-Grade | 7,101 | 9.29 ± 2.87 | 9.50 [7.50, 11.25] |
| Low-Grade | 40,466 | 7.81 ± 2.65 | 8.00 [6.00, 9.75] |
| NA | 24,471 | 8.08 ± 2.76 | 8.00 [6.08, 10.00] |


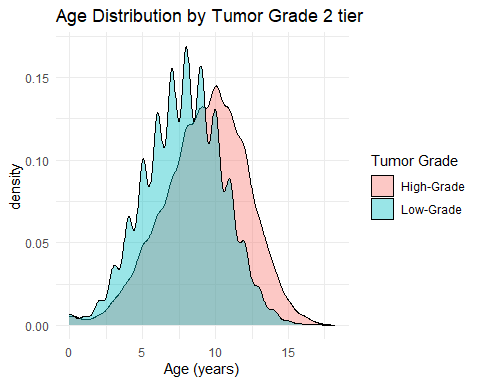


Wilcoxon rank sum test with continuity correction

data: Age_years by Grade_2_tier
W = 187740287, p-value < 2.2e-16
alternative hypothesis: true location shift is not equal to 0

### MCT: Age at diagnosis according to Grade 3-tier

| Grade | Count (n) | Mean ± sd | MedianIQR |
| --- | --- | --- | --- |
| Grade 1 | 2,510 | 7.20 ± 2.60 | 7.17 [5.25, 9.00] |
| Grade 2 | 45,462 | 8.01 ± 2.69 | 8.00 [6.00, 10.00] |
| Grade 3 | 1,664 | 9.53 ± 2.90 | 9.92 [8.00, 11.58] |
| NA | 22,402 | 8.11 ± 2.81 | 8.00 [6.08, 10.00] |

# A tibble: 3 × 2
 Grade_3_tier p_value
 <chr> <dbl>
1 Grade 1 1.16e- 4
2 Grade 2 3.7 e-24
3 Grade 3 3.35e-12

Kruskal-Wallis rank sum test

data: Age_years by Grade_3_tier
Kruskal-Wallis chi-squared = 711.69, df = 2, p-value < 2.2e-16

Conover's test of multiple comparisons : holm

 mean.rank.diff pval
Grade 2-Grade 1 4228.014 <2e-16 ***
Grade 3-Grade 1 12028.301 <2e-16 ***
Grade 3-Grade 2 7800.287 <2e-16 ***
---
Signif. codes: 0 '***' 0.001 '**' 0.01 '*' 0.05 '.' 0.1 ' ' 1

## Supplementary statistical analysis for Melanomas

### Melanoma: Ages.

| Location | Count (n) | Mean ± sd | MedianIQR |
| --- | --- | --- | --- |
| oral cavity | 6,043 | 11.24 ± 2.43 | 11.50 [10.00, 13.00] |
| skin | 3,247 | 9.53 ± 2.82 | 9.92 [7.83, 11.58] |
| digits | 1,263 | 9.72 ± 2.54 | 10.00 [8.00, 11.58] |
| eye and adnexa | 1,040 | 9.26 ± 2.92 | 9.42 [7.08, 11.33] |

# A tibble: 4 × 2
 Location p_value
 <chr> <dbl>
1 digits 2.43e- 8
2 eye and adnexa 1.74e- 6
3 oral cavity 3.7 e-24
4 skin 1.68e-22

Kruskal-Wallis rank sum test

data: Age_years by Location
Kruskal-Wallis chi-squared = 1170, df = 3, p-value < 2.2e-16

Conover's test of multiple comparisons : holm

 mean.rank.diff pval
eye and adnexa-digits -414.0040 0.0055 **
oral cavity-digits 1969.0176 <2e-16 ***
skin-digits -123.2404 0.2414
oral cavity-eye and adnexa 2383.0216 <2e-16 ***
skin-eye and adnexa 290.7635 0.0202 *
skin-oral cavity -2092.2580 <2e-16 ***
---
Signif. codes: 0 '***' 0.001 '**' 0.01 '*' 0.05 '.' 0.1 ' ' 1

## Supplementary statistical analysis for Haemangiosarcomas

### Haemangiosarcoma: Ages.

| - Diagnosis | n | Mean ± sd | MedianIQR |
| --- | --- | --- | --- |
| Splenic HSA | 4,255 | 9.92 ± 2.03 | 10.00 [8.75, 11.17] |
| Cutaneous HSA | 2,662 | 9.21 ± 2.92 | 9.58 [7.50, 11.25] |

Shapiro-Wilk normality test

data: Hemangiosarcoma_Age_test$Age_years[Hemangiosarcoma_Age_test$Diagnosis == "Cutaneous HSA"]
W = 0.98273, p-value < 2.2e-16

Shapiro-Wilk normality test

data: Hemangiosarcoma_Age_test$Age_years[Hemangiosarcoma_Age_test$Diagnosis == "Splenic HSA"]
W = 0.97627, p-value < 2.2e-16


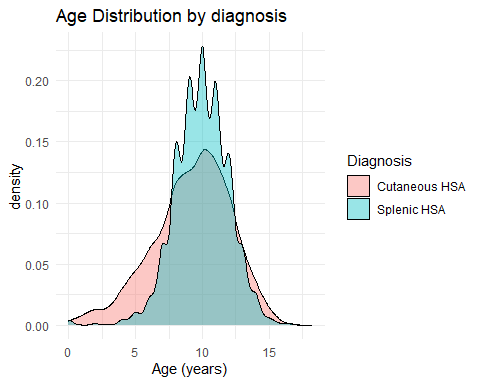


Wilcoxon rank sum test with continuity correction

data: Age_years by Diagnosis
W = 4952618, p-value < 2.2e-16
alternative hypothesis: true location shift is not equal to 0

## Supplementary statistical analysis for Osteosarcomas

### Osteosarcoma: Analyzing differences in the anatomical distribution of osteosarcomas between high-risk and low-risk breeds.

- Pearson's Chi-squared test

  data: chi_matrix
  X-squared = 237.32, df = 35, p-value < 0.0001

### Osteosarcoma: Ages.

| Variable | Overall N =787 | Breed | | P-value |
| --- | --- | --- | --- | --- |
|  |  | High-risk breed N =457 | Low-risk breed N =330 |  |
| Age_years | 9.00 (7.21; 11.00) | 8.08 (6.83; 10.00) | 10.04 (8.50; 12.00) | <.0001 |

### Osteosarcoma: Analizing differences in age at diagnosis in low and high-risk breeds.

Shapiro-Wilk normality test

data: Bone_tumours_Age_test_high_low_risk_breeds_test$Age_years[Bone_tumours_Age_test_high_low_risk_breeds_test$Breed == "High-risk breed"]
W = 0.98077, p-value = 9.488e-06

Shapiro-Wilk normality test

data: Bone_tumours_Age_test_high_low_risk_breeds_test$Age_years[Bone_tumours_Age_test_high_low_risk_breeds_test$Breed == "Low-risk breed"]
W = 0.97234, p-value = 5.868e-06

Wilcoxon rank sum test with continuity correction

data: Age_years by Breed
W = 29667, p-value < 0.0001
alternative hypothesis: true location shift is not equal to 0


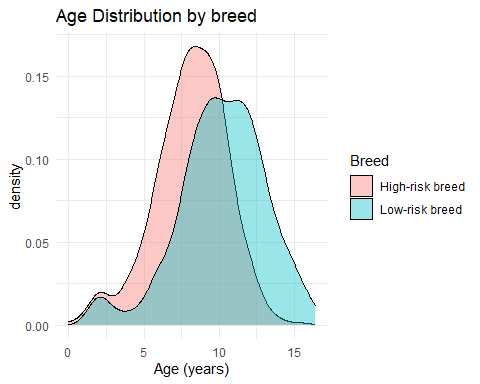


# Supplementary Code

## Python code: see Supplementary_material_(Python script_ANNONIMIZED_TR_paper.html)

# Schematic overview of Data extraction (four steps) and normalization process.

## Free-text narratives examples from the original dataset.

| Report_ID | Narrative |
| --- | --- |
| 78 | <br>diagnosis<br>1) fibroadnexal dysplasia and furunculosis, head<br>2) subcutaneous mct (well, differentiated), thigh<br>3) cutaneous mct, grade 2 (patnaik grading system)/ low-grade mct two-tier grading system (kiupel), upper thorax<br><br>prognosis<br>1) excellent<br>2) good<br>3) fair<br><br>margins<br>1) clear<br>2) <font>lateral margins are clear by 4mm and 2.36mm respectively and the deep margin is clear by 5.26mm</font><br>3) lateral margins are clear b… |
| 157 | <br>diagnosis<br>1) cutaneous mast cell tumour, grade 3 (patnaik grading system)/ high-grade mast cell tumour two-tier grading system (kiupel)<br>2) cutaneous mast cell tumour, grade 3 (patnaik grading system)/ high-grade mast cell tumour two-tier grading system (kiupel)<br>3) cutaneous mast cell tumour, grade 2 (patnaik grading system)/ low-grade mast cell tumour two-tier grading system (kiupel)<br>4) cutaneous mast cell tumour, grade 3 (patnaik grading system)/ high-grade mast cell tumour two-tier grading system (kiupel)<br>… |
| 1693 | <br>diagnosis<br>1) white suture nylon buccal side fibrous reactive proliferation with edema<br>2) purple suture vicryl lingual rostral malignant melanoma<br>3) no suture lingual caudal malignant melanoma<br>4) tooth 405 malignant melanoma with fibrous reactive proliferation<br><br>prognosis<br>poor<br><br>clinical history<br>halitosis swelling lower right side associated with first pre molar 405 very hyperplastic tissue - burst abscess… |
| 2502 | diagnosis<br>ocular melanoma - eye<br>mixed cell infiltration and focal lymphoid cell proliferation - lip<br>fibroadnexal hamartoma and superimposed inflammation - right thigh<br><br>histology<br>the submissions from rocky are 3 samples.<br><br>in the eye, a black intraocular mass was recognized grossly.&#160;&#160;hist |
| 3000 | <br><br>diagnosis haemangiosarcoma&nbsp;&nbsp;<br><br>histology <br>history: ruptured splenic mass. <br><br>description: two sections of the piece of spleen from toby are examined. they contain a neoplastic cellular proliferation with extensive areas of haemorrhage and necrosis. the proliferation consists of variably sized blood filled clefts and channels that are lined by spindle to polygonal cells with abundant dark eosinophilic cytoplasm and oval nuclei with finely stippled chromatin and small nucleoli. there are scattered mitoses.&nbsp;&nb |
| 8699 | <br>diagnosis&nbsp;&nbsp; <br>oral osteoblastic osteosarcoma<br><br>histology&nbsp;&nbsp; <br>history: oral mass<br><br>description: one specimen is submitted and evaluated. it shows the presence of a nonencapsulated and infiltrative tumour in the oral mucosa with extensive mucosal epithelial ulceration. central areas of bone formation and calcification is surrounded by polygonal and spindle shaped neoplastic osteoblasts. the cells contain a large amount of eosinophilic cytoplasm with indistinct cellular borders |

## Step 1: Separating Diagnosis section (Python script_ANNONIMIZED_TR_paper, step 4)

| Report_ID | Diagnosis_narrative |
| --- | --- |
| 78 | <br>diagnosis<br>1) fibroadnexal dysplasia and furunculosis, head<br>2) subcutaneous mct (well, differentiated), thigh<br>3) cutaneous mct, grade 2 (patnaik grading system)/ low-grade mct two-tier grading system (kiupel), upper thorax<br><br |
| 157 | <br>diagnosis<br>1) cutaneous mast cell tumour, grade 3 (patnaik grading system)/ high-grade mast cell tumour two-tier grading system (kiupel)<br>2) cutaneous mast cell tumour, grade 3 (patnaik grading system)/ high-grade mast cell tumour two-tier grading system (kiupel)<br>3) cutaneous mast cell tumour, grade 2 (patnaik grading system)/ low-grade mast cell tumour two-tier grading system (kiupel)<br>4) cutaneous mast cell tumour, grade 3 (patnaik grading system)/ high-grade mast cell tumour two-tier grading system (kiupel)<br>… |
| 1693 | <br>diagnosis<br>1) white suture nylon buccal side fibrous reactive proliferation with edema<br>2) purple suture vicryl lingual rostral malignant melanoma<br>3) no suture lingual caudal malignant melanoma<br>4) tooth 405 malignant melanoma with fibrous reactive proliferation |
| 2502 | diagnosis<br>ocular melanoma - eye<br>mixed cell infiltration and focal lymphoid cell proliferation - lip<br>fibroadnexal hamartoma and superimposed inflammation - right thigh<br><br> |
| 3000 | <br><br>diagnosis haemangiosarcoma&nbsp;&nbsp;<br><br> |
| 8699 | <br>diagnosis&nbsp;&nbsp; <br>oral osteoblastic osteosarcoma< |

## Step 2: Separating individual diagnosis (Python script_ANNONIMIZED_TR_paper, step 5)

| Report_ID | Diagnosis_1 | Diagnosis_2 | Diagnosis_3 | Diagnosis_4 |
| --- | --- | --- | --- | --- |
| 78 | 1) fibroadnexal dysplasia and furunculosis, head | 2) subcutaneous mct (well, differentiated), thigh | 3) cutaneous mct, grade 2 (patnaik grading system)/ low-grade mct two-tier grading system (kiupel), upper thorax |  |
| 157 | 1) cutaneous mast cell tumour, grade 3 (patnaik grading system)/ high-grade mast cell tumour two-tier grading system (kiupel) | 2) cutaneous mast cell tumour, grade 3 (patnaik grading system)/ high-grade mast cell tumour two-tier grading system (kiupel)< | 3) cutaneous mast cell tumour, grade 2 (patnaik grading system)/ low-grade mast cell tumour two-tier grading system (kiupel) | 4) cutaneous mast cell tumour, grade 3 (patnaik grading system)/ high-grade mast cell tumour two-tier grading system (kiupel |
| 1693 | 1) white suture nylon buccal side fibrous reactive proliferation with edema | purple suture vicryl lingual rostral malignant melanoma | no suture lingual caudal malignant melanoma | tooth 405 malignant melanoma with fibrous reactive proliferation |
| 2502 | ocular melanoma - eye | mixed cell infiltration and focal lymphoid cell proliferation - lip |  |  |
| 3000 | haemangiosarcoma&nbsp;&nbsp |  |  |  |
| 8699 | oral osteoblastic osteosarcoma |  |  |  |

## Step 3: Dataset from wider to larger to go from an animal-per row dataset to a diagnosis-per row dataset and use of specific curated dictionaries: match with Keys and mapp to Values (Python script_ANNONIMIZED_TR_paper, step 6 onwards).

| Report ID | Diagnosis ID | Diagnosis | Location | Grade_3_tier | Grade_2_tier |
| --- | --- | --- | --- | --- | --- |
| 78 | 78-2 | mct | thigh |  |  |
| 78 | 78-3 | mct | thorax | grade 2 | low-grade |
| 157 | 157-1 | mast cell tumour | cutaneous | grade 3 | high-grade |
| 157 | 157-2 | mast cell tumour | cutaneous | grade 3 | high-grade |
| 157 | 157-3 | mast cell tumour | cutaneous | grade 2 | low-grade |
| 157 | 157-4 | mast cell tumour | cutaneous | grade 3 | high-grade |
| 1693 | 1693-2 | melanoma | lingual |  |  |
| 1693 | 1693-3 | melanoma | lingual |  |  |
| 1693 | 1693-4 | melanoma | tooth |  |  |
| 2502 | 2502-1 | melanoma | eye |  |  |
| 3000 | 3000-1 | haemangiosarcoma | splenic |  |  |
| 8699 | 8699-2 | osteosarcoma | oral |  |  |

### Dictionaries

| Diagnosis dictionary | |
| --- | --- |
| Keys | Values |
| mct | Mast cell tumour |
| mast cell tumour | Mast cell tumour |
| melanoma | Melanoma |
| haemangiosarcoma | Haemangiosarcoma |
| osteosarcoma | Osteosarcoma |

| Location dictionary | |
| --- | --- |
| Keys | Values |
| cutaneous | Mammary gland |
| subcutaneous | Skin |
| ocular | Eye |
| splenic | Spleen |
| oral | Oral cavity |
| lingual | Oral cavity |
| thigh | Hindlimb |
| thorax | Thorax |
| head | Head |
| tooth | Oral cavity |

| Grade_2_tier dictionary | |
| --- | --- |
| Grade_2_tier | Grade_2_tier |
| Keys | Values |
| high-grade | High-Grade |
| low-grade | Low-Grade |
| grade low to intermediate | Low-Grade |
| low grade | Low-Grade |
| grade high | High-Grade |
| high grade | High-Grade |
| grade low | Low-Grade |

| Grade_3_tier dictionary | |
| --- | --- |
| Grade_3_tier | Grade_3_tier |
| Keys | Values |
| grade 1 | Grade 1 |
| grade one | Grade 1 |
| grade three | Grade 3 |
| grade two | Grade 2 |
| grade 3 | Grade 3 |
| grade 2 | Grade 2 |
| grade iii | Grade 3 |

## Step 4 (end result): Normalized dataset with the mapped terms from the curated dictionaries.

| Report ID | Tumour ID | Diagnosis | Location | Grade_3_tier | Grade_2_tier | Target diagnosis |
| --- | --- | --- | --- | --- | --- | --- |
| 78 | 78-2 | Mast cell tumour | Hindlimb |  |  | Mast cell tumour |
| 78 | 78-3 | Mast cell tumour | Thorax | Grade 2 | Low-Grade | Mast cell tumour |
| 157 | 157-1 | Mast cell tumour | Skin | Grade 3 | High-Grade | Mast cell tumour |
| 157 | 157-2 | Mast cell tumour | Skin | Grade 3 | High-Grade | Mast cell tumour |
| 157 | 157-3 | Mast cell tumour | Skin | Grade 2 | Low-Grade | Mast cell tumour |
| 157 | 157-4 | Mast cell tumour | Skin | Grade 3 | High-Grade | Mast cell tumour |
| 1693 | 1693-2 | Melanoma | Oral cavity |  |  | Oral melanoma |
| 1693 | 1693-3 | Melanoma | Oral cavity |  |  | Oral melanoma |
| 1693 | 1693-4 | Melanoma | Oral cavity |  |  | Oral melanoma |
| 2502 | 2502-1 | Melanoma | Eye |  |  | Uveal melanoma |
| 3000 | 3000-1 | Haemangiosarcoma | Spleen |  |  | Spleen haemangiosarcoma |
| 8699 | 8699-2 | Osteosarcoma |  |  |  | Osteosarcoma |
