# Supplementary material for: Epidemiology of Four Major Canine Tumours in the UK: Insights From a National Pathology Registry With Comparative Oncology Perspectives
Source: Vet Comp Oncol. 2026 Feb 24;24(2):324–40. doi: 10.1111/vco.70056 (PMC13161743; doi:10.1111/vco.70056)
Supplement: Supplementary file 2 — Data S2: Supporting Information. [file VCO-24-324-s001.pdf]

Step 1: Loading data

In [1]:
```
# Load ANNONIMIZED_lab_data_raw
Pathology_records_raw = pd.read_csv("ANNONIMIZED_lab_data_raw.csv")

# Load the reference list spreadsheet
Reference_lists_spreadsheet = pd.read_excel("ANNONIMIZED_lab_data_dictionaries.xlsx
```

Step 2: Different operations to:

a) filter species of interest

b) main text is turned to lowercase to avoid mistmatches among same string in upper and lowercase

c) Cytology cases are removed from the dataset

In [ ]:
```
# Filter Species of interest
Pathology_records_raw = Pathology_records_raw.query('Specie == "Canine"')

# Identify duplicate DiagnosisID rows
duplicates = Pathology_records_raw.duplicated(subset="Report_ID", keep=False)

## Cytology cases are removed from the dataset
Pathology_records_raw = Pathology_records_raw.query('Histo_cyto == "HIST"')

# Keep "Histo" rows if they exist, otherwise keep "Cyto" rows
Pathology_records_raw = Pathology_records_raw.drop_duplicates(subset="Report_ID")
```

Step 3: Converting Excel columns of the Dictionary spreadsheet into Python dictionaries (examples of how dictionaries look like are shown on the Supplementary material (Schematic overview of Data extraction (four steps) and normalization process, Step 3).

In [ ]:
```
# Key lists imported from the Reference_lists_spreadsheet (see chunk 2).
Tumour_list_keys = Reference_lists_spreadsheet["Tumour_list_keys"]
```

```
Grade_keys=Reference_lists_spreadsheet["Grade_keys"].astype(str).str.lower()
Tumour_location_keys = Reference_lists_spreadsheet["Tumour_location_keys"].astype(s
Breeds_keys=Reference_lists_spreadsheet["Breeds_keys"].astype(str).str.lower()

# Value lists imported from the Reference_lists_spreadsheet (see chunk 2).
Tumour_list_keys_values = Reference_lists_spreadsheet["Tumour_list_keys_values"]
Grade_values = Reference_lists_spreadsheet["Grade_values"]
Tumour_location_values = Reference_lists_spreadsheet["Tumour_location_values"]
Breeds_values = Reference_lists_spreadsheet["Breeds_values"]

# Convert key lists to Python lists
Tumour_list_keys = Tumour_list_keys.tolist()
Tumour_location_keys = Tumour_location_keys.tolist()
Grade_keys = Grade_keys.tolist()
Breeds_keys = Breeds_keys.tolist()

# Convert value lists to Python lists
Tumour_list_values = Tumour_list_values.tolist()
Tumour_location_values = Tumour_location_values.tolist()
Grade_values=Grade_values.tolist()
Breeds_values=Breeds_values.tolist()

# This dictionary contains names of tumours.
# There are three Tumour_lists (Tumour_list_keys, Tumour_list_02_keys and Tumour_li
# ...different kind of tumours. There are 3 lists and not just 1 because tumours ha
# ...in such a way that more specific names (such as Osteosarcoma, Carcinosarcoma) 
# ...more generic terms (such as carcinoma, tumour) are on Tumour_list_03_keys.

Dictionary_mapping_diagnosis_keys = []
Dictionary_mapping_diagnosis_keys.extend(Tumour_list_keys)
Dictionary_mapping_diagnosis_values = []
Dictionary_mapping_diagnosis_values.extend(Tumour_list_values)

# This dictionary contains anatomical locations.
Dictionary_mapping_tumour_location_keys = []
Dictionary_mapping_tumour_location_keys.extend(Tumour_location_keys)
Dictionary_mapping_tumour_location_values = []
Dictionary_mapping_tumour_location_values.extend(Tumour_location_values)

# This dictionary contains breeds.
Dictionary_mapping_breeds_keys = []
Dictionary_mapping_breeds_keys.extend(Breeds_keys)
Dictionary_mapping_breeds_values = []
Dictionary_mapping_breeds_values.extend(Breeds_values)

# This dictionary contains grades.
Dictionary_mapping_grade_keys = []
Dictionary_mapping_grade_keys.extend(Grade_keys)
Dictionary_mapping_grade_values = []
Dictionary_mapping_grade_values.extend(Grade_values)


# Once all the lists area created, they are converted to the final Dictionaries.
Dictionary_mapping_diagnosis = dict(zip(Dictionary_mapping_diagnosis_keys, Dictiona
Dictionary_mapping_tumour_location = dict(zip(Dictionary_mapping_tumour_location_ke
Dictionary_mapping_breeds = dict(zip(Dictionary_mapping_breeds_keys, Dictionary_map
```

```
Dictionary_mapping_grade = dict(zip(Dictionary_mapping_grade_keys, Dictionary_mappi
```

Step 4: The "Narratives" column contains the free-text data. From here, using the below regular expression (regex), we extract the specific information describing the tumour type into a new column named "Diagnosis_narrative"

In [2]:

```
# Extract the diagnosis information between "diagnosis" and the next occurrence of

diagnosis_info = Pathology_records_raw['Narratives'].str.extract(r'(?i)(?:diagnos|i

# Combine the extracted information into a single string per row
Pathology_records_raw['Diagnosis_narrative'] = diagnosis_info.groupby(level=0).agg(
```

Step 5: A regex delimiter is used on the newly created "Diagnosis_narrative" column to separete potential different diagnosis within the same report.

Everytime the pattern described by the regex is matched within the same cell in the "Diagnosis" column a split happens.

For instance, a report describing two tumours in "Site 1: ..." and "Site 2:..." will be splitted in two parts and each of these parts will be dinamically written are written in a new column called Diagnosis_1 (Site 1: ...), Diagnosis_2 (Site 2: ...), and so forth.

In [3]:

```
# Function to extract narratives using a delimiter

def extract_narratives(narrative, delimiter, max_columns=30):
    # Check if the input is NaN or None
    if pd.isnull(narrative):
        return [""]  # Return an empty string for NaN values

    # Ensure input is a string
    if not isinstance(narrative, str):
        narrative = str(narrative)
```

```
    # Apply regex split
    narratives = re.split(delimiter, narrative)

    # Keep all elements, including the first one
    if len(narratives) > 1:
        return [n.strip() for n in narratives[:max_columns] if n.strip()]

    return [narrative.strip()]


# Specify the delimiter
delimiter = r'(br>|(?<=\s)\d+\.(?=\s)|site\s\d+|\d+\))'

# Extract narratives using the delimiter and create new columns dynamically
Separate_diagnosis_columns = Pathology_records_raw['Diagnosis_narrative'].apply(lam

# Rename the new columns
Separate_diagnosis_columns = Separate_diagnosis_columns.rename(columns=lambda x: f'

# Concatenate the new columns with the original dataframe
Pathology_records_raw = pd.concat([Pathology_records_raw, Separate_diagnosis_column
```

Step 6: The newly created diagnosis columns (Diagnosis_1, Diagnosis_2, etc.) are reshaped from a wide to a long format. Each diagnosis is transformed into a separate row, retaining the original report identifier (Report_ID). A unique DiagnosisID is generated for each row by combining the report ID (for instance (Report_ID: 78) with the diagnosis number (e.g., DiagnosisID: 78-1), ensuring traceability of multiple diagnoses per report.

In [ ]:
```
# List of diagnosis columns
diagnosis_cols = [col for col in Narratives_ID.columns if 'Diagnosis_' in col]

# Reshape the dataframe
Pathology_records_raw = Pathology_records_raw.melt(id_vars='Report_ID', value_vars=

# Remove 'Diagnosis' from 'DiagnosisID' and prepend Report_ID
Pathology_records_raw['DiagnosisID'] = Pathology_records_raw['Report_ID'].astype(st
```

Step 7: Same operation to step 5 to create a column called "Clinical_history" that will be

used as a source of information for tumour locations.

In [19]:
```
# Define regex pattern
regex_pattern = r'clinical(.*?)(?:comment|histology|$)'

# Extract data using the regex pattern
extracted = Pathology_records_raw['Narratives'].str.extractall(regex_pattern)[0]

# Group by the original DataFrame index and combine the extracted text
Pathology_records_raw['Clinical_history'] = (
    extracted.groupby(level=0).apply(lambda x: ' '.join(map(str, x))).str.strip()
)
```

Step 8: The Tumour_list_keys list (that includes the tumour terms to be matched on the narratives) is used across the "Diagnosis_narrative" column looking for matches of tumours on this column.

Every time a tumour is found through an exact-match is written in a newly created column "Diagnosis".

In [5]:
```
# Define the function to assign labels for Tumour_list

def match_diagnoses(diagnosis_str):
    # Convert the diagnosis_str to lowercase
    diagnosis_str = diagnosis_str.lower()
    # Check if the diagnosis string is in Tumour_list_clean_01 and return the match
    return [diagnosis for diagnosis in Tumour_list_keys if diagnosis in str(diagnos

# get the start time
st_tumour_list_1 = time.time()

# Apply the function to the 'Diagnosis' column

Pathology_records_raw['Matches'] = Pathology_records_raw['Diagnosis_narrative'].app

Pathology_records_raw = Pathology_records_raw['Matches'].apply(pd.Series)

Pathology_records_raw = pd.concat([ID_Tumour_list, match_df], axis=1)

Pathology_records_raw = Pathology_records_raw.drop("Matches", axis=1)

Pathology_records_raw = Pathology_records_raw[Pathology_records_raw['Diagnosis'].no
```

Step 9: Same principle is applied for extract additional information from the narratives such as location, grade, additional features, etc.

An specific diagnosis is used over the dataset to extract specific information and write on a newly created column.

Location is commonly indicated in "Diagnosis" or "Clinical_history" (Step 7) so the the code first searches in "Diagnosis" and, If no location is found, it then looks in "Clinical_history".

Step 10: Adding geographical location to the narratives.

In [1]:

```
# Here we mapp the postcodes of the veterinary practices to rural\urban postcodes.
# https://geoportal.statistics.gov.uk/datasets/55ee8d6742574178ae5fb47e304703e6/abo
# ONS Postcode Directory (November 2023)
# https://www.ons.gov.uk/methodology/geography/geographicalproducts/ruralurbanclass

ONSPD_NOV_2023_UK=pd.read_csv("ONSPD_NOV_2023_UK/ONSPD_NOV_2023_UK.csv")
Rural_Urban_PCD = ONSPD_NOV_2023_UK[['pcd','ru11ind']].rename(columns={'pcd':'Postc

# Merge the two DataFrames on the 'pcd' column
merged_data = pd.merge(Pathology_records_raw, Rural_Urban_PCD, how='left', left_on=

# Create a new column 'Rural_urban' based on the mapping rules
conditions = [
    merged_data['Postcode_ru11ind'].isin(['A1', 'B1', 'C1', 'C2', '1', '2']),
    merged_data['Postcode_ru11ind'].isin(['D1', 'D2', 'E1', 'E2', 'F1', 'F2', '3',
]
choices = ['Urban', 'Rural']

merged_data['Rural_urban'] = np.select(conditions, choices, default='UNKNOWN')

Pathology_records_raw = merged_data

Dictionary_mapping_postcodes = dict(zip(ONSPD_NOV_2023_UK['pcd'], ONSPD_NOV_2023_UK

Pathology_records_raw['Postcode_ru11ind'] = Pathology_records_raw['Postcode'].map(D

# Read the spreadsheet into a pandas DataFrame
df = pd.read_excel("postcode_area_names.xlsx")
```

```
# Create an empty dictionary to store the postcode data
postcode_data = {}

from itertools import takewhile

# Iterate over each row in the DataFrame and populate the dictionary
for index, row in df.iterrows():
    postcode_area = row["Postcode Area"]
    area_name = row["Postcode Area Name"]
    region = row["Region"]
    postcode_data[postcode_area] = {"area_name": area_name, "region": region}

# Create a function to map postcodes to area names and regions
def map_postcode(postcode):
    if isinstance(postcode, str):  # Check if the value is a string
        postcode_letters = ''.join(takewhile(str.isalpha, postcode))  # Extract cha
        if postcode_letters in postcode_data:
            return postcode_data[postcode_letters]['area_name'], postcode_data[post
    return None, None  # Return None for non-string values or unmatched postcodes

# Apply the map function to create new columns
Pathology_records_raw['Area_name'], Pathology_records_raw['Region'] = zip(*Patholog

# OAs to LSOAs to MSOAs to LEP to LAD (May 2022) Lookup in England
# https://geoportal.statistics.gov.uk/datasets/56dfa94d126548b48ceb7bfdd67fb11e/exp
PCD_LAU = pd.read_csv("ONSPD_NOV_2023_UK/PCD_OA_LSOA_MSOA_LAD_MAY22_UK_LU.csv", enc
PCD_LAU = PCD_LAU.rename(columns={'pcd7':'Postcode', 'oa11cd':'OA11CD'})

#Output Area to LAU2 to LAU1 to NUTS3 to NUTS2 to NUTS1 (December 2011) Lookup in E
#https://www.data.gov.uk/dataset/41d50f23-42b8-44ad-99d1-19ee19fd9106/output-area-t
NUTS = pd.read_csv("ONSPD_NOV_2023_UK/Output_Area_to_LAU2_to_LAU1_to_NUTS3_to_NUTS2

merged_data = pd.merge(Pathology_records_raw, PCD_LAU, how='left', left_on='Postcod
Pathology_records_raw = pd.merge(merged_data, NUTS, how='left', left_on='OA11CD', r
```

Step 11: Finally, a list of terms is created to exclude non-diagnostic cases.

In this sense, all reports containing the strings written on the below list negation_terms such as "no evidence", "no neoplas", "no tumo", "no clear evidence" and so forth are removed from the tumour registry.

In [ ]:

```
# Define a list of keywords to check for in the 'Diagnosis' column
negation_terms = [
    "no evidence", "no neoplas", "no tumo", "no clear evidence", "no remain", "inco
    "no residual neoplas", "free from", "non-diagnost", "non diagnos",
    "no evidence to suggest inflammation, infection or neoplasia", "not really have
```

```
]

# Create a boolean mask for rows where the 'Diagnosis' column contains any of the n
uncertain_mask = Pathology_records_raw["Diagnosis_narrative"].str.contains('|'.join

# Remove rows with negation terms from the main dataframe
Pathology_records_raw = Pathology_records_raw[~uncertain_mask]
```

Step 12: Finally, the new dataframe is save into a csv file.

In [ ]:
```
Pathology_records_raw.to_csv("ANNONIMIZED_PTR.csv")
```
